# Supplementary material for: Artificial photosynthetic cells with biotic–abiotic hybrid energy modules for customized CO2 conversion
Source: Nat Commun. 2023 Oct 25;14:6783. doi: 10.1038/s41467-023-42591-x (PMC10600252; doi:10.1038/s41467-023-42591-x)
Supplement: Supplementary file 1 — Supplementary Information [file 41467_2023_42591_MOESM1_ESM.pdf]

Supplementary information

Artificial photosynthetic cells with biotic–abiotic hybrid energy modules for customized CO_2 conversion

Supplementary Information for
Artificial photosynthetic cells with biotic–abiotic hybrid energy modules for customized CO_2 conversion

Feng Gao[1], Guangyu Liu[1], Aobo Chen[1], Yangguang Hu[1], Huihui Wang[1], Jiangyuan Pan[1], Jinglei Feng[1], Hongwei Zhang[1], Yujie Wang[1], Yuanzeng Min[1], Chao Gao [1]* & Yujie Xiong[1,2,3]*

[1]Hefei National Research Center for Physical Sciences at the Microscale, School of Chemistry and Materials Science, National Synchrotron Radiation Laboratory, School of Nuclear Science and Technology, University of Science and Technology of China, Hefei, Anhui 230026, China.

[2]Institute of Energy, Hefei Comprehensive National Science Center, 350 Shushanhu Rd., Hefei, Anhui 230031, China.

[3]Anhui Engineering Research Center of Carbon Neutrality, College of Chemistry and Materials Science, Key Laboratory of Functional Molecular Solids, Ministry of Education, Anhui Normal University, Wuhu, Anhui 241002, China.

These authors contributed equally: Feng Gao, Guangyu Liu.

*e-mail: gaoc@ustc.edu.cn; yjxiong@ustc.edu.cn

Supplementary Methods

Materials. $CdCl_2$, NAD^+, $NADP^+$, NADH, NADPH, 3-(N-morpholino)propanesulfonic acid (MOPS), sodium L-ascorbate, mercaptopropionic acid (MPA), phosphotungstic acid, 2- (N-Morpholino)ethanesulfonic acid (MES), 2,6-dichlorophenolindophenol sodium salt (DCPIP) and $K_3Fe(CN)_6$ were purchased from Aladdin (Shanghai, China). Phosphate-buffered saline (PBS), 4-(2-hydroxyethyl)piperazine-1-ethanesulfonic acid (HEPES), BCA kit, ATP assay kit, sodium dodecyl sulfate-polyacrylamide gel electrophoresis (SDS-PAGE) kit, Blue Native-polyacrylamide gel electrophoresis (BN-PAGE) kit, bovine serum albumin, ADP, DNase, RNase, protease inhibitor, tricine and sorbitol were purchased from Beyotime Biotechnology (Shanghai, China). ATP was purchased from meilunbio (Dalian, China). Percoll was purchased from Solarbio (Beijing, China). Poly(diallyldimethylammonium chloride) (PDADMAC) (molecular weight <100000, 35 wt.% in H_2O) was purchased from Sigma-Aldrich (Shanghai, China). Glyoxylate, glycolate and 2,2'-bipyridine were purchased from Energy-chemical (Shanghai, China). Dichloro(pentamethylcyclopentadienyl)rhodium (III) dimer was purchased from Bide Pharmatech (Shanghai, China). Dithiothreitol (DTT), sodium dithionite and 1H, 1H, 2H, 2H-perfluoro-1-octanol were purchased from Macklin (Shanghai, China). Fluorinated oil (Novec 7500) was purchased from 3M (Shanghai, China). Genes and primers (Supplementary Table 12) were synthesized by General biol (Anhui, China). DNA polymerase and restriction enzymes were purchased from Thermo Fisher Scientific Inc (Waltham, MA, USA). pET28a (+) and pET22b (+) were purchased from Novagen (Madison, WI, USA). The plasmid isolation kit, Tris-HCl, glycerol and Ni-NTA super flow column for purification were purchased from Sangon Biotech (Shanghai, China). Electrophoresis reagents were purchased from Bio-Rad Laboratories (Hercules, CA, USA). All other reagents were purchased from Sinopharm Chemical Reagent Company (Shanghai, China). All reagents were used as received without further purification unless specified otherwise. The water used in all experiments was purified by using a Millipore Milli-Q water system (resistance was 18.2 MΩ cm).

Preparation of CdTe quantum dots (QDs). MPA-capped CdTe QDs were synthesized according to a previously reported method[1] with slight modification. Briefly, a fresh NaHTe solution was first prepared. Under an argon atmosphere, 0.1 mmol of tellurium powder, 2 mmol of $NaBH_4$ and 10 mL of water were added into a 25 mL three-necked flask. The solution was heated to 90 °C for 10 min to obtain a transparent solution of NaHTe, which was then cooled to ~50 °C for further use.

At the same time, 0.2 mmol of $CdCl_2$, 250 μL of MPA and 20 mL of water were added into a 50 mL three-necked flask, followed by adjusting the pH to 10.0 by dropwise addition of 1.0 M NaOH solution under stirring. The solution was heated to 50 °C under the protection of an Ar atmosphere. After that, 2.0 mL of a freshly prepared NaHTe solution was injected into the solution of Cd precursor, and the solution was heated to 90 °C under reflux and an Ar atmosphere. The growth of QDs in the solution was monitored by fluorescence spectroscopy. When the fluorescence emission peak reached ~585 nm, the heating mantle was removed and the resulting mixture was cooled to room temperature, which yielded the CdTe QDs colloid solution.

Preparation of CdS quantum dots (QDs). MPA-capped CdS QDs were synthesized according to a previously reported method[2] with slight modification. Briefly, 1 mmol of $CdCl_2$, 3.4 mmol of MPA and 20 mL of water were added into a 50 mL three-necked flask, followed by adjusting the pH to 12.0 by dropwise addition of 30 wt. % NaOH solution under stirring. The solution was protected with Ar atmosphere. After that, 1.0 mL of a freshly prepared Na_2S solution (0.1 mmol) was injected into the solution of Cd precursor, and the solution was heated to 100 °C heating mantle under reflux and an Ar atmosphere. Finally, the bright-yellow, transparent solution was stirred for 30 min to promote the growth of QDs. And then heating mantle was removed and the resulting mixture was cooled to room temperature, which yielded the CdS QDs colloid solution. The as-prepared CdS QDs colloid solution was washed with ethanol (v: v= 1:1) and then redispersed in water for further use.

Preparation of MoS_2 quantum dots (QDs). MoS_2 QDs were synthesized according to a previously reported method[3]. Briefly, 250 mg of $Na_2MoO_4·2H_2O$ was dissolved in 25 mL of water and ultrasonication for 5 min, followed by adjusting the pH to 6.5 by dropwise addition of 0.1 M HCl solution under stirring. Subsequently, 500 mg of L-cysteine and 50 mL of water were added with ultrasonication for 10 min. Finally, the obtained mixture was transferred into a Teflon-lined stainless-steel autoclave (100 mL) and reacted at 200 °C for 36 h. After the resultant mixture cooled to room temperature, MoS_2 QDs was collected via centrifugation for 20 min (12, 000 × g).

Functionalization of QDs. The functionalization of CdTe QDs with PDADMAC was achieved by modifying a previously reported protocol[4]. Briefly, the as-prepared CdTe QDs colloid solution was washed with ethanol (v: v= 1:1) and then redispersed in water. 9 mL of 1.3 mg/mL CdTe QDs was mixed with 1 mL of PDADMAC solution (3.5%, in water), followed by stirring for 10 min in the dark. The obtained solution was added with 2-propanol (v/v= 1:3) to precipitate the QDs. After centrifugation, the QDs were redispersed in water. To remove any unbound PDADMAC, the washing procedure was repeated several times. Finally, the resulting positively charged CdTe QDs ($CdTe^+$) were collected and redispersed in SSC buffer (100 mM NaCl and 50 mM sodium citrate, pH 7.8) for further use. The same method was used to obtain other positively charged QDs (i.e., CdS^+ and MoS_2^+).

Preparation of thylakoid. Thylakoid of young spinach was isolated according to a previously reported method[5-7]. The fresh spinach was purchased from the vegetable market. Spinach leaves were stored in the dark at 4 °C one night. Dark adapted spinach leaves were washed, and large stems were removed. The harvested leaves were blended in cold buffer A (330 mM sorbitol, 50 mM HEPES-KOH pH 7.6, 5 mM $MgCl_2$, and 0.1% (w/v) bovine serum albumin). The resulting homogenate was filtered through two layers of nylon cloth, and the filtrate was centrifuged at 3,000 × g for 10 min at 4 °C. The obtained precipitate was re-suspended in cold buffer B (300 mM sorbitol, 50 mM HEPES-KOH pH 7.6, 5 mM $MgCl_2$, and 10 mM sodium L-ascorbate), followed by

gently overlaying on a Percoll gradient (80%:80% v/v Percoll, 10 mM sodium L-ascorbate, 300 mM sucrose, and 66 mM MOPS-KOH pH 7.6; 40%:40% v/v Percoll, 10 mM sodium L-ascorbate, 300 mM sucrose, and 25 mM MOPS-KOH pH 7.6). The fraction containing thylakoid was taken out and diluted with cold buffer B, followed by centrifugation at 3,000 × g for 10 min at 4 °C. The obtained precipitate was re-suspended in cold buffer C (10 mM HEPES-KOH, 10 mM $MgCl_2$, 10 mM sodium L-ascorbate, and 10% DMSO) and stored at −80°C in dark until use.

Before use, thylakoid was washed 2~3 times with cold buffer D (700 mM sorbitol, 10 mM HEPES-KOH pH 7.6, 10 mM $MgCl_2$, and 10 mM sodium L-ascorbate). The chlorophyll (Chl) concentration of thylakoid was determined according to the previously reported method[8]. The intact of the thylakoid was determined by BN–PAGE analysis according to the previously reported method[9].

Isolation of plasma membrane, broken liposome and protein/proteoliposome. The obtained thylakoid precipitate was re-suspended in cold buffer C (10 mM HEPES-KOH, 10 mM $MgCl_2$, 10 mM sodium L-ascorbate, and 10% DMSO) and stored at −80°C in dark until use. Broken plasma membrane was obtained via centrifugation (3,000 × g, 10 min, 4 °C) of the melting and osmotic thylakoid precipitate. To obtain liposome and reconstituted protein, intact thylakoid was solubilized by 2 % DDM, and partial large liposome was collected by centrifugation (12,000 × g, 30 min, 4 °C). Reconstituted protein/proteoliposome was obtained based on complete detergent solubilization method by following previous literature[10-12]. Briefly, intact thylakoid was solubilized by 2 % DDM and incubation for 30 min for equilibration. The DDM was removed at 4 °C by adding Bio-Beads (Bio-Rad) with a slow procedure consisting of 2 sequential additions. 20 mg Bio-Beads per milligram of DDM was added for 1 h, followed by an addition of 30 mg Bio-Beads per milligram of DDM for another 2 h. Reconstituted protein was collected and stored at -80 °C until use.

Preparation of PSII-enriched membrane particles. PSII-enriched membrane particles (PSII) were isolated and purified from young spinach according to a

previously reported method[13]. The fresh spinach was purchased from the vegetable market. Spinach leaves were stored in the dark at 4 °C one night. Dark adapted spinach leaves were washed, and large stems were removed. The harvested leaves were blended in cold buffer A1 (20 mM Tricine-NaOH pH 7.8, 0.4 M sucrose, 2 mM $MgCl_2$, 40 mM NaCl, 2 mM Vitamin C, 0.2% BSA). The resulting homogenate was filtered through two layers of nylon cloth, and the filtrate was centrifuged at 2,000 × g for 1 min at 4 °C to obtain supernatant mixture, which was then centrifuged at 6,000 × g for 15 min at 4 °C to collect chloroplast. The obtained chloroplast was re-suspended in cold buffer B1 (20 mM Tricine-NaOH pH 7.8, 10 mM NaCl, 5 mM $MgCl_2$, 0.2% BSA) with gently stirred for 15 min to break the intact chloroplast. Subsequently, the above mixture was centrifugated at 1,600 × g for 2 min, followed by centrifugation at 8000 × g for 15 min to collect the precipitate. And then the precipitant was resuspended in high ionic strength buffer C1 (0.4 M sucrose, 20 mM MES-NaOH pH 6.5, 15 mM NaCl, 5 mM $MgCl_2$) and meanwhile using buffer C1 to adjust chlorophyll (Chl) concentration (2.5 mg/mL). Buffer D1 (buffer C with 20 wt% TX-100, pH 6.5) was slowly added to the suspension with gently stirring until the weight ratio of TX-100 to Chl is 20:1. Finally, the suspension was centrifugated at 10,000 × g for 1 min, followed by centrifugation at 35,000 × g for 30 min to collect the precipitate. The precipitate was washed with Buffer C1 repeatedly until the supernatant was not green and re-suspended in cold buffer E1 (buffer C with 0.5 M betaine) and stored at -80°C in dark until use.

Preparation of PSII-CdTe. The modification of PSII with $CdTe^+$ was accomplished by mixing the as-prepared PSII with $CdTe^+$ (mass ratio of PSII (Chl) to $CdTe^+$ was 1:1) in a vial with gently stirring for 10 min, followed by centrifugation at 10,000 × g for 10 min at 4 °C to remove unbounded $CdTe^+$, which yielded the $CdTe^+$-modified PSII (namely, PSII-CdTe).

Characterizations. To determine the elemental composition, the sample was dissolved with aqua regia (HCl/HNO_3 = 3:1, volume ratio) and the resulting solution was filtered.

The filtrate was subjected to quantification on metal contents with an inductively coupled plasma atomic emission spectrometry (ICP-AES) (Optima 7300 DV). The reported values are averages of triplicate measurements. The error bars represent standard deviations.

To examine the morphology, a drop of the aqueous suspension of the sample was added onto a piece of the carbon-coated copper grid, dried under ambient conditions, and then observed on a transmission electron microscope (TEM) (Hitachi H-7700 operated at 100 kV, JEM-F200 operated at 200 kV for HRTEM and JEOL JEM-2100F operated at 200 kV for EDS mapping). To examine the bio-membrane morphology, a drop of the resultant dispersion was added onto a copper grid, followed by negatively staining with a solution of phosphotungstic acid (2 wt% in water) (1 drop), drying under ambient conditions and then observation on a transmission electron microscope (TEM) (Hitachi H-7700 operated at 100 kV).

To observe ultrastructure of thylakoids, TEM samples were prepared by a typical process of fixation, dehydration and embedding in a resin matrix according to the reported method. The as-prepared sample was fixed with 5% glutaraldehyde overnight, washed thrice with phosphate buffer (PB, 0.1 M, pH = 7.2), and stained with a solution of 2% osmium tetroxide (1 mL) overnight. In the following step, the sample was rinsed thrice with PB (0.1 M, pH = 7.2) (20 min each, 1 mL), dehydrated in a series of graded ethanol solutions (30, 50, 70, 80, 95 and 100% ethanol in water), and then rinsed thrice with acetone (20 min each, 1 mL). The resulting sample was incubated in graded resin solution (with resin-to-acetone volume ratio at 1:1, 3:1 and 100% resin) overnight. Subsequently, a new batch of resin (250 μL) was added, and the sample was cured at 70 °C. Finally, 70-nm-thick samples were sliced off using an ultramicrotome equipped with a diamond knife (Leica UC7) and placed on copper grids for TEM imaging (Hitachi H-7700 operated at 100 kV).

To monitor the surface zeta-potential, the sample was dispersed into the buffer D, and the resulting dispersion was subjected to dynamic light scattering (DLS) measurement with an analyzer (Zetasizer Nano ZS, Malvern). The reported values are averages of triplicate measurements. The error bars represent standard deviations.

To measure the absorption, the sample was dispersed into the buffer D, and the resulting dispersion was subjected to ultraviolet-visible absorption spectrum recorded with a UV−Vis spectrometer (Cary 60 UV-Vis, Agilent).

To explore the fluorescence, the sample was dispersed into the buffer D, and the resulting dispersion was then subjected to fluorescence emission spectrum recorded ($\lambda_{ex}/\lambda_{em}$ = 400 nm/450–800 nm, slit-width of 10 nm for both excitation and emission wavelength) with a fluorometer (F-4600 spectrofluorometer, Hitachi).

To visualize fluorescence image, the sample was dispersed into the buffer D, and the resulting dispersion was then subjected to fluorescence confocal microscope (SP5, Leica) imaging. For thylakoid, an excitation laser line of 488 nm and an emission wavelength range of 650–740 nm were used. For $CdTe^+$, an excitation laser line of 405 nm and an emission wavelength range of 540–640 nm were used. The reported results have been checked for consistency with 3 individual samples.

Measurements of luminescence lifetime. Luminescence lifetime measurements were carried out using time-correlated single-photon counting system. The lifetime data were analyzed with Data Station v6.6 (Horiba Scientific). The samples (Chl equivalent of 100 μg/mL for Tk, 86.4 μg/mL for CdTe) were excited by the incident light of 370 nm with a 1 MHz LED laser (NanoLED-370). Logarithmic plots of emission decays of CdTe were at 590 nm. The samples (Chl equivalent of 20 μg/mL for Tk or PSII, 17.28 μg/mL for CdTe) were excited by the incident light of 450 nm with a 1 MHz LED laser (NanoLED-450). Logarithmic plots of emission decays of Tk or PSII were at 685 nm. The fluorescent decay curve was fitted with tri-exponential decay fit. Each decay curve was fitted to a three-exponential function: $i(t) = A + B1*\exp(-t/T1) + B2*\exp(-t/T2) + B3*\exp(-t/T3)$.

Assays for electron transfer from PSII. Electron transfer from PSII was evaluated directly through a similar protocol as described above in "Assays for electron transfer from PSII to PSI". Briefly, the dispersion of PSII-CdTe (Chl equivalent of 20 μg/mL or equal amount $CdTe^+$, i.e., Chl equivalent × loading efficacy; similarly treated

hereinafter) in PBS was added into DCPIP solution (100 μM). The resulting mixture was subsequently illuminated with white light at 0.1W/cm^2 for a certain time. The resulting reaction buffer was then centrifuged at 10,000 × g for 10 min at 4 °C. The resultant supernatant was then subjected to measurement of absorption at 595 nm with a microplate reader. Controls were assayed similarly but only with DCPIP in PBS. The reported values are averages of triplicate measurements. The error bars represent standard deviations.

$K_3Fe(CN)_6$ photoreduction assay for verifying the presence of plasma membrane. $K_3Fe(CN)_6$ photoreduction was evaluated directly through a protocol as described in "https://www.sigmaaldrich.cn/deepweb/assets/sigmaaldrich/product/documents/638/446/cpisopis-mk.pdf". Briefly, the dispersion of sample (Chl equivalent of 30 μg/mL) in buffer was added into $K_3Fe(CN)_6$ solution (1 mM). The resulting mixture was illuminated with light at 0.1 W/cm^2 for a certain time (0, 2, 4, 6 min), and subsequently centrifuged at 10,000 × g for 10 min at 4 °C. The obtained supernatant was then subjected to measurement of absorption at 405 nm with a microplate reader. The control experiment were assayed similarly but only with $K_3Fe(CN)_6$. The reported values are averages of triplicate measurements and the error bars represent standard deviations.

Bioactivity assay of NADPH or NADH with ^{1}H NMR. NADPH or NADH regeneration activity was assayed in a 1 mL reaction volume with 15 μg/mL Chl equivalent. The reaction buffer was illuminated with white light (AM 1.5 filter, sunlight simulation) at 0.1 W/cm^2 for 30 min. The resulting reaction buffer was centrifuged at 3,000 × g for 10 min at 4 °C. The resultant supernatant (0.5 mL) was then subjected to ^{1}H NMR spectrum recorded with Bruker AVANCE AV III 400. Controls were assayed similarly but only with NADPH/NADH or $NADP^+$/NAD^+.

Meanwhile, NADPH regeneration activity was assayed in a 0.6 mL reaction volume with 30 μg/mL Chl equivalent and GhrA (6 μg/mL), by adding glyoxylate (3 mM). The reaction buffer was illuminated with white light (AM 1.5 filter, sunlight simulation) at 0.1 W/cm^2 for 30 min. The resulting reaction buffer was centrifuged at

3,000 × g for 10 min at 4 °C. The resultant supernatant (0.5 mL) was then subjected to ^{1}H NMR spectrum recorded with Bruker AVANCE AV III 400. Controls were assayed similarly but only with glyoxylate or glycolate.

Preparation of $[Cp^{*}Rh(bpy)H_2O]^{2+}$. $[Cp^{*}Rh(bpy)H_2O]^{2+}$ was synthesized according to a previously reported method[14]. Briefly, 31.2 mg of 2,2'-bipyridine was added into a suspension of dichloro(pentamethylcyclopentadienyl)rhodium (III) dimer (61.8 mg) in methanol (4 mL), followed by stirring for 12 h. 40 mL of cold diethyl ether was poured into the resultant mixing solution to precipitate [Cp*Rh(bpy)Cl]Cl. After centrifugation, the [Cp*Rh(bpy)Cl]Cl was kept in vacuum to remove diethyl ether. The solid was dissolved in water to prepare stock solutions and stored at 4 °C avoiding direct exposure to light. [Cp*Rh(bpy)Cl]Cl was readily hydrolyzed to $[Cp^{*}Rh(bpy)H_2O]^{2+}$. The [Cp*Rh(bpy)Cl]Cl was subjected to ^{1}H NMR spectrum recorded with Bruker AVANCE AV III 400.

NADH production with addition of $[Cp^{*}Rh(bpy)H_2O]^{2+}$. NADH regeneration activity was assayed in a 1 mL reaction volume with Tk–CdTe (15 or 60 μg/mL Chl equivalent) and $[Cp^{*}Rh(bpy)H_2O]^{2+}$(0.2 or 0.4 mM) added. The reaction buffer was illuminated with white light (AM 1.5 filter, sunlight simulation) at 0.1 W/cm^2 for 30 min. The resulting reaction buffer was centrifuged at 3,000 × g for 10 min at 4 °C. The resultant supernatant (0.5 mL) was then subjected to ^{1}H NMR spectrum recorded with Bruker AVANCE AV III 400.

NADH production with addition of impurities or $K_3Fe(CN)_6$. NADH regeneration was assayed by monitoring the absorption at 340 nm in a 1 mL reaction buffer. The reaction buffer, containing 50 mM 4-(2-hydroxyethyl) piperazine-1-ethanesulfonic acid (HEPES)-KOH with a pH of 7.8, 5 mM K_2HPO_4, 3 mM NAD^+, 10 mM sodium L-ascorbate (not containing sodium L-ascorbate in the $K_3Fe(CN)_6$-added assay to avoid the redox reaction), 10 mM KCl, 5 mM $MgCl_2$ and various samples (Tk, Tk + 0.1 mM $K_3Fe(CN)_6$; impurities, Tk + impurities, the impurities was isolated form the equivalent

Tk), was illuminated with white light (AM1.5 filter, sunlight simulation) at 0.1 W/cm^2 for 10 min. The resulting reaction buffer was centrifuged at 3,000 × g for 10 min at 4 °C. The obtained supernatant was then subjected to measurement of absorption at 340 nm with the spectrophotometer (Metash, V-5000).

Cloning, expression and purification of glyoxylate/hydroxypyruvate reductase (GhrA). The GhrA genes (Supplementary Table 13, Uniprot: P75913) were cloned using NdeI and XhoI (Supplementary Table 14). The cloned genes were confirmed to be free from point mutations by DNA sequencing (General Biology, China). The recombinant plasmids (Supplementary Table 15) were transformed in competent *Escherichia coli* BL21(DE3) cells. Expression and purification of the GhrA were performed according to a previously reported method[15] with modification. Briefly, cells were cultured in medium (Supplementary Table 16) at 37 °C with shaking until the OD_{600} reached 1.0. Then the recombinant protein was induced using 0.2 mM isopropyl-β-D-thiogalactopyranoside (IPTG) at 16 °C for 20 h. The cells were collected by centrifugation at 5,000 × g for 10 min at 4 °C, followed by lysing using a high-pressure cell disrupter for 5 min under 800–1000 bar. The supernatant was harvested by centrifugation at 13,000 × g for 30 min at 4 °C, and then incubated with pre-equilibrated Ni-NTA resins column. The purified protein was concentrated, and the molecular weight was determined by size-exclusion chromatography and SDS–PAGE analysis.

Cloning, expression and purification of formate dehydrogenases CcFDH and PsFDH. The CcFDH (UniProt E2IQB0) and pristine PsFDH (Uniprot P33160) genes were cloned into pET28a (+) and pET22b (+) using *NdeI* and *XhoI* or *NdeI* and *NotI*, respectively. The cloned CcFDH and pristine PsFDH genes were confirmed to be free from point mutations by DNA sequencing (General Biology, China). The amino acid residues determining the cofactor specificity of NADH-FDH were reported to be at 222 and 224 in *Pseudomonas*[16]. To obtain mutational PsFDH (PsFDH), site-directed mutagenesis of the resulting plasmid with the PCR-based QuikChange Method (Agilent Technologies) was carried out to substitute nucleotides corresponding to the

aforementioned pairs of amino acids, which introduced 222 Gln and 224 Asn substitutions into the pristine PsFDH coding sequence.

The recombinant plasmids were transformed in competent *Escherichia coli* BL21(DE3) cells. Expression and purification of the FDHs were performed according to a previously reported method[12] with modification. Briefly, cells were cultured in LB medium at 37 °C with shaking until the OD_{600} reached 0.6–0.8. Then the recombinant CcFDH and PsFDH proteins were induced using 0.1 mM and 0.5 mM IPTG at 16 °C for 20 h, respectively. The cells were collected by centrifugation at 5,000 × g for 20 min at 4 °C, followed by sonication in lysis buffer containing Tris-HCl (pH 7.4), NaCl, DNase, RNase and protease inhibitor for 15 min with ice bath. The supernatant was harvested by centrifugation at 12,000 × g for 30 min at 4 °C, and then incubated with pre-equilibrated Ni-NTA resins column. To obtain pure CcFDH and PsFDH, bound Ni-NTA resins were eluted with 6 mL of wash buffer (50 mM Tris-HCl, pH 7.4, 500 mM NaCl, 50 mM imidazole). The purified proteins were concentrated and stored with buffer (50 mM Tris-HCl pH 7.4, 150 mM NaCl). The purity of the proteins was determined by SDS–PAGE analysis. The concentration of the proteins was determined by BCA kit, with bovine serum albumin as a standard protein[17].

Cloning, expression and crude extract of remodeled nitrogenase MoFe. The nifD gene (UniProt P07328) and nifA gene (UniProt P09570) were cloned into puc19 (+) and puc57-Kan (+) using *BamHI* and *SphI* or *HindIII* and *XbaI*, respectively. The cloned nifD and nifA genes were confirmed to be free from point mutations by DNA sequencing (General Biology China). According to previous studies[18], the amino acid residues determining the substrate selectivity of Mo-Fe nitrogenases were suggested to be at 70 and 195 in α subunit. Therefore, the site-directed mutagenesis of the resulting plasmid using the PCR-based QuikChange Method (Agilent Technologies) was carried out to introduce the 70 Ala and 195 Gln substitutions into the nifD coding sequence. The recombinant plasmids were transformed in competent *Azotobacter Vinelandii* with electroporation system. Expression and crude extract of the MoFe were performed according to a previously reported method[19] with modification. Briefly, cells were

cultured in medium (Supplementary Table 16) at 28 °C with shaking until the OD_{600} reached 0.6–0.8. Then the recombinant MoFe proteins were induced using 0.1 mM IPTG at 16 °C for ~20 h. The cells were collected by centrifugation at 5,000 × g for 20 min at 4 °C, followed by suspending in 4 M glycerol in Tris-HCl buffer (25 mM, pH 7.4) for 30 min. The suspension was added with 10 mM sodium dithionite and purged with N_2 for 10 min, followed by centrifugation at 12,000 × g for 10 min at 4 °C. After the glycerol was discarded, the pellet was re-suspended in lysis buffer containing Tris-HCl (25 mM, pH 7.4), DTT (0.1 mg/mL), DNase (10 μg/mL) and sodium dithionite (10 mM), with N_2 purged for 10 min, followed by sonication for 15 min with ice bath. The supernatant was harvested by centrifugation at 12,000 × g for 30 min at 4 °C. The crude proteins were concentrated by 30 kDa MWCO Amicon Ultra Centrifugal Filters (Millipore). The concentration of the proteins was determined by BCA kit.

Fabrication and operation of microfluidic devices. The microfluidic devices were purchased from ZhongXinQiHeng (Suzhou,China). Chip devices were made of poly(dimethylsiloxane) (PDMS) from a SU8-2000 negative photoresist mold (30 μm depth) produced using a standard soft-lithography procedure. Pressure-driven pump was used to control the flows in the microfluidic channels. Chip devices were connected to controller with polytetrafluoroethylene (PTFE) tubing (inner diameter (ID) of 0.6 mm and an outer diameter (OD) of 1.6 mm). Droplets were produced in fluorinated oil and stabilized against coalescence by a perfluoropolyether–polyethyleneglycol block copolymer surfactant (PFPE–PEG). All microfluidic devices were used at room temperature (24 °C).

Microscopy and fluorescence measurement. To capture real-time brightfield image, microfluidic chips were mounted on the x–y stage of an inverted microscope (WITec Alpha 300R). Droplets were observed with a high-speed camera using white light from the top. Then, the collected emulsions (30 μL) were transferred to a culture dish specifically designed for microscopy. The emulsions were visualized by using white

light from confocal microscopy (SP5, Leica). The fluorescence signals of the emulsions were detected by using a confocal microscopy (SP5, Leica), with an excitation laser line of 405 nm and an emission wavelength range of 410–500 nm for NADH or NADPH. For thylakoid, an excitation laser line of 488 nm and an emission wavelength range of 650–740 nm were used. For $CdTe^+$, an excitation laser line of 405 nm and an emission wavelength range of 540–640 nm were used.

NADPH, NADH and ATP regeneration in artificial photosynthetic cells. Artificial photosynthetic cells were created from "**Fabrication of artificial photosynthetic cells**", and the collected artificial photosynthetic cells with a total volume of 200 μL were illuminated with white light (AM 1.5 filter, sunlight simulation) at 0.1 W/cm^2. Subsequently, 150 μL of fluorinated oil was added to 90 μL of pre-treated artificial photosynthetic cells. 60 μL of 1H, 1H, 2H, 2H-perfluoro-1-octanol was then added, and the resultant mixture was vortexed and centrifuged. The aqueous phase was pipetted and quenched with 1% of HCl. NADPH or NADH regeneration activity was assayed by monitoring the absorption at 340 nm in a 1 mL reaction volume. ATP regeneration activity was measured by using the ATP assay kit.

Electrochemical measurements. Linear sweep voltammetry was performed with a CHI 760E Potentiostat under the atmosphere at ambient temperature. A standard three-electrode cell was used for all measurements. A platinum electrode and an Ag/AgCl electrode were used as the counter and reference electrode, respectively. A glassy carbon electrode (3 mm in diameter) was used as the working electrode where the samples were loaded. After dripping and drying the catalyst dispersion (5 mg of samples, 30 μL of 5 wt% Nafion solution, and 0.5 mL of ethanol solvent) on the glassy carbon electrode, all the electrodes were immersed into an electrolytic solution (0.1 M of KCl, 0.1 M of triethanolamine, and the pH value was adjusted to 7 by using HCl solution). Prior to the measurements, the solvent was purged for at least 15 min with the pure CO_2 gas. All the LSV curves were recorded at a scan rate of 0.005 V s^{-1}. The conduction

band (CB) energy levels were calculated from the onset potentials of reduction. The valence band (VB) energy levels were determined according to the related CB energy levels and band gap (Eg)[2,20,21]. The relationship of E_{VB}, E_{CB}, Eg, E(Ag/AgCl), measured potential (E_{mea}) and normal hydrogen electrode (NHE) were simplified as follows:

E(NHE) = E(Ag/AgCl) + 0.197 V.

$E_{CB} = E_{mea} – [E(NHE) – E(Ag/AgCl)]$

$E_{VB} = E_{CB} + Eg$

E_{mea} corresponds to the onset reduction potential which is obtained from the tangents in LSV. Eg was determined from the UV–Vis diffuse reflectance spectra.

Calculation of internal quantum efficiency. To obtain the internal quantum efficiency of Tk-CdTe, the light absorption property of Tk-CdTe was analyzed under AM1.5 irradiation, by multiplying the photon flux distribution of AM1.5 spectra with the absorption of photons at different wavelengths by Tk-CdTe[22,23]. The absorption of photons at different wavelengths by Tk-CdTe was calculated by following the Beer-Lambert law with the equation below:

$$\text{Absorption (100\%)} = 1 - \text{transmittance} = 1-10^{-\text{absorbance}}$$

The overall absorption percentage of Tk-CdTe was calculated to be 3.54% using the following equation:

$$\text{Absorption percentage of AM1.5} = \frac{\int \text{photons flux absorbed by Tk-CdTe}}{\int \text{incident photons flux}}$$

Internal quantum efficiency (IQE) was calculated as follows:

$$\text{IQE} = \frac{\text{the number of electrons for production of HCOOH}}{\text{total absorbed incident photons}}$$

Supplementary Figures

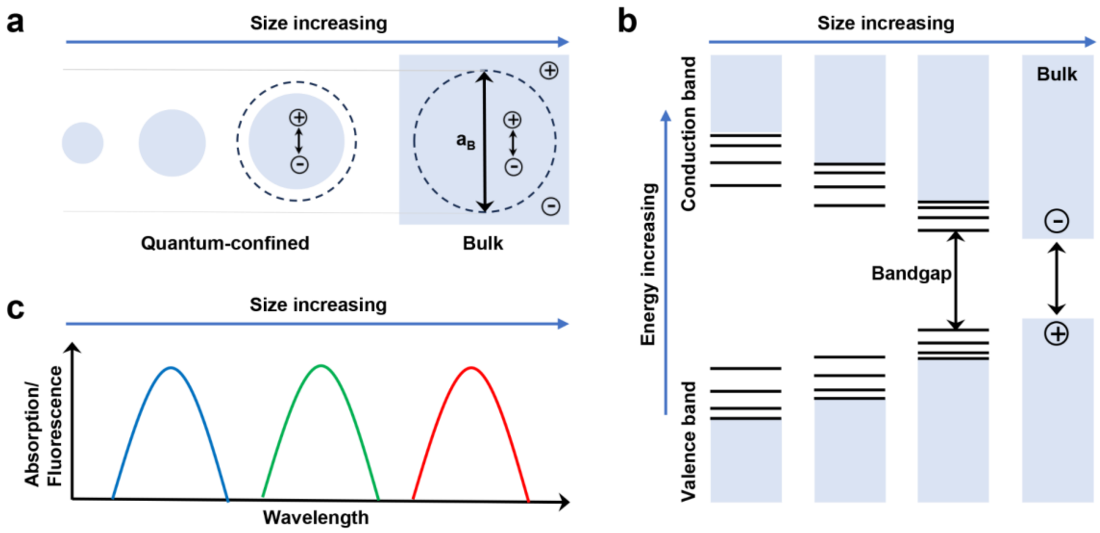

Supplementary Fig. 1 | Illustration of the quantum confinement leading to size-dependent optical and electrical properties.

Quantum confinement, leading to size-dependent optical and electrical properties that are distinct from those of parental bulk solids, occurs when the spatial extent of electronic wave functions is smaller than the Bohr exciton diameter (a_B) (Supplementary Fig. 1a). As QDs become smaller, quantum confinement increases the effective bandgap. Specifically, the energy of the valence band maximum (VBM) will gradually decrease, while the energy of the conduction band minimum (CBM) will gradually increase, leading to a blue shift of the absorption and emission spectra (Supplementary Fig. 1b, c)[24-26]. Specifically, the more negative conduction band potential of CdTe QDs supplies more energetic photoexcited electrons and facilitates the photogenerated electrons transfer to PSII. Meanwhile, the appropriate valence band potential of CdTe QDs also facilitates the recombination of photogenerated holes with electrons from biocompatible sodium L-ascorbate to promote electrons separation. Thus, we chose CdTe QDs for integration with thylakoid.

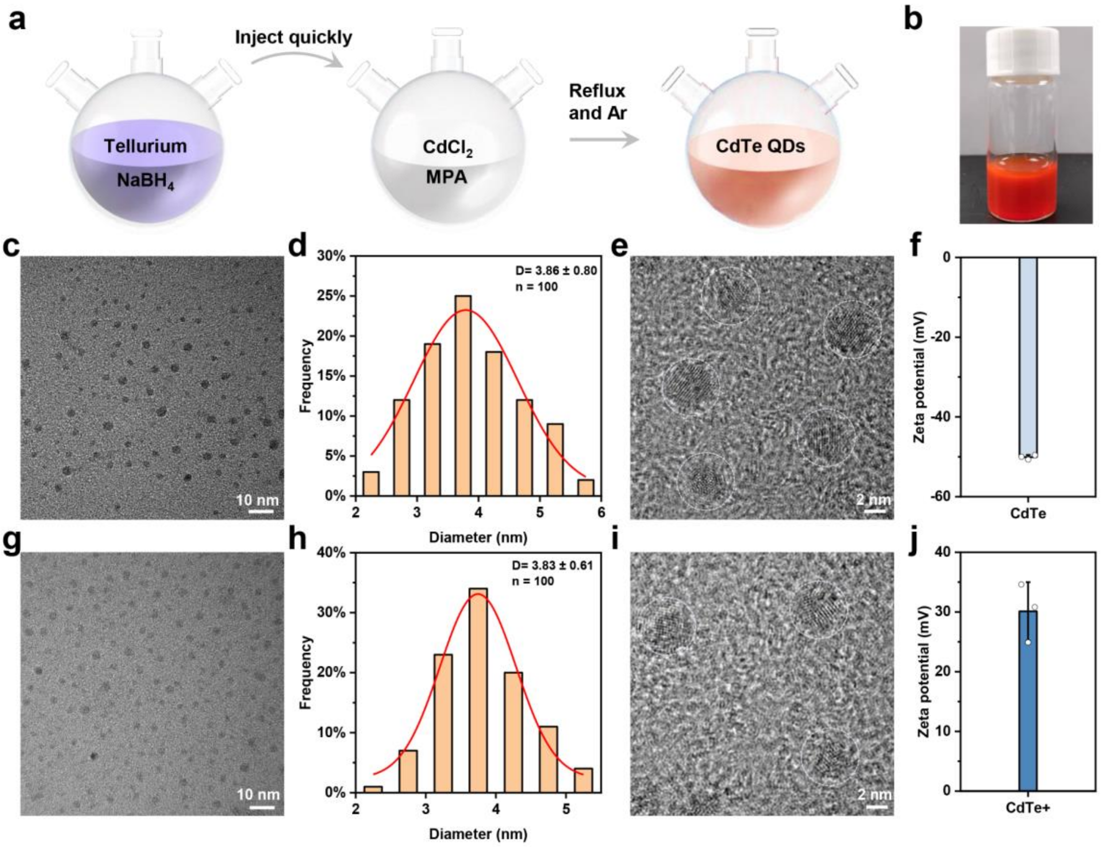

Supplementary Fig. 2 | Characterizations of positive charge CdTe QDs ($CdTe^+$). (**a**) Schematic illustration for the preparation of CdTe QDs. (**b**) Photograph of prepared CdTe QDs dispersion. (**c**) TEM image of CdTe QDs. (**d**) Statistical size distribution of the as-synthesized CdTe QDs. (**e**) HRTEM image of CdTe QDs. (**f**) Zeta-potential profiles of CdTe QDs. Data points are reported as mean ± standard deviation. (n = 3). (**g**) TEM image of $CdTe^+$. (**h**) Statistical size distribution of $CdTe^+$. (**i**) HRTEM image of $CdTe^+$. (**j**) Zeta-potential profiles of $CdTe^+$. Data points are reported as mean ± standard deviation. (n = 3).

The prepared CdTe QDs show a bright red colour with good aqueous dispersibility under ambient light. The high-resolution TEM (HRTEM) images show that the average size of as synthesized CdTe QDs is 3.86 ± 0.8 nm (Supplementary Fig. 2c, d). The observed lattice fringes in HRTEM images give evidence for the crystalline nature of CdTe QDs (Supplementary Fig. 2e). The mean diameter of the crystalline $CdTe^+$ is approximately 3.83 nm.

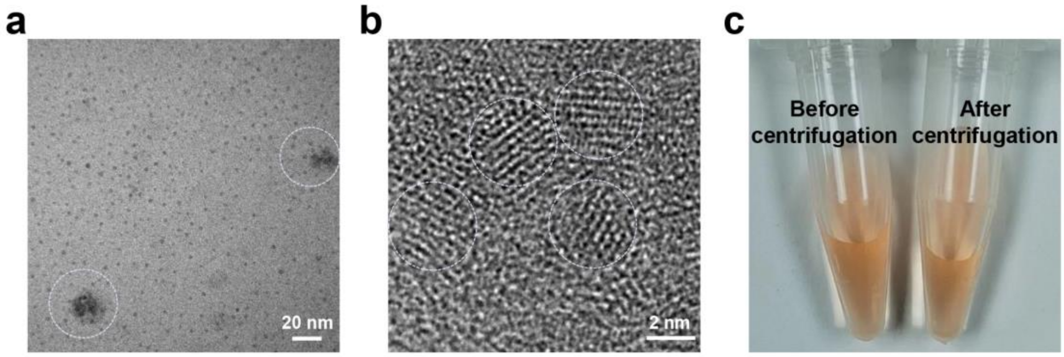

Supplementary Fig. 3 | Characterizations of $CdTe^+$. (**a**) TEM image of $CdTe^+$. The circles outlined by light dashed lines indicate bits of cross-linked PDADMAC and $CdTe^+$. (**b**) HRTEM image of cross-linked PDADMAC and $CdTe^+$. (**c**) Photograph of $CdTe^+$ solution before and after centrifugation at 5,000 × g for 10 min.

Note that the observed bits of aggregates are due to the cross-linked PDADMAC rather than aggregation of $CdTe^+$ (Supplementary Fig. 3a, b). No precipitate could be observed after centrifugation of the $CdTe^+$ solution and it maintains a good dispersion, confirming that there is no aggregated CdTe nanoparticles (Supplementary Fig. 3c).

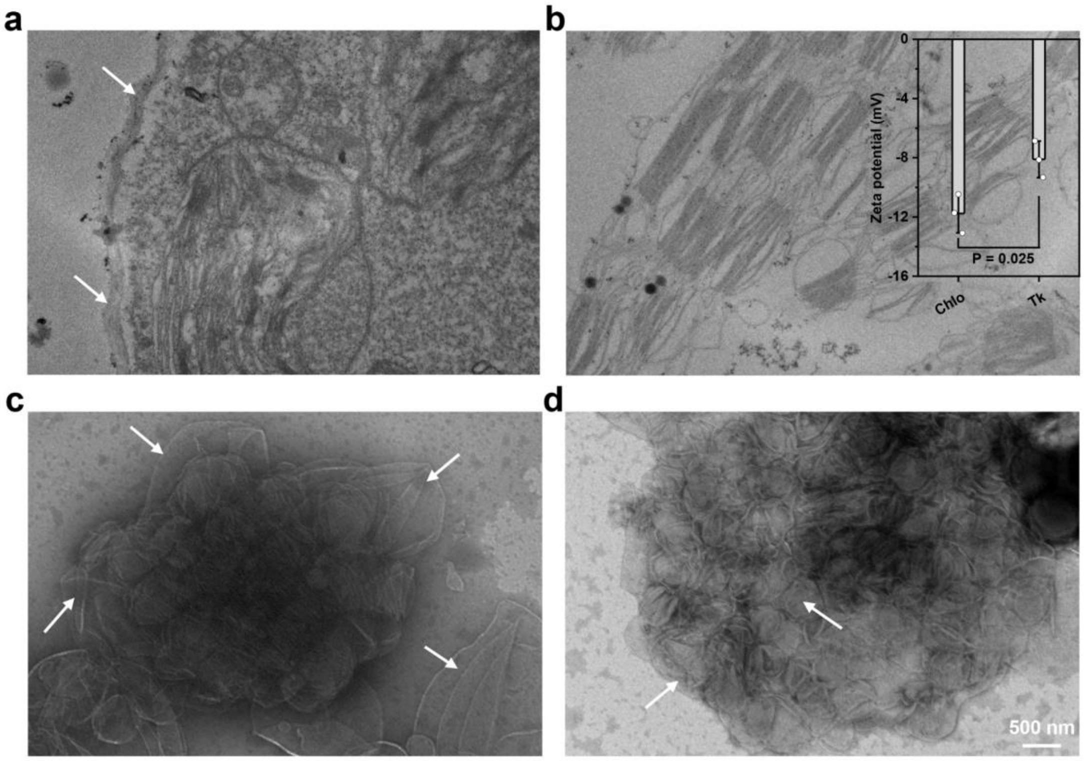

Supplementary Fig. 4 | TEM image of isolated thylakoid. (**a**, **b**) Ultrastructure of (**a**) thylakoid precursor (i.e., chloroplast, abbr., Chlo) and (**b**) thylakoid (abbr., Tk). The white arrows indicate intact plasma membrane. The inset in (**b**) is the comparison of zeta potential between Chlo and Tk. The error bars represent the standard deviations of data from triplicate measurements. $P < 0.05$ (analyzed by two-sided Student's t-test) indicates the significant difference between Chlo and Tk. Data points are reported as mean ± standard deviation. ($n = 3$). (**c**, **d**) TEM images of phosphotungstic acid stained (**c**) Chlo and (**d**) Tk. The white arrows indicate (**c**) broken plasma membrane of Chlo and (**d**) individual spherical stack membrane of thylakoid.

To examine whether thylakoid has been completely isolated from the membrane of chloroplast, we further investigated the location of plasma membrane during the preparation process of thylakoid. Compared with thylakoid, the thylakoid precursor (i.e., chloroplast) shows apparent plasma membrane structure[27], which gives rise to the enrichment of cytoplasmic substance and represents lower zeta potential (Supplementary Fig. 4a, b). Moreover, cell membrane staining[28,29] results reveal that

thylakoid precursor rather than thylakoid has obvious out-membrane-bound structure (Supplementary Fig. 4c), while the isolated thylakoid shows individual spherical stack membrane (Supplementary Fig. 4d). This confirms that thylakoid has been completely isolated from the membrane of chloroplast.

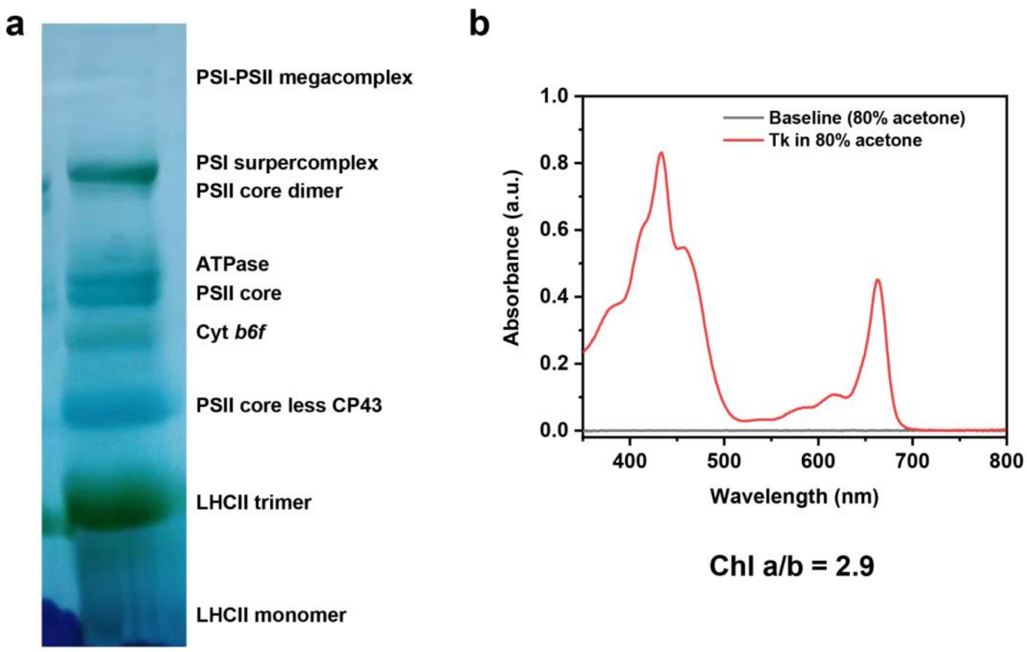

Supplementary Fig. 5 | Characterizations of thylakoid. (**a**) BN-PAGE analysis of photosynthetic apparatus isolated from spinach. The thylakoid was solubilized by 2 % n-Dodecyl-β-D-Maltopyranoside (DDM). Thylakoid loaded in lane was 12.5 μg (equivalent of Chl). PSI: photosystem I; PSII: photosystem II; Cyt *b6f*: cytochrome *b6f* complex; LHCII: light-harvesting complex of photosystem II; CP43: photosystem II chlorophyll binding subunit. (**b**) UV−Vis absorption spectra of Tk in 80% acetone. The Chl a/b ratio in our Tk was calculated to be 2.9.

Tk was prepared by breaking the chloroplast in osmotic shock buffer, which obtained intact thylakoid and plasma membrane of broken chloroplast. Further purification was carried out by throwing away broken membrane with repeated washing. The light-absorbing antennas in Tk are chlorophyll (Chl) a and b. According to the method described by Porra[6,8], the Chl a/b ratio in our thylakoid was calculated to be 2.9 (Supplementary Fig. 5b), which is consistent with the reported literatures[6,8]. Taken together with BN-PAGE analysis (Supplementary Fig. 5a), the results suggest that the intact thylakoid with well-preserved structure and light-harvesting function was obtained in vitro.

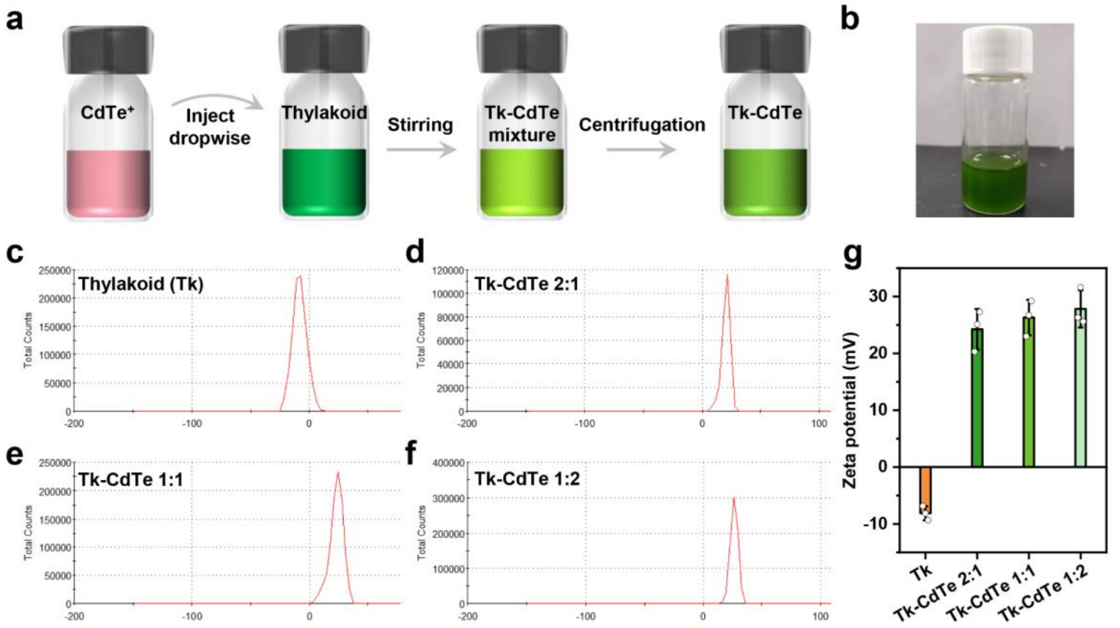

Supplementary Fig. 6 | Characterizations of TK–CdTe. (**a**) Schematic illustration for the preparation of TK–CdTe. (**b**) Photograph of prepared TK–CdTe dispersion. (**c-f**) Zeta-potential profiles of (**c**) TK, (**d**) Tk–CdTe 2:1, (**e**) Tk–CdTe 1:1 and (**f**) Tk–CdTe 1:2. (**g**) Zeta-potential values derived from (**c-f**). Data points are reported as mean ± standard deviation. (n = 3).

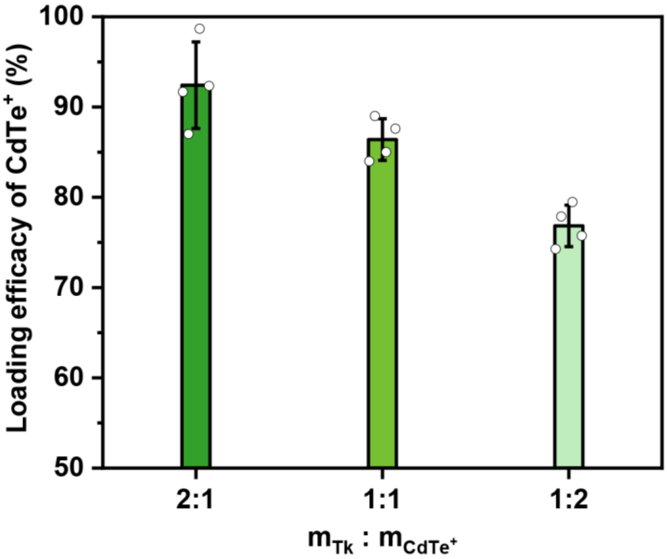

Supplementary Fig. 7 | The ratios of the actual loading amounts of $CdTe^{+}$ in Tk–CdTe (determined by ICP-AES) to the added amounts of $CdTe^{+}$ in the preparation of Tk–CdTe. Data points are reported as mean ± standard deviation. (n = 4).

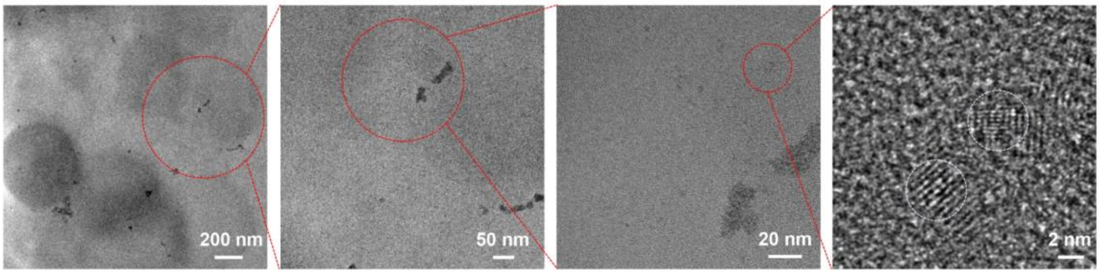

Supplementary Fig. 8 | HRTEM image of $CdTe^+$ located on the thylakoid. The circles outlined by red dashed lines indicate the magnified region.

The thylakoid membrane manly contains lipids and photosynthetic protein complexes, and exhibits negative surface charge mainly attributed to the exposed charged amino acids of the photosynthetic protein[30,31]. The presence of negative charges on thylakoid membrane enables the adsorption of positively charged $CdTe^+$ through electrostatic force, which is confirmed by SEM, EDS mapping, CLSM and TEM images (Fig. 2 and Supplementary Fig. 8). Moreover, the effective adsorption sites should be beneficial for photogenerated electrons transfer, while the pure lipid bilayer has weak capacity for transferring electrons[32]. Therefore, the adsorption sites for $CdTe^+$ on thylakoid membrane could be the negative charged carboxyl in lipids or photosynthetic protein, whereas the effective adsorption sites for $CdTe^+$ on thylakoid membrane are probably the negative charged carboxyl in photosynthetic protein (as demonstrated in Supplementary Fig. 22).

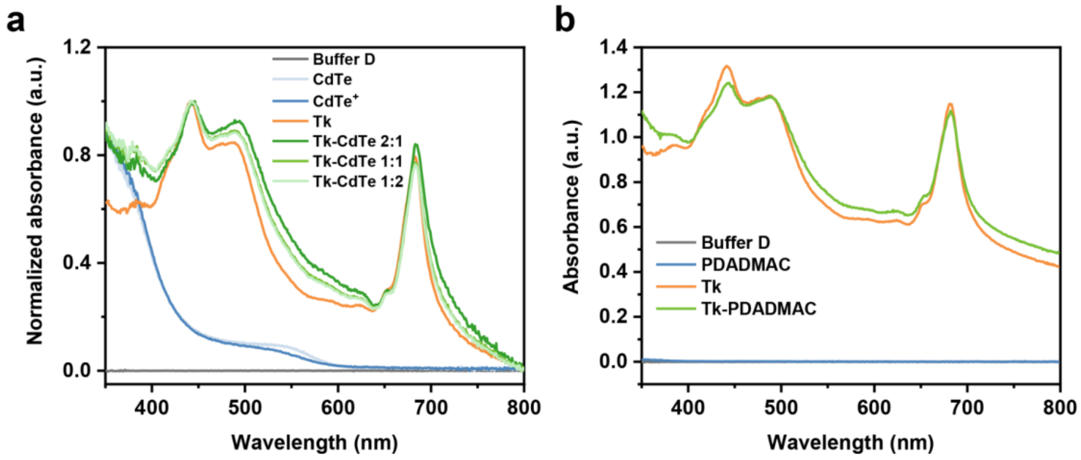

Supplementary Fig. 9 | UV−Vis absorption spectra. (**a**) Normalized UV−Vis absorption spectra of Tk–CdTe in buffer D. The spectra of individual component including CdTe QDs, $CdTe^+$, Tk and buffer D were obtained as the control. (**b**) UV−Vis absorption spectra of Tk–PDADMAC in buffer D. The spectra of individual component including PDADMAC, Tk and buffer D were obtained as the control. The buffer D includes 700 mM sorbitol, 10 mM HEPES-KOH pH 7.6, 10 mM $MgCl_2$, and 10 mM sodium L-ascorbate.

The relative dominant light absorption of Tk is due to the antenna pigments that are not photochemically active. Tk photosynthesis is constrained to the visible range of the solar spectrum, allowing access to only roughly 50% of the incident solar energy radiation and less than 10% of full solar light saturates the capacity of the photosynthetic apparatus[33,34]. In fact, the intrinsic solar light absorption is ascribed to chlorophyll a and b, which exhibits weak capability to capture solar energy (Supplementary Fig. 5b). Thus, Tk generally suffers from low utilization of sunlight, which not only limits the efficiency of the whole photosynthetic process, but also poses a challenge for increasing the supply of photogenerated electrons[35]. In this regard, we envisaged that efficient regeneration of cofactors can be achieved by rationally combining thylakoid and light-harvesting inorganic nanomaterials to increase the supply of photogenerated electrons.

PDADMAC itself does not show any absorption at the wavelength in the range of 350 to 800 nm. Interestingly, $CdTe^+$ indicated slightly decreased absorption at the wavelength of ~540 nm after the PDADMAC modification (Supplementary Fig. 9a). This is probably due to the decreased absolute value of zeta potential for CdTe QDs after surface modification of PDADMAC (from -50.1 mV to 30.1 mV, Supplementary Fig. 2), which leads to weaker repulsive force, shortened space distance of the colloid and thus the changed light absorption peak.

After the incorporation of CdTe QDs on thylakoid, the Tk−CdTe shows enhanced light absorption in the range of 700 to 800 nm (Supplementary Fig. 9a), despite there was no contributed absorption of CdTe QDs in that range. Similar phenomenon was observed when pure PDADMAC was absorbed on thylakoid (Supplementary Fig. 9b), indicating that the slightly promoted light-harvesting capability is probably ascribed to the introduced PDADMAC.

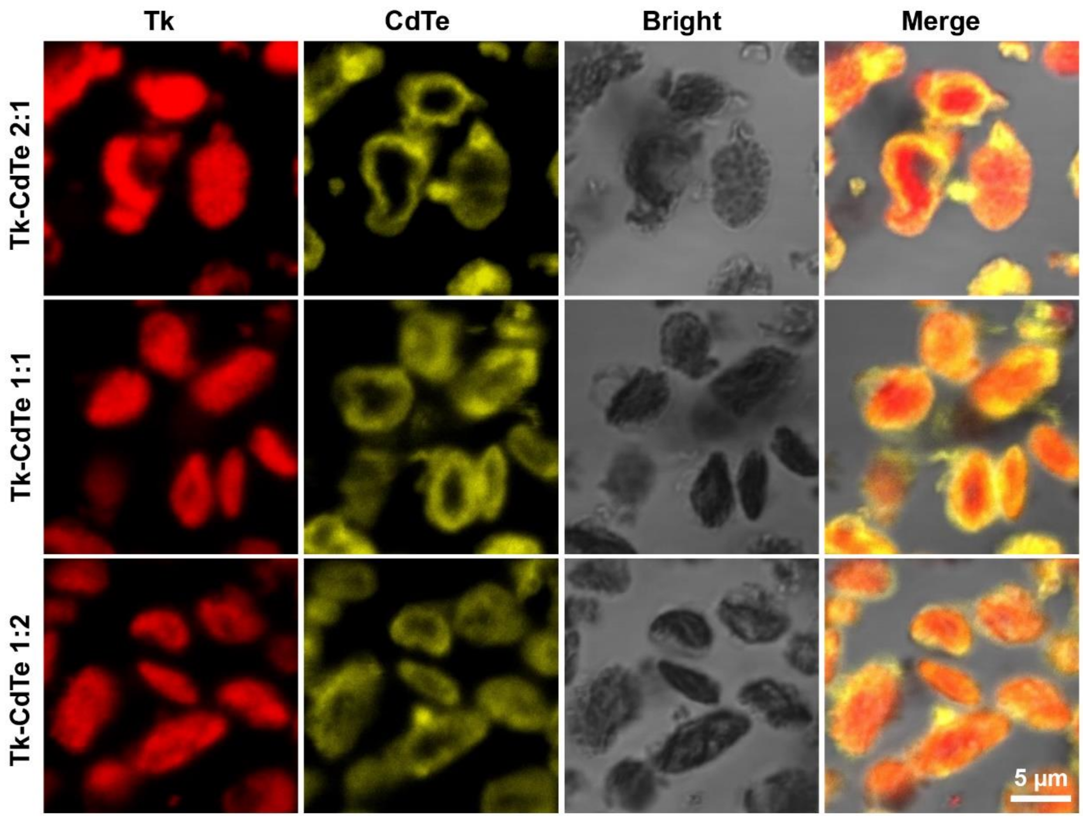

Supplementary Fig. 10 | Enlarged CLSM images of Tk–CdTe 2:1 (1st row), Tk–CdTe 1:1 (2nd row) and Tk–CdTe 1:2 (3rd row). Tk and $CdTe^{+}$ are indicated by red and yellow fluorescence signals, respectively.

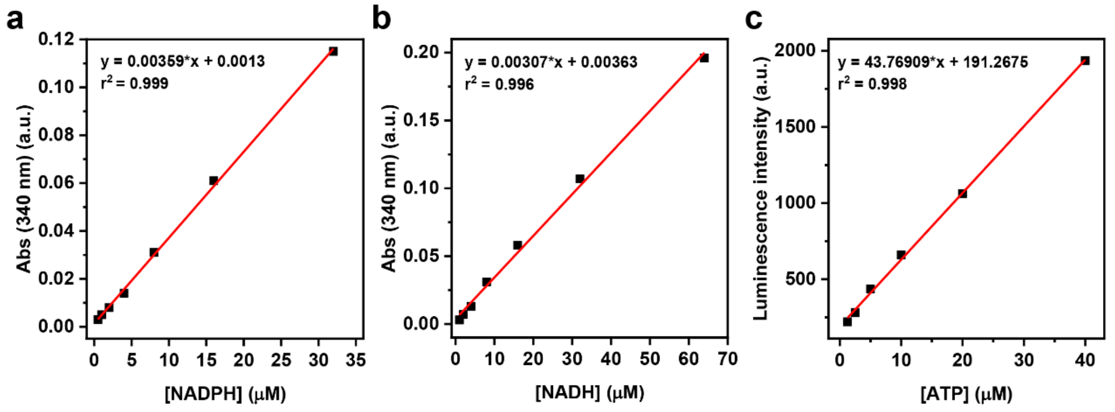

Supplementary Fig. 11 | Standard curves for the detection and quantification of regenerated cofactors. The relationship of (**a**) NADPH and (**b**) NADH absorbance at 340 nm *versus* their concentrations. (**c**) The relationship of ATP assay luminescence at 555 nm *versus* its concentration.

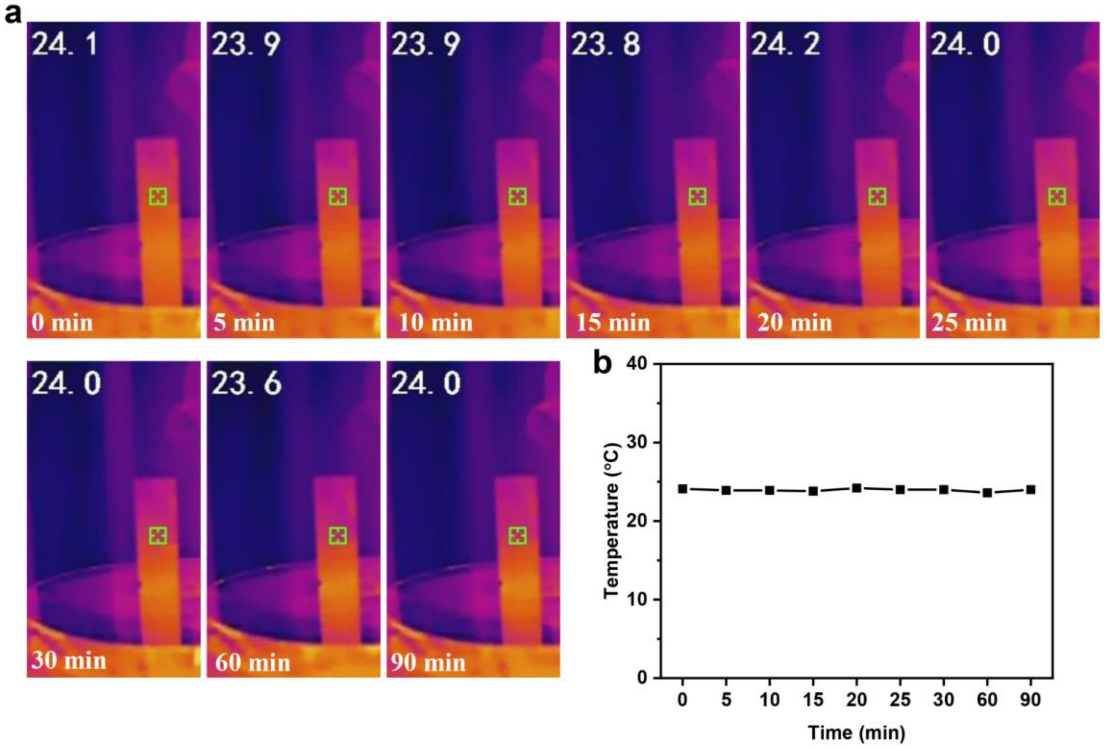

Supplementary Fig. 12 | Temperature monitoring of the reaction system along with light illumination time. (**a**) The temperature monitored by using thermal imaging camera at various light illumination time under a light intensity of 0.1 W/cm^2. (**b**) Data statistics of monitored temperature with various illumination time.

The temperature of the reaction system under the light intensity of 0.1 W/cm^2 was as low as the ambient temperature and it has no obvious change throughout the reaction, suggesting that there is no significant photothermal effect on NADPH regeneration by thylakoid under the light intensity of 0.1 W/cm^2.

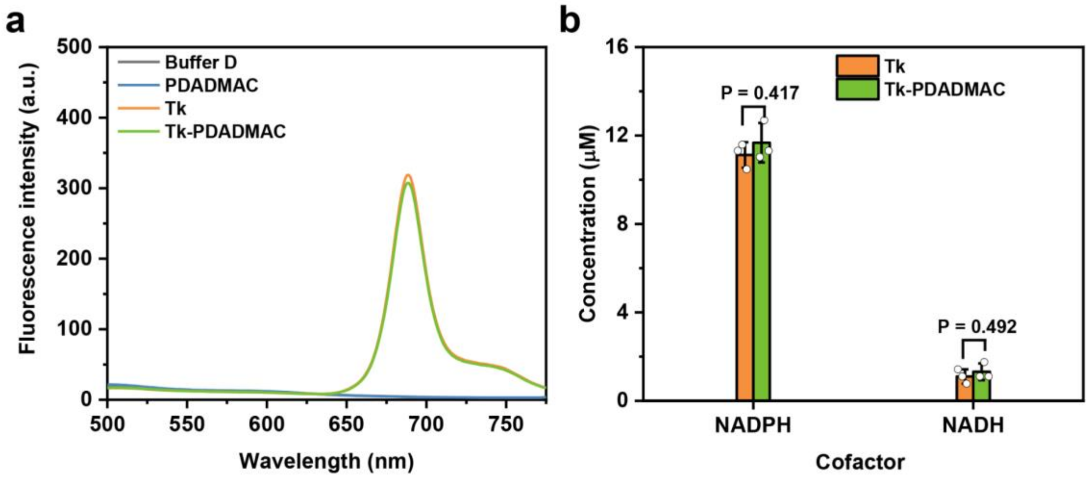

Supplementary Fig. 13 | The contribution of PDADMAC to cofactors regeneration. (**a**) Fluorescence spectra of Tk–PDADMAC in buffer D. The spectra of PDADMAC, Tk and buffer D were collected as the control. (**b**) NADPH and NADH regeneration by Tk–PDADMAC or Tk with 10 μg/mL Chl equivalent. Data points are reported as mean ± standard deviation (n = 3). P > 0.05 (analyzed by two-sided Student's t-test) indicates no significant difference between Tk–PDADMAC and Tk for cofactors regeneration.

Although the modification of PDADMAC can lead to a slightly promoted absorption for Tk-PDADMAC at the wavelength of 700-800 nm (Supplementary Fig. 9), no fluorescence signal of PDADMAC can be detected while PDADMAC has negligible influence on the fluorescence emission of Tk.

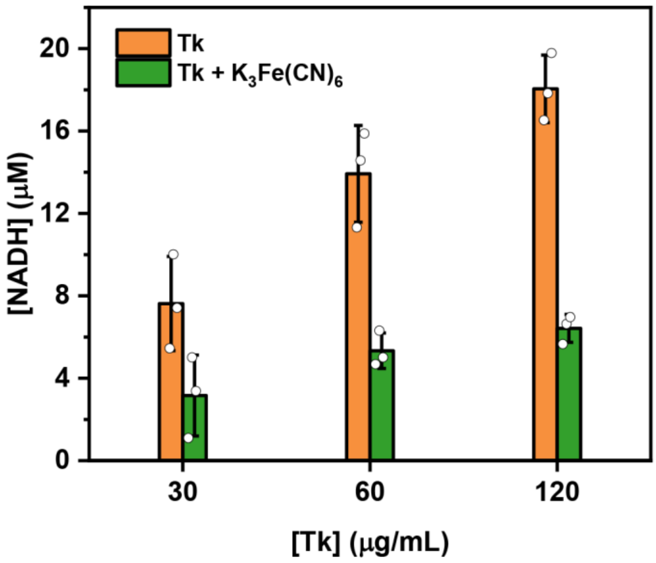

Supplementary Fig. 14 | NADH regeneration by Tk with 0.1 mM $K_3Fe(CN)_6$ treatment. Data points are reported as mean ± standard deviation (n = 3).

We further verified that the ferredoxin-$NADP^+$ reductase (FNR) in Tk catalyzes the reduction of NAD^+ to NADH by evaluating the NADH regeneration capability in the presence of ferricyanide. It has reported that ferricyanide (as an electron acceptor) is photo-reduced primarily by FNR-contained photosystem I (PSI)[36,37]. Our experiment results show that the NADH regeneration capability by Tk is significantly reduced when the ferricyanide is introduced as a competitive electron acceptor (Supplementary Fig. 14), indicating that the reduction of NAD^+ to NADH occurs at the same FNR in PSI that produces NADPH.

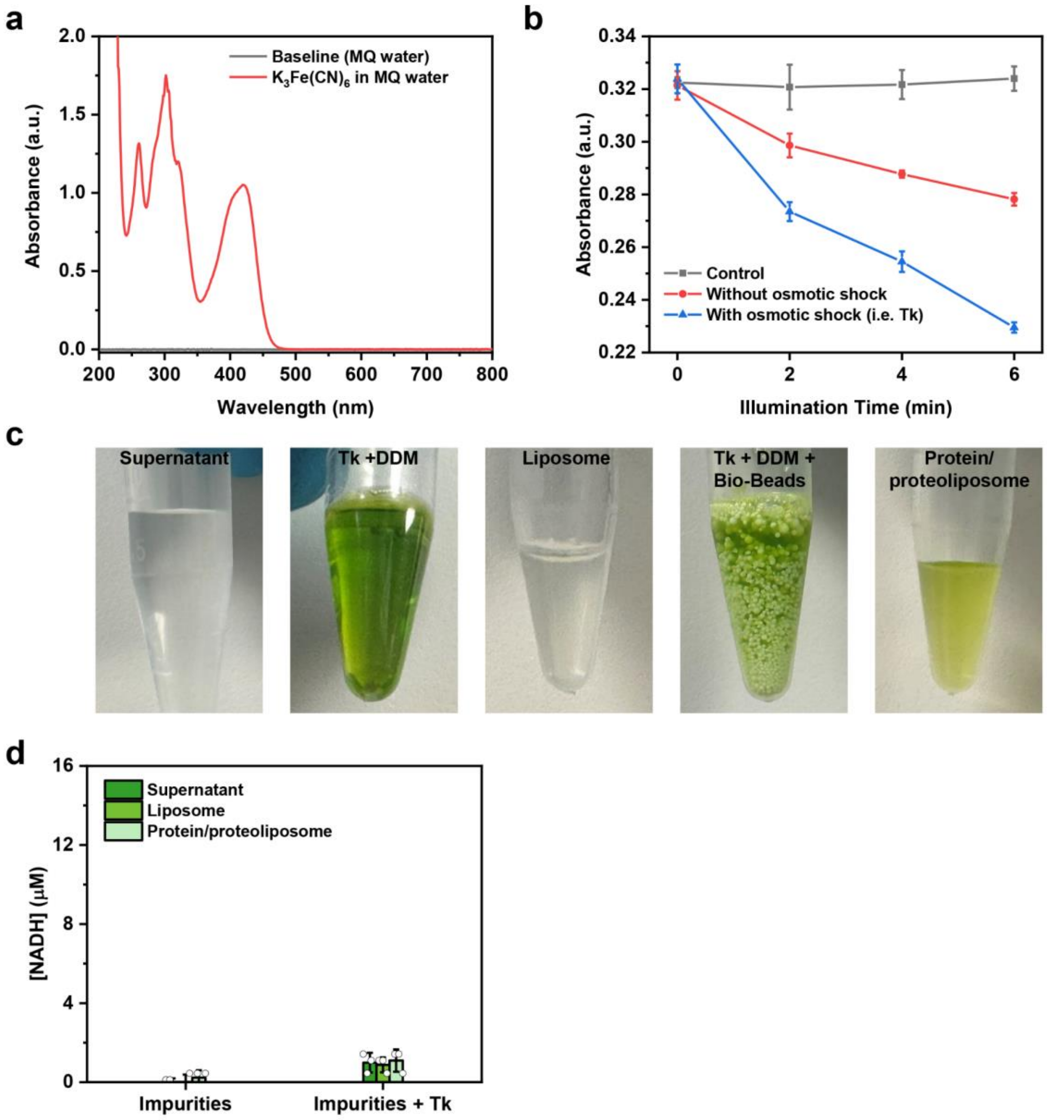

Supplementary Fig. 15 | The isolation of possible impurities and its influence on NADH regeneration by Tk. (**a**) UV−vis absorption spectra of $K_3Fe(CN)_6$ in Millipore water. (**b**) $K_3Fe(CN)_6$ photoreduction assay over Tk with/without osmotic shock buffer treatment. Note: the presence of intact plasma membrane in Tk without osmotic shock buffer treatment hinders the photoreduction of $K_3Fe(CN)_6$. Data points are reported as mean ± standard deviation (n = 3). (**c**) Possible impurities in the process of Tk isolation, containing broken plasma membrane, liposome and protein/proteoliposome. Supernatant: broken plasma membrane-contained buffer. Tk + DDM: isolated Tk solubilized by 2 % DDM. Liposome: precipitate via centrifugation from solubilized Tk.

Tk + DDM + Bio-Beads: Bio-Beads added into solubilized Tk mixture. Protein/proteoliposome: reconstituted protein via removing DDM. (**d**) NADH regeneration by Tk with impurities (plasma membrane, liposome and protein/proteoliposome) treatment. Data points are reported as mean ± standard deviation (n = 3).

Then, we further performed some control experiments to eliminate the influence of possible impurities (e.g., plasma membrane, broken liposome and protein/proteoliposome) on NADH regeneration. The presence of plasma membrane in the supernatant of broken chloroplast buffer was verified by ferricyanide photoreduction assay (https://www.sigmaaldrich.cn/deepweb/assets/sigmaaldrich/product/documents/638/446/cpisopis-mk.pdf). As shows in Supplementary Fig. 15a, b, ferricyanide was partially photo-reduced due to the interference of intact plasma membrane without osmotic shock buffer treatment, confirming the presence of plasma membrane. We obtained the plasma membrane by collecting the supernatant in broken chloroplast with osmotic shock buffer treatment (Supplementary Fig. 15c) to estimate its influence on NADH regeneration. Similarly, we collected the broken liposome and protein/proteoliposome from thylakoid via n-Dodecyl-β-D-Maltopyranoside (DDM)-mediated method[10,11] to estimate their influence on NADH regeneration (Supplementary Fig. 15c). The results demonstrate that the possible impurities have negligible NADH regeneration capacity and also have no significant influence on NADH regeneration by Tk (Supplementary Fig. 15d).

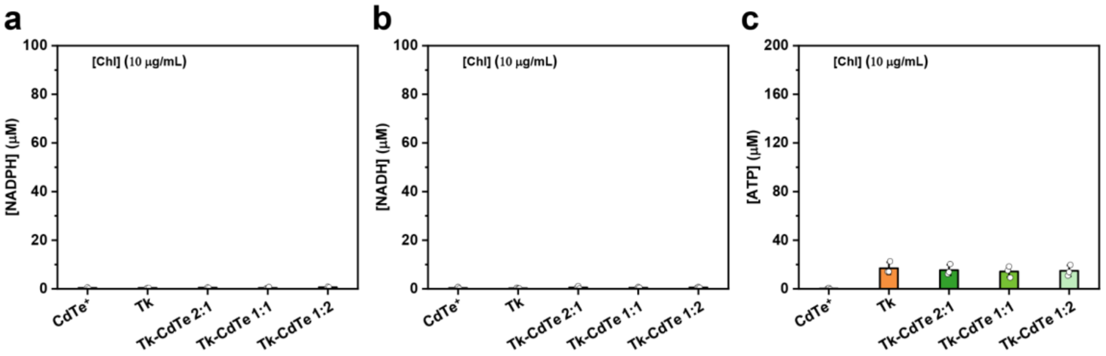

Supplementary Fig. 16 | NADPH, NADH and ATP regeneration without light illumination. (**a**) NADPH, (**b**) NADH and (**c**) ATP regeneration by $CdTe^+$, Tk or Tk–CdTe with 10 μg/mL Chl equivalent or equal amount $CdTe^+$ (i.e., Chl equivalent × loading efficacy; similarly treated hereinafter). Data points are reported as mean ± standard deviation (n = 3).

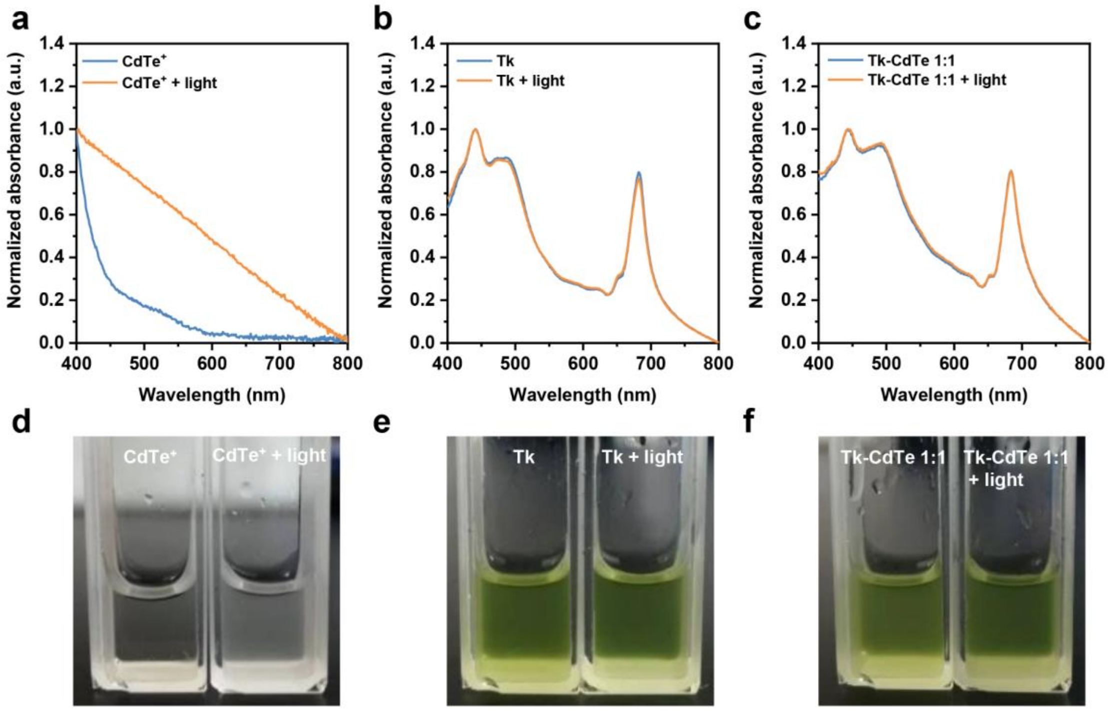

Supplementary Fig. 17 | Photostability of Tk–CdTe after 30 min light illumination. **(a–c)** Normalized UV-Vis absorption spectra of (**a**) $CdTe^+$, (**b**) Tk and (**c**) Tk–CdTe 1:1 in NADPH regeneration buffer before and after illumination. (**d–f**) Photographs of (**d**) $CdTe^+$, (**e**) Tk and (**f**) Tk-CdTe 1:1 dispersion solution before and after illumination under the light intensity of 0.1 W/cm^2.

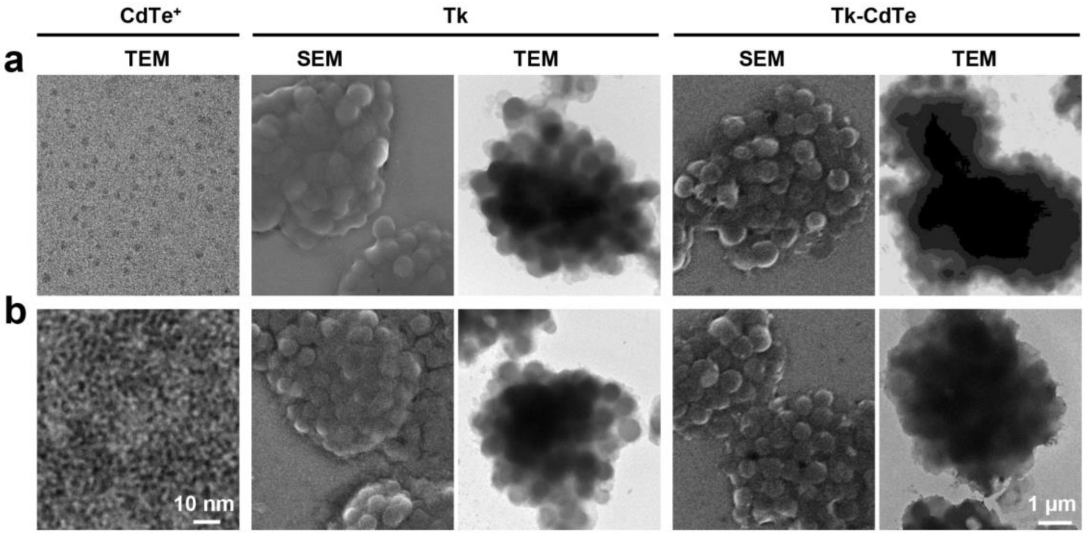

Supplementary Fig. 18 | TEM and SEM images of $CdTe^{+}$, Tk and Tk–CdTe 1:1.

(**a**) Before and (**b**) after 30 min illumination under the light intensity of 0.1 W/cm^2.

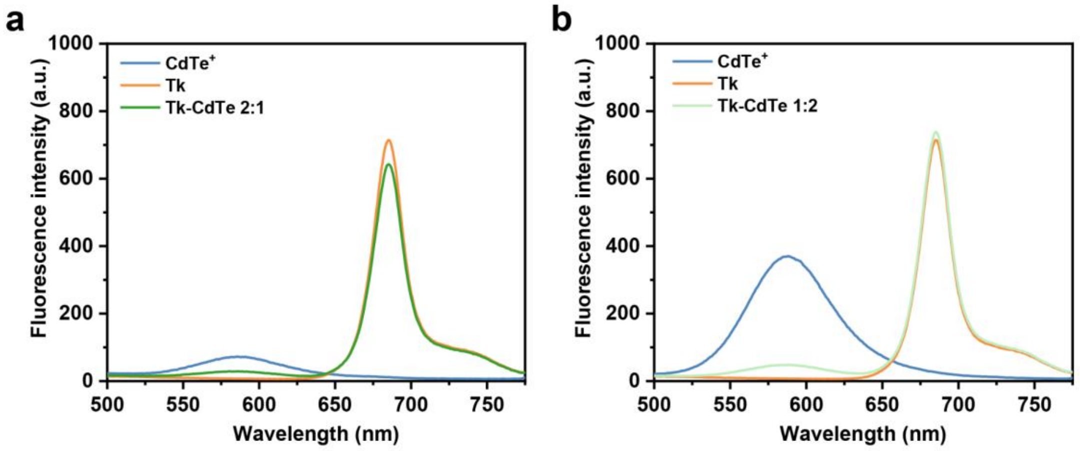

Supplementary Fig. 19 | Tk−CdTe with promoted electron transfer performance. Fluorescence spectra of (**a**) Tk−CdTe 2:1 and (**b**) Tk−CdTe 1:2 with 20 μg/mL Chl equivalent in buffer D with an excitation wavelength of 400 nm, in reference to $CdTe^{+}$ and Tk.

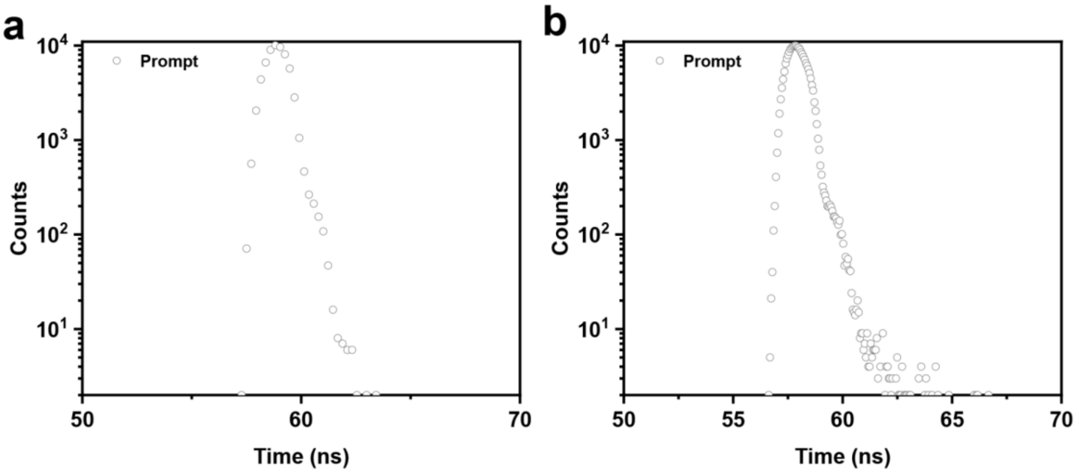

Supplementary Fig. 20 | Instrument response function (IRF) (i.e., prompt spectrum). The IRF with (**a**) NanoLED-370 and (**b**) NanoLED-450 as the excitation source.

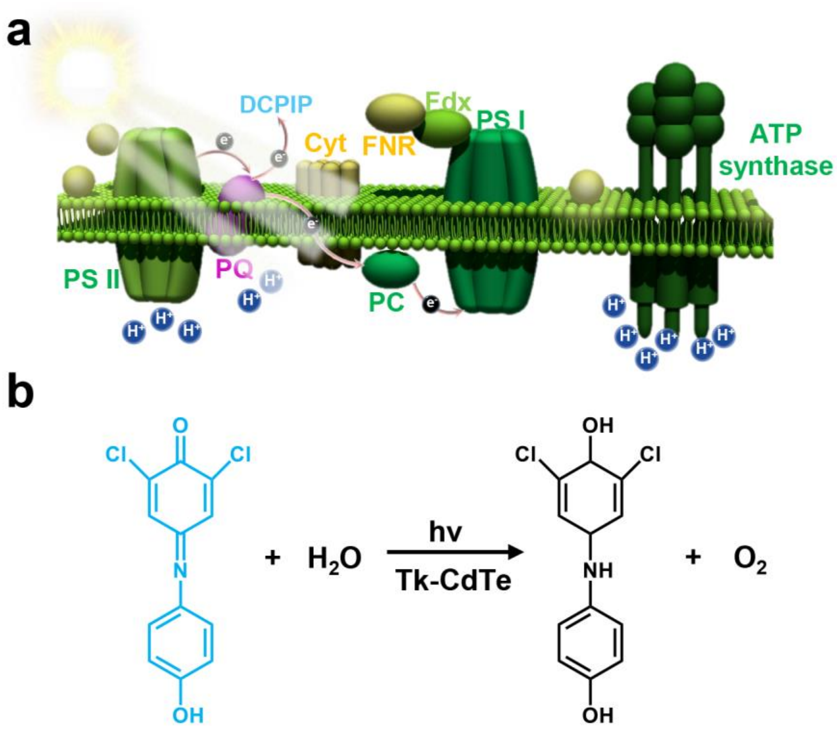

Supplementary Fig. 21 | Working principle of DCPIP. (**a**) DCPIP as an artificial electron acceptor, which can capture electrons transported from PSII to PSI in the photoreaction. (**b**) Schematic illustration for the reduction of DCPIP under light illumination. DCPIP alone indicates strong absorption at 595 nm. With electron capture, the light absorption of DCPIP will decrease.

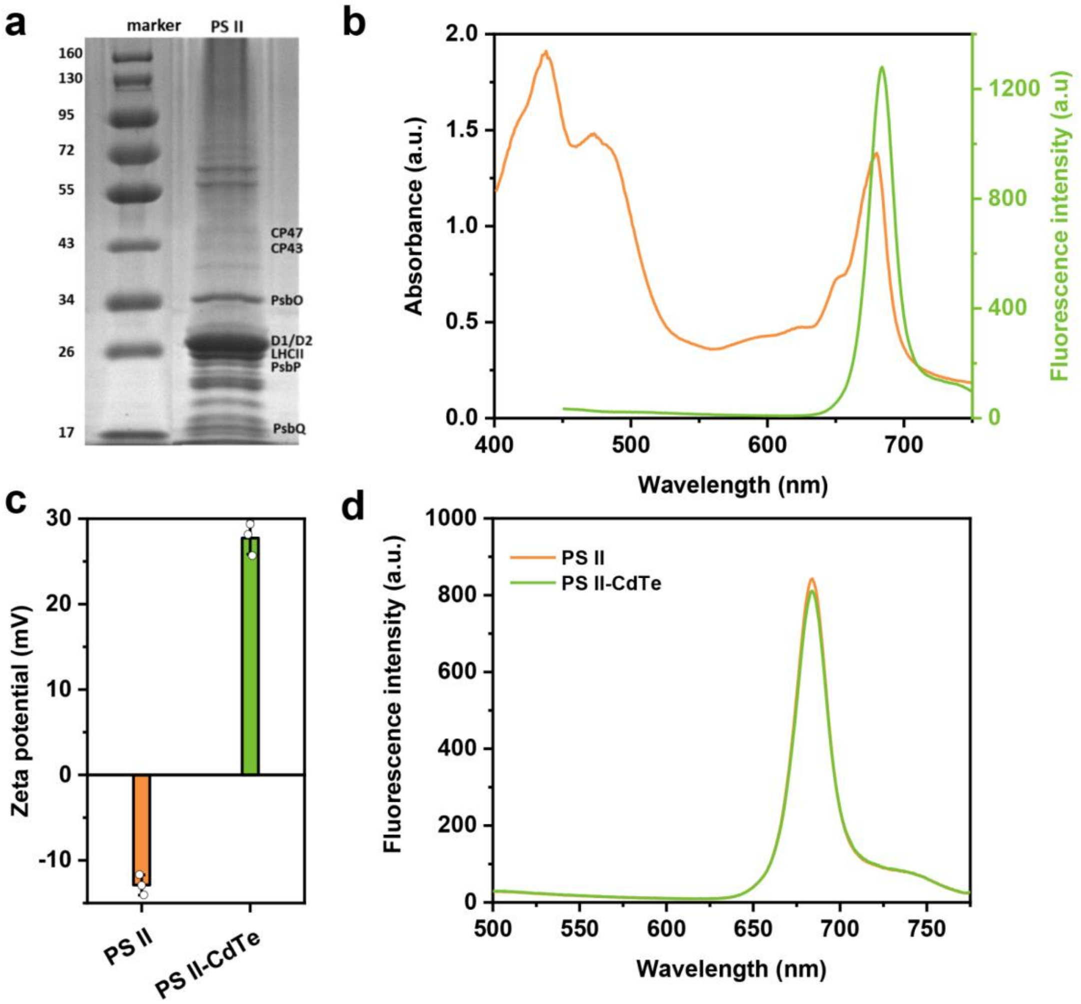

Supplementary Fig. 22 | Characterization of PSII-CdTe. (**a**) SDS–PAGE analysis of PSII. (**b**) Absorption and fluorescence spectra of PSII in buffer E1. The excitation wavelength is 400 nm. (**c**) Zeta-potential and (**d**) fluorescence spectra of PSII and PSII-CdTe in buffer E1. Data points are reported as mean ± standard deviation (n = 3).

We have demonstrated the promoted electrons transfer for PSII-CdTe (Fig. 4h). Considering that PSII is the initial site of electrons transfer, accepting photogenerated electrons from $CdTe^+$ (Fig. 4i), we thus reasonably propose that the negative charged carboxyl of protein in PSII (LHCII complex and PSII core complex, Supplementary Fig. 22a) is likely the effective adsorption sites for $CdTe^+$. However, currently it is difficult to determine exactly the particular protein sites for $CdTe^+$ due to the complex structure of proteins in PSII, thus requiring further exploration in future.

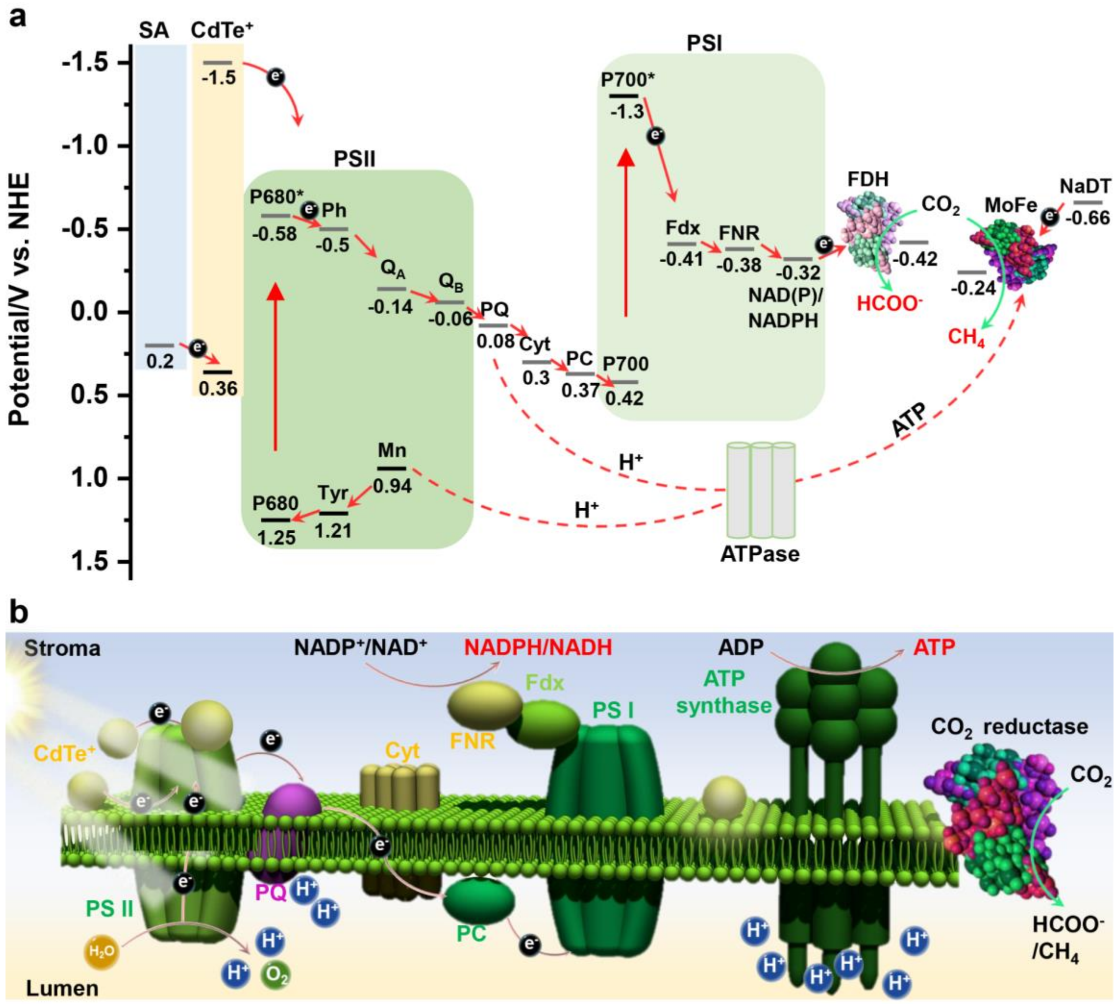

Supplementary Fig. 23 | Cofactors regeneration and CO_2 reduction. (**a**) NAD(P)H and ATP regeneration via electron transfer chain and their redox potential levels to the CO_2 conversion. (**b**) Schematic illustration of a possible mechanism for electron transfer from excited $CdTe^{+}$ to photosystem. Upon light illumination, the energy modules regenerate bioactive NADPH, NADH and ATP cofactors, which can be coupled with various cofactors-dependent CO_2 reductases to drive CO_2 conversion. SA: sodium L-ascorbate. Mn, Tyr, P680, Ph, Q_A, Q_B, PQ, Cyt, PC and P700 are the abbreviation of the electron transfer factors in photosystem. Fdx: Ferredoxin. FNR: Ferredoxin-$NADP^{+}$ oxidoreductase. NaDT: sodium dithionite. FDH: formate dehydrogenases. MoFe: remodeled nitrogenase.

The electrons transfer process in PSII component has been illustrated in Fig. 4i and

Supplementary Fig. 23a[38-40]. Specifically, the photogenerated electrons from CdTe QDs transfer into the PSII through electron transport chain. Meanwhile, a photon inputs energy into the PSII by stimulating P680, which releases energetic electrons and produces the highly reactive P680* radical. The electron moves to pheophytin, and subsequently to electron transfer cofactors (Q_A and Q_B). P680 donates electrons and transforms into strong biological oxidizing agent (or electron acceptor), which captures the electron from a tyrosine residue (Tyr). The oxidized Tyr receives the electron from Mn via the splitting of water (H^+ and O_2 generation). This electron flow continues from photoexcited CdTe QDs and water oxidation to the PSII as long as Tk-CdTe is stimulated by photons. The interface between CdTe QDs and PSII plays a key role in electron transport, which increases the supply of photogenerated electrons to boost the cofactors regeneration by Tk. The rational integration of thylakoid with CdTe QDs substantially enhances the regeneration of bioactive NADPH, NADH and ATP cofactors without external supplements by promoting proton-coupled electron transfer. The photogenerated hole by P680 is likely finally consumed on Mn for oxidization of water (H^+ and O_2 generation) in PSII. The oxidation potential of sodium L-ascorbate is 0.2 V vs. NHE[41] which is suitable to recombine with the photogenerated hole of CdTe QDs.

Overall, CdTe QDs and PSII transfer photogenerated electrons to PSI via electron transport chain, and the photogenerated electrons ultimately are accepted by $NAD(P)^+$ to produce NAD(P)H (Supplementary Fig. 23b). During the electrons transfer process, a proton gradient, which is derived from the electron transport chain (i.e., oxidizing of reduced plastoquinone) or splitting of water, is generated across the thylakoid membrane, driving the synthesis of ATP. Finally, the produced cofactors of NADPH, NADH and ATP are used to reduce CO_2 via CO_2 reductase. NADPH or NADH as reducing equivalents are utilized by FDH to convert CO_2 into $HCOO^-$. ATP is utilized by MoFe to power the conversion of CO_2 into CH_4 through combining with NaDT, which has an oxidation potential of -0.66 V vs. NHE[42] and is typically used as an electron donor for MoFe protein catalysis[43,44].

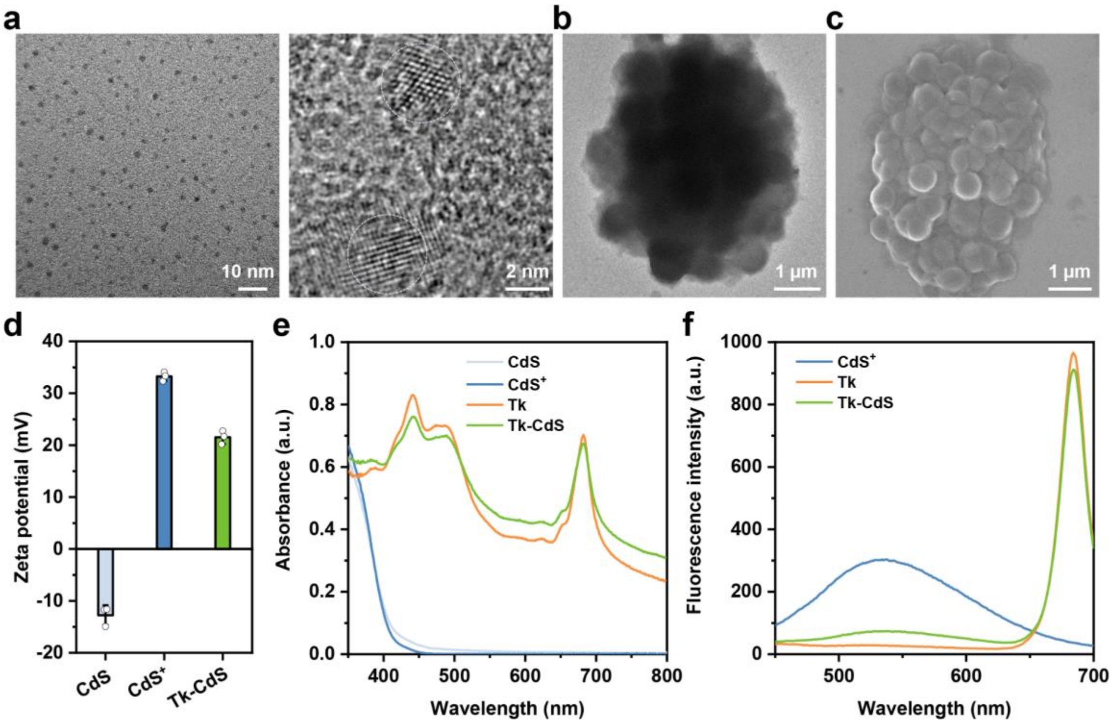

Supplementary Fig. 24 | Characterization of Tk–CdS. (**a**) TEM and HRTEM images of CdS QDs. (**b**) TEM and (**c**) SEM image of Tk–CdTe. (**d**) Zeta-potential analysis of CdS QDs, CdS^+ and Tk–CdS. The error bars represent the standard deviations of data from triplicate measurements. Data points are reported as mean ± standard deviation (n = 3). (**e**) UV-Vis absorption spectra of Tk–CdS in buffer D. The spectra of individual component including CdS QDs, CdS^+ and Tk were obtained as the control. (**f**) Fluorescence spectra of Tk–CdS with 20 μg/mL Chl equivalent in buffer D, with an excitation wavelength of 370 nm. The spectra of individual component including CdS^+ and Tk were obtained as the control.

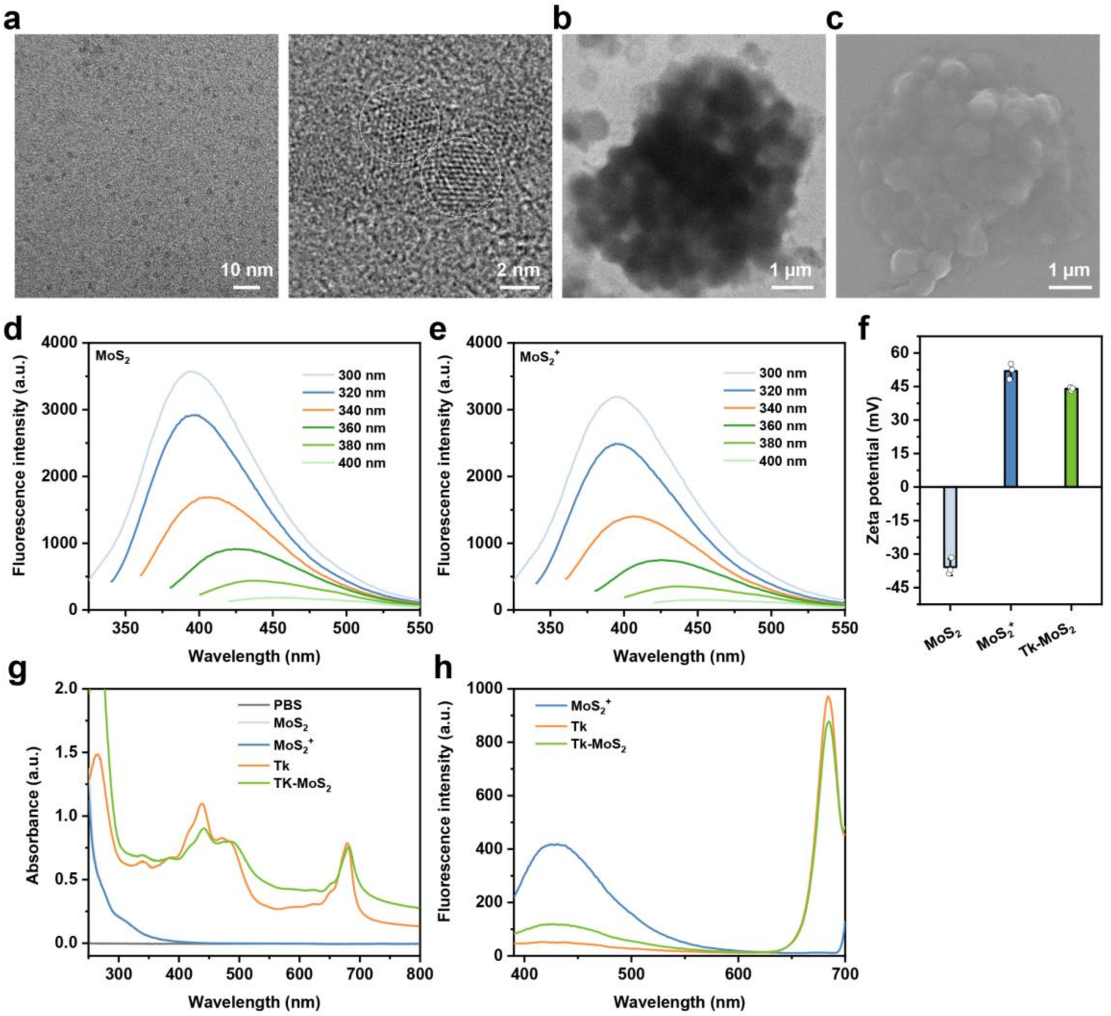

Supplementary Fig. 25 | Characterization of Tk–MoS_2. (**a**) TEM and HRTEM images of MoS_2 QDs. (**b**) TEM and (**c**) SEM image of Tk–MoS_2. (**d, e**) Fluorescence spectra of (**d**) MoS_2 QDs and (**e**) MoS_2^+ excited at different wavelengths. (**f**) Zeta-potential analysis of MoS_2 QDs, MoS_2^+ and Tk–MoS_2. The error bars represent the standard deviations of data from triplicate measurements. Data points are reported as mean ± standard deviation (n = 3). (**g**) UV-Vis absorption spectra of Tk–MoS_2 in PBS. The spectra of individual component including MoS_2 QDs, MoS_2^+, Tk and PBS were obtained as the control. (**h**) Fluorescence spectra of Tk–MoS_2 with 20 μg/mL Chl equivalent in PBS, with an excitation wavelength of 360 nm. The spectra of individual component including MoS^+ and Tk were obtained as the control.

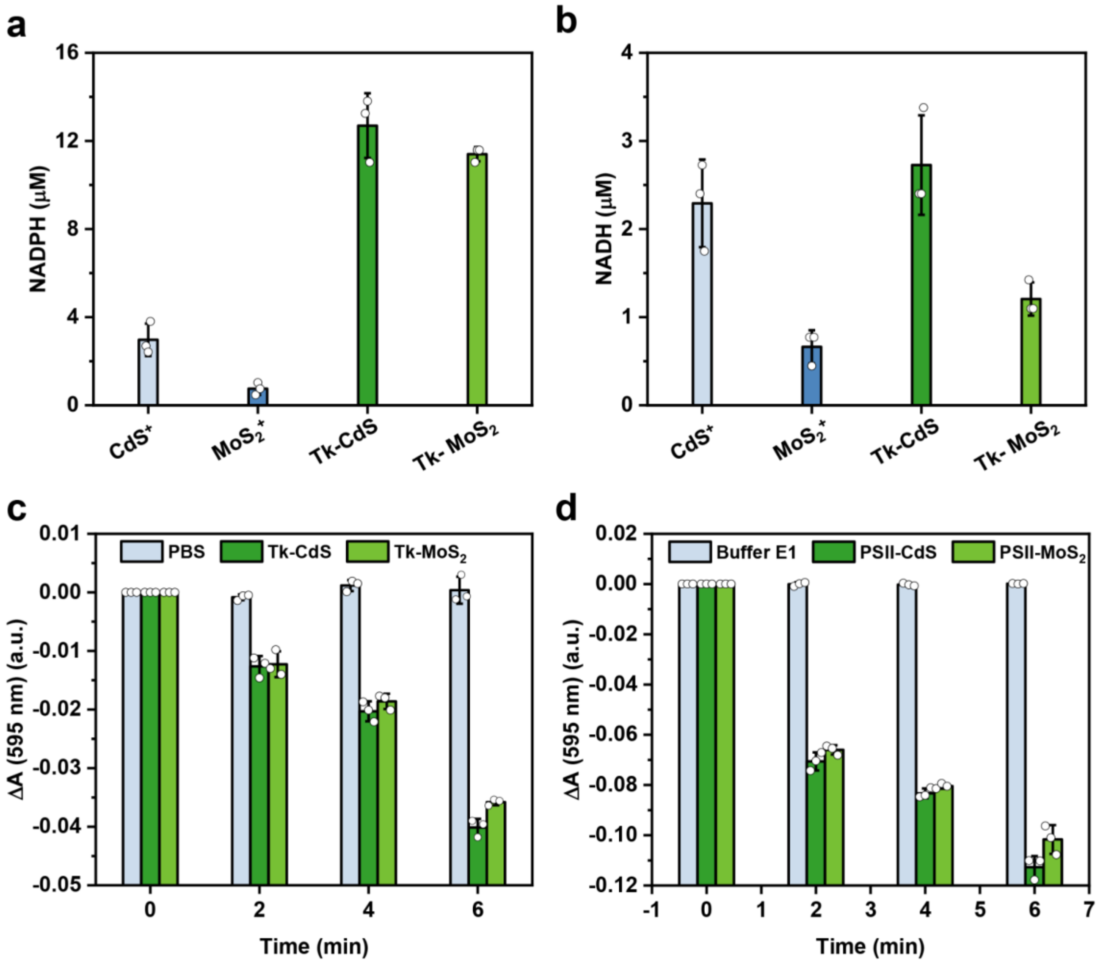

Supplementary Fig. 26 | Light-driven regeneration of NADPH and NADH by Tk–CdS or Tk–MoS_2. (**a**) NADPH and (**b**) NADH regeneration by Tk–CdS or Tk–MoS_2 with 10 μg/mL Chl equivalent. CdS^+ and MoS_2^+ were treated similarly as control. Data points are reported as mean ± standard deviation (n = 3).

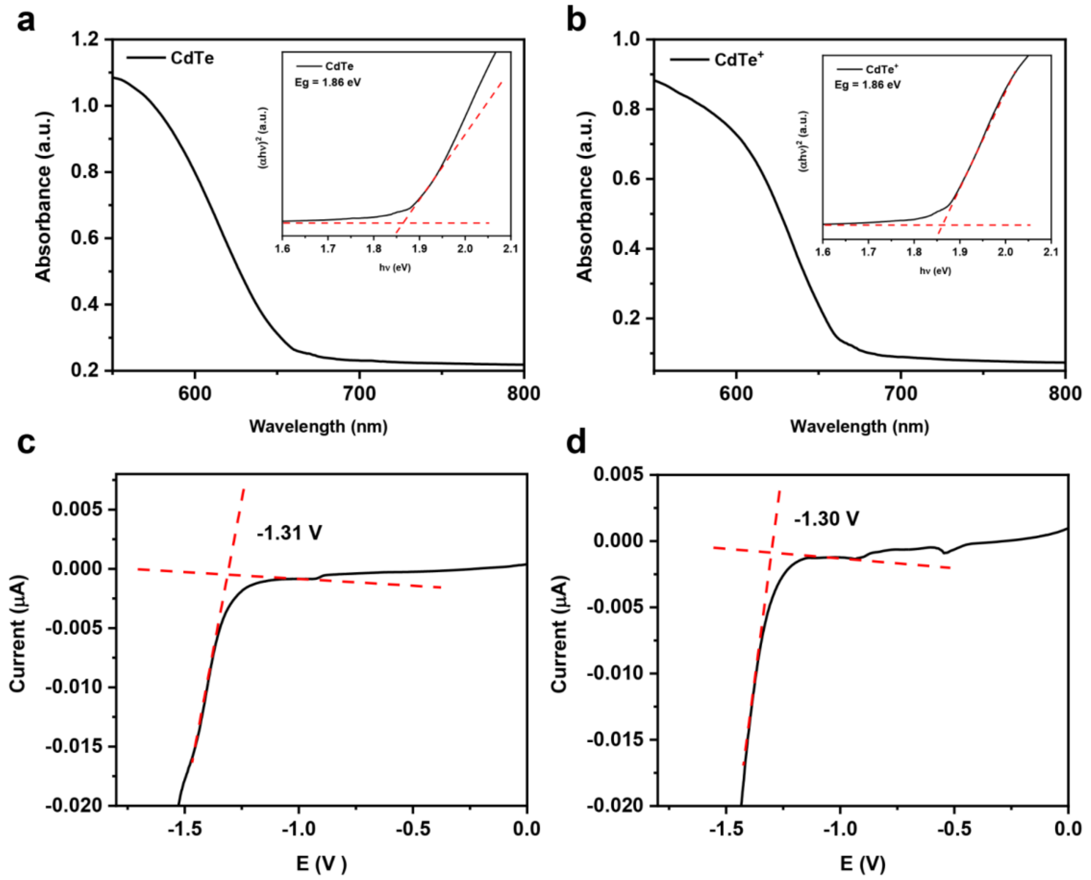

Supplementary Fig. 27 | Calculation of energy band diagrams of CdTe QDs. (**a, b**) UV−Vis diffuse reflectance spectra and (inset) calculated band gap of (**a**) CdTe QDs and (**b**) $CdTe^+$. Optical band gap was determined with a Tauc plot. Linear sweep voltammetry (LSV) curves of (**c**) CdS QDs and (**d**) $CdTe^+$.

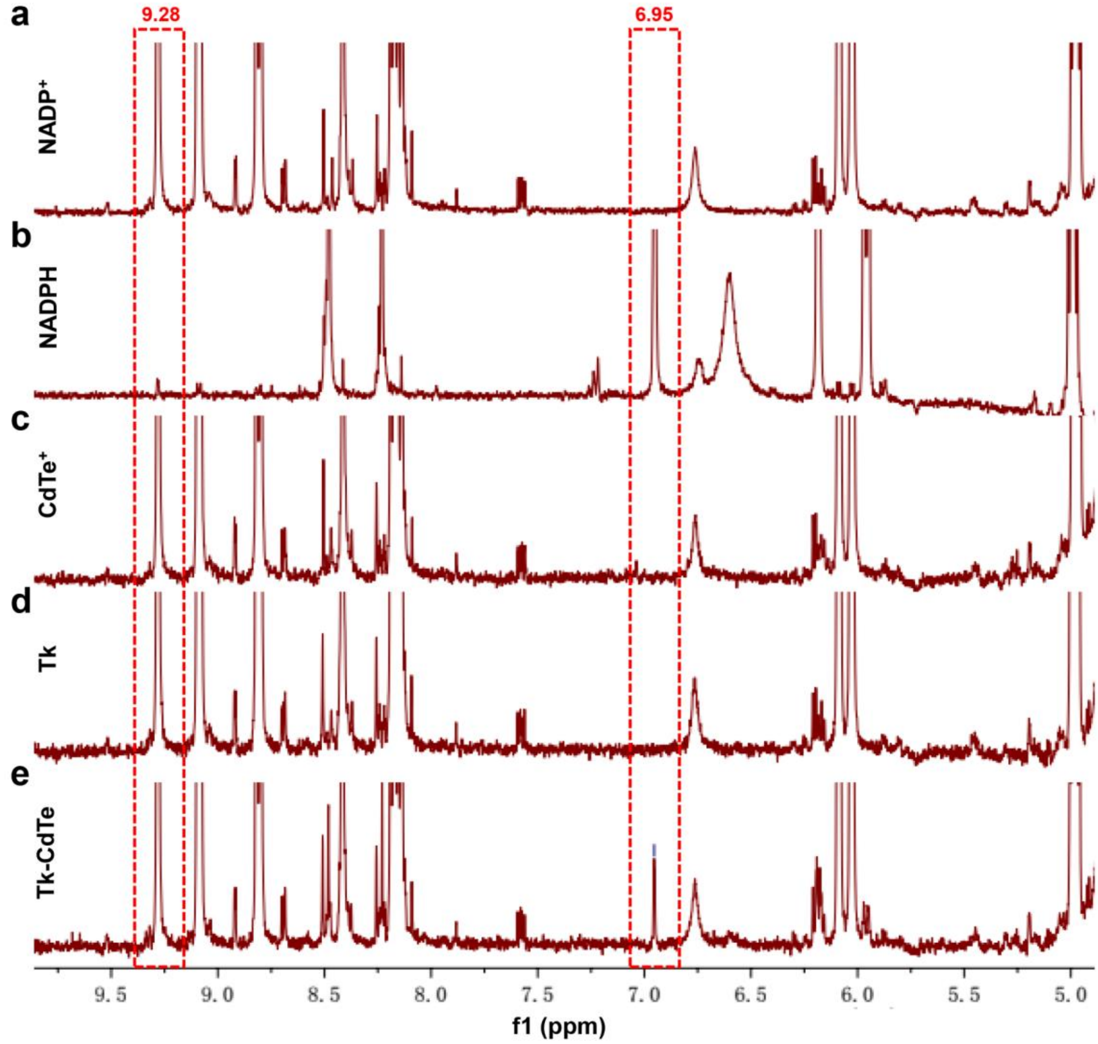

Supplementary Fig. 28 | Bioactive assay of regenerated NADPH. The ^{1}H NMR spectra of standard (3 mM) (**a**) $NADP^+$ and (**b**) NADPH, and the regenerated NADPH with $NADP^+$ as a substrate in the presence of (**c**) $CdTe^+$, (**d**) Tk or (**e**) Tk–CdTe 1:1 at 15 μg/mL Chl equivalent after 30 min illumination.

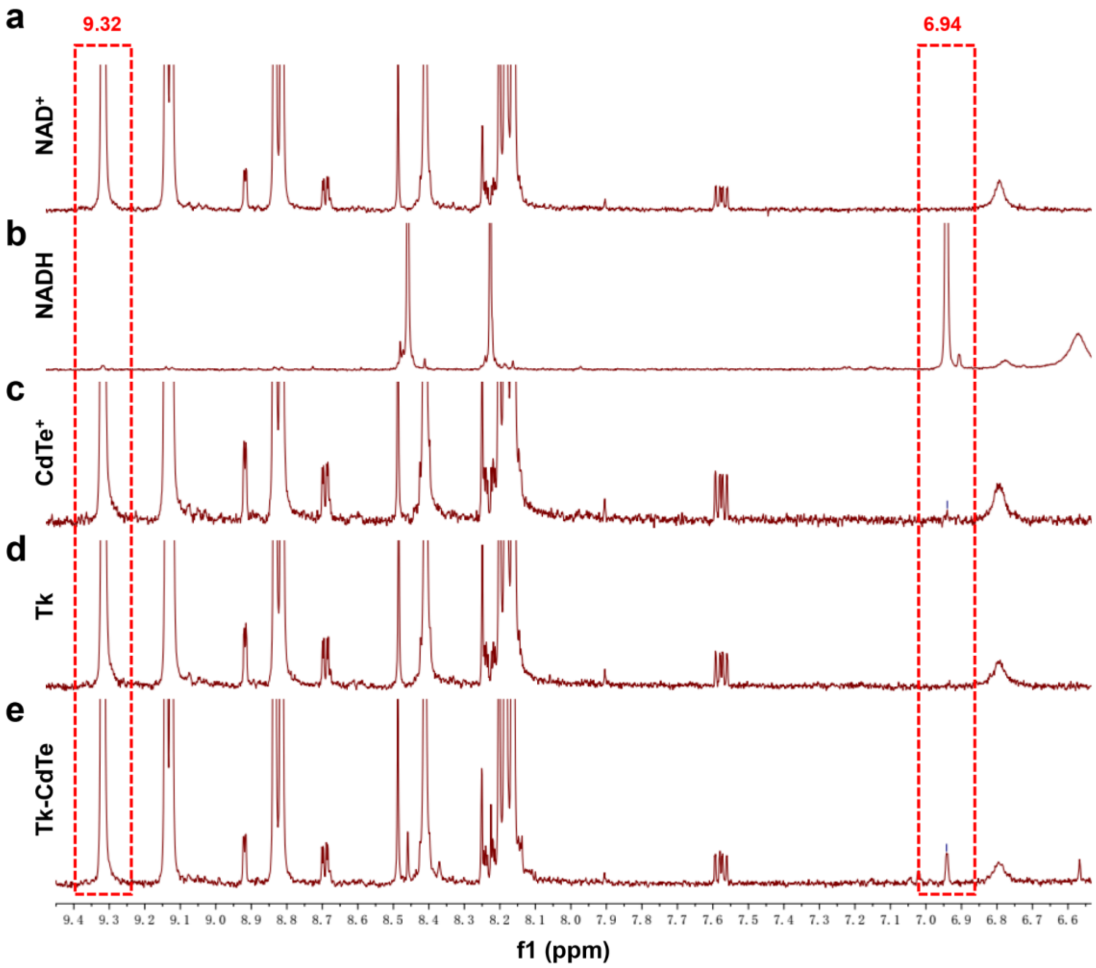

Supplementary Fig. 29 | Bioactive assay of regenerated NADH. The ^{1}H NMR spectra of standard (3 mM) (**a**) NAD^+ and (**b**) NADH, and the regenerated NADH with NAD^+ as a substrate in the presence of (**c**) $CdTe^+$, (**d**) Tk or (**e**) Tk–CdTe 1:1 at 15 μg/mL Chl equivalent after 30 min illumination.

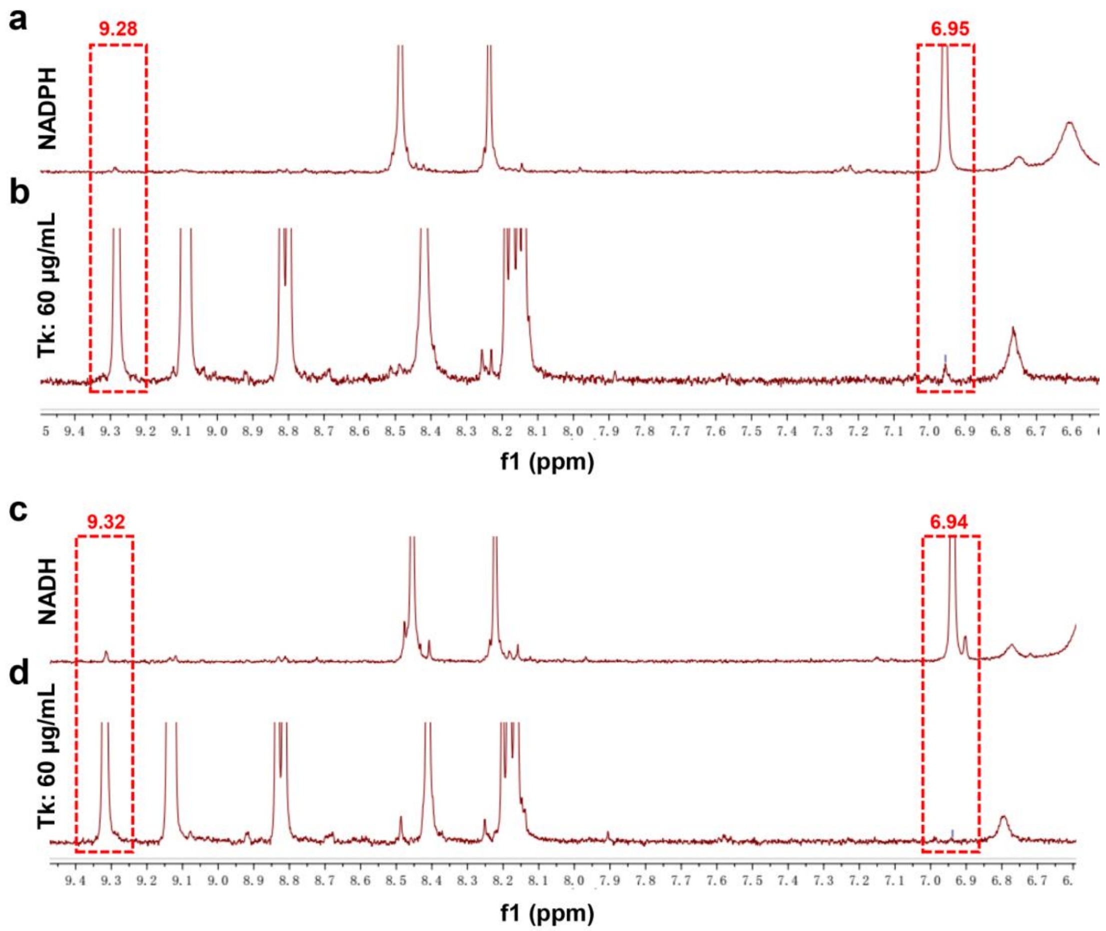

Supplementary Fig. 30 | Bioactive assay of regenerated cofactors by Tk. The ^{1}H NMR spectra of standard (3 mM) (**a**) NADPH or (**c**) NADH, and the regenerated (**b**) NADPH or (**d**) NADH with corresponding $NADP^+$ or NAD^+ as a substrate in the presence of Tk at 60 μg/mL Chl equivalent after 30 min illumination.

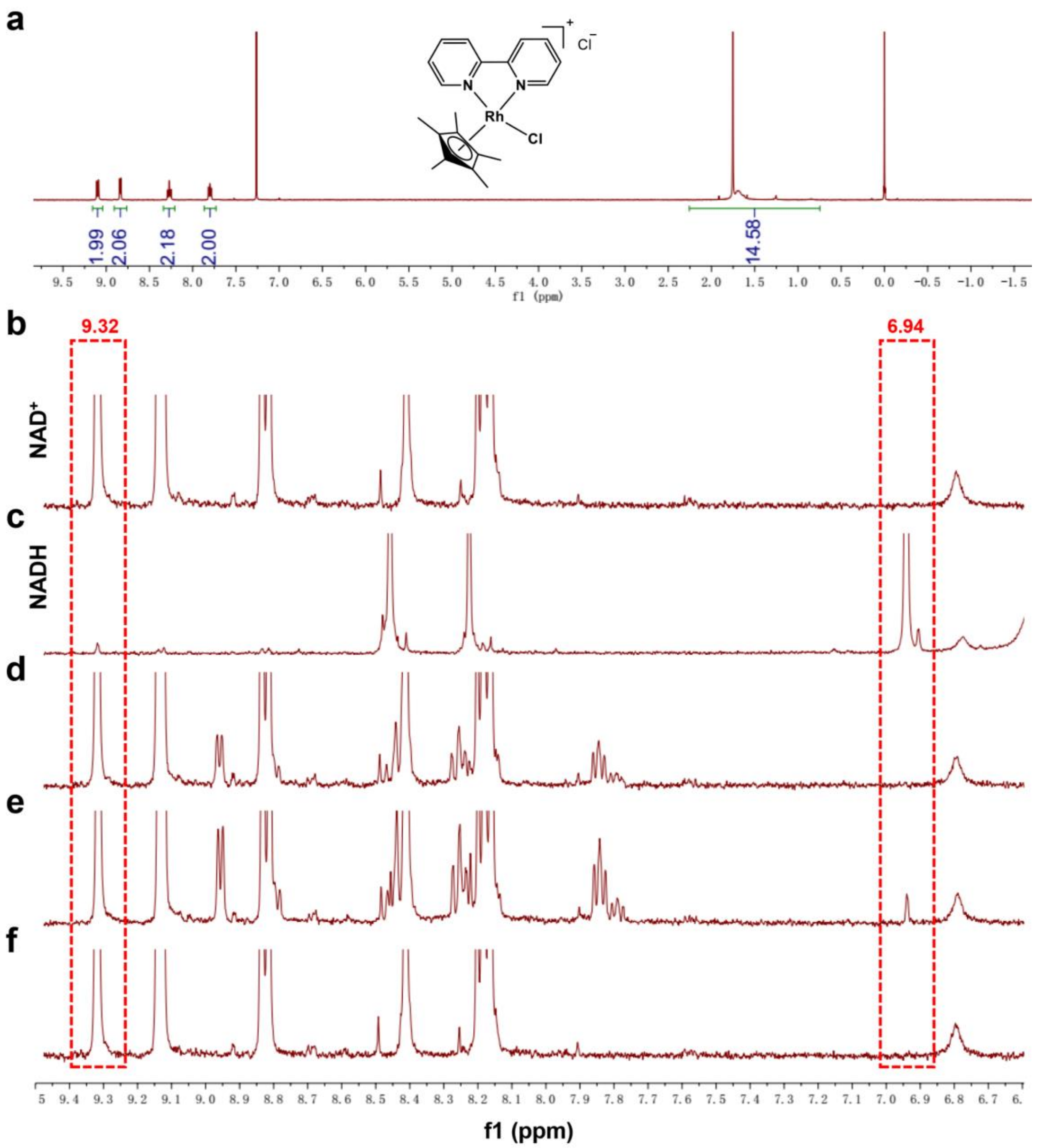

Supplementary Fig. 31 | Electronic mediator [Cp*Rh(bpy)H_2O]$^{2+}$ (denoted as [M]) showing a promoted effect on NADH regeneration by CdTe$^+$. (**a**) The ^{1}H NMR spectrum of [Cp*Rh(bpy)Cl]Cl in $CDCl_3$. (**b–f**) The ^{1}H NMR spectrum of regenerated NADH with NAD$^+$ as a substrate in the presence of CdTe$^+$ and [M] after 30 min illumination. Standard spectra of (**b**) NAD$^+$ and (**c**) NADH. Spectra in the presence of (**d**) CdTe$^+$ at 12.96 μg/mL and 0.2 mM [M], (**e**) CdTe$^+$ at 51.84 μg/mL and 0.4 mM [M] and (**f**) CdTe$^+$ at 51.84 μg/mL.

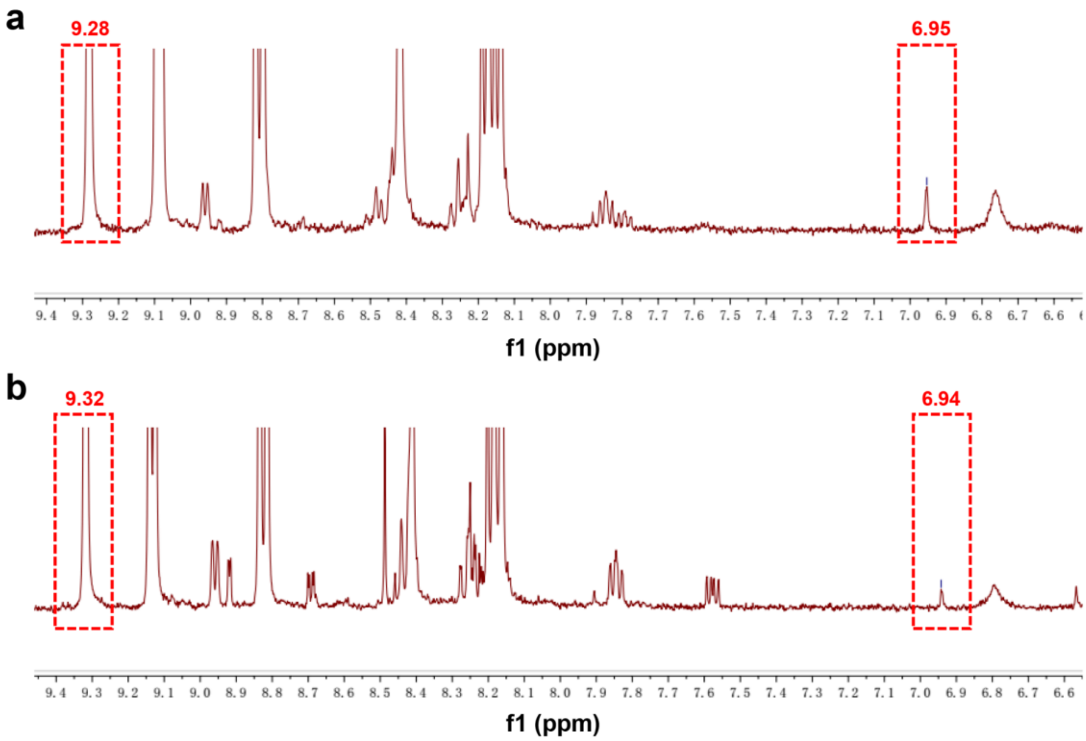

Supplementary Fig. 32 | Electronic mediator [Cp*Rh(bpy)H_2O]$^{2+}$ showing negligible effect on NADH regeneration by Tk–CdTe. (**a, b**) The ^{1}H NMR spectrum of regenerated (**a**) NADPH and (**b**) NADH with corresponding $NADP^+$ and NAD^+ as a substrate in the presence of Tk–CdTe 1:1 at 15 μg/mL Chl equivalent after 30 min illumination.

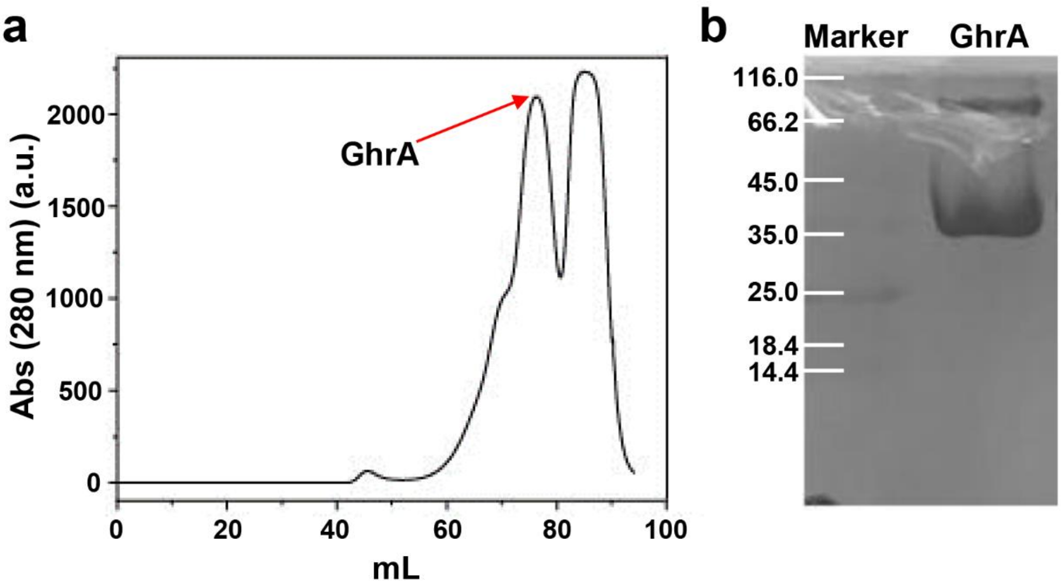

Supplementary Fig. 33 | Characterizations of glyoxylate/hydroxypyruvate reductase (GhrA). (**a**) Size-exclusion chromatography and (**b**) SDS−PAGE analysis of purified GhrA.

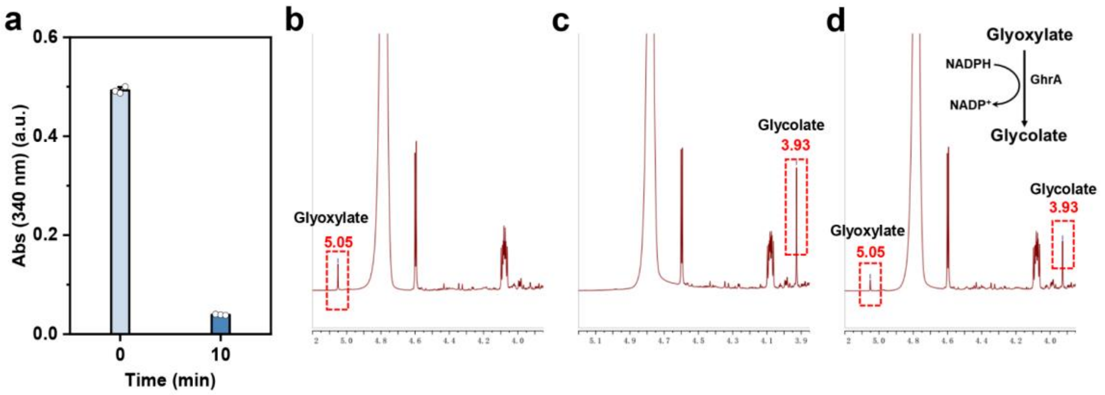

Supplementary Fig. 34 | Enzymatic activity assay of GhrA. (**a**) The time-dependent absorption at 340 nm with glyoxylate as a substrate in the presence of GhrA and NADPH. Data points are reported as mean ± standard deviation (n = 3). The ^{1}H NMR spectra of (**b**) glyoxylate, (**c**) glycolate and (**d**) regenerated glycolate with glyoxylate as a substrate in the presence of GhrA and NADPH.

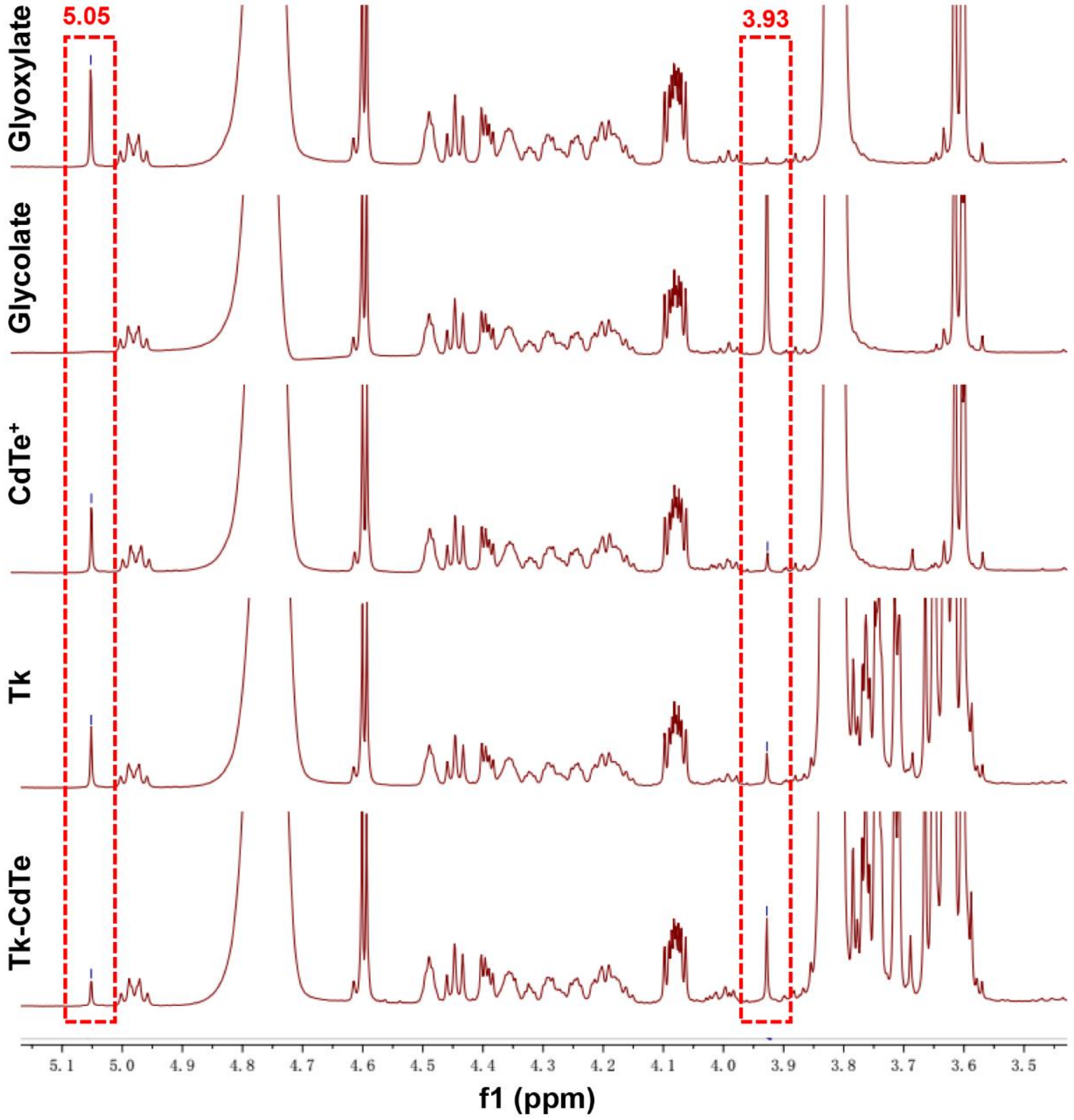

Supplementary Fig. 35 | Bioactivity assay of regenerated NADPH. The ^{1}H NMR spectra of standard (**a**) glyoxylate and (**b**) glycolate, and the regenerated glycolate with glyoxylate as a substrate in the presence of GhrA, NADP$^+$ and (**c**) CdTe$^+$, (**d**) Tk or (**e**) Tk–CdTe 1:1 at 30 μg/mL Chl equivalent after 30 min illumination.

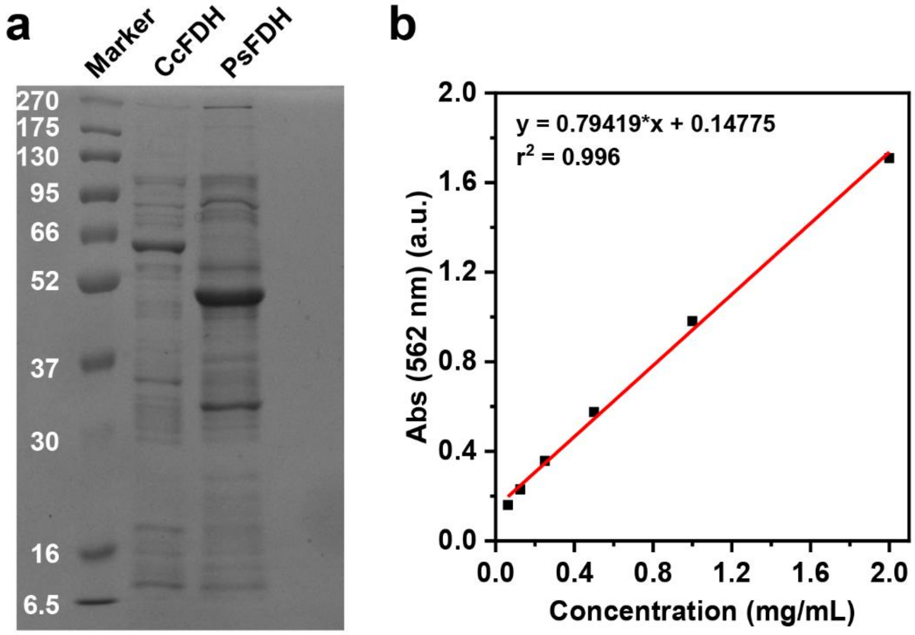

Supplementary Fig. 36 | Characterizations of formate dehydrogenases. (**a**) SDS−PAGE analysis of formate dehydrogenases. (**b**) The relationship of protein absorbance at 562 nm *versus* its concentration in a BCA kit method for quantitative analysis of formate dehydrogenases.

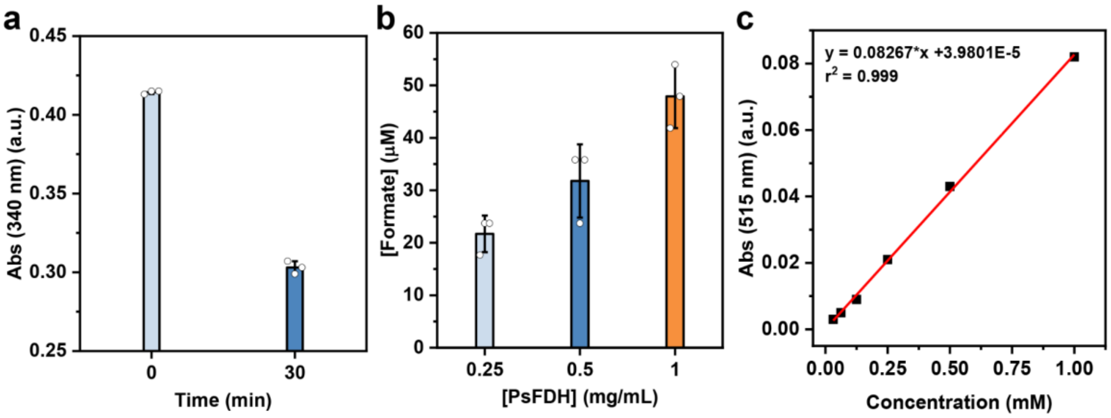

Supplementary Fig. 37 | Enzymatic activity assay of PsFDH. (**a**) The time-dependent absorption at 340 nm with CO_2 as a substrate in the presence of PsFDH and NADPH. Data points are reported as mean ± standard deviation (n = 3). (**b**) Enzymatic conversion of CO_2 to formate with different concentration of PsFDH in the presence of NADPH. Data points are reported as mean ± standard deviation (n = 3). (**c**) The relationship of product absorbance at 515 nm *versus* formate concentration in the Lang and Lang method for quantitative analysis of formate concentration.

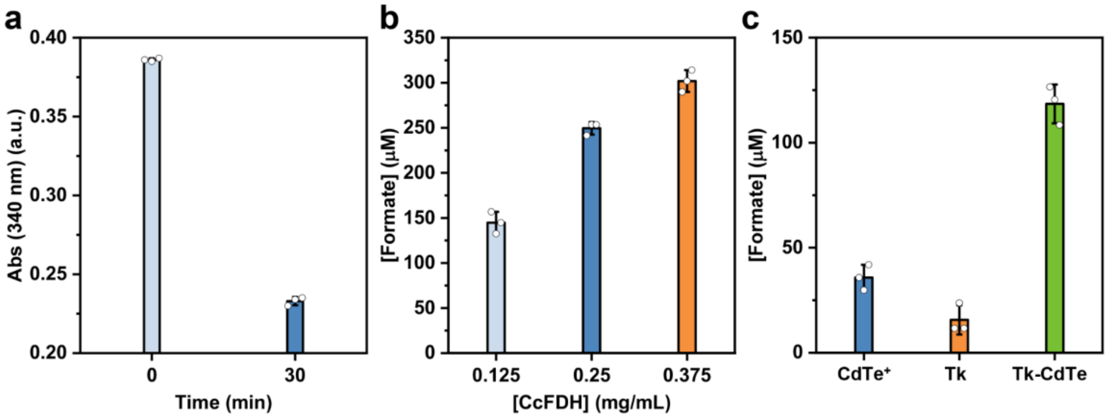

Supplementary Fig. 38 | Enzymatic activity assay of CcFDH. (**a**) The time-dependent absorption at 340 nm with $NaHCO_3$ as a substrate in the presence of CcFDH and NADH. Data points are reported as mean ± standard deviation (n = 3). (**b**) Enzymatic conversion of $NaHCO_3$ to formate with different concentration of CcFDH in the presence of NADH. Data points are reported as mean ± standard deviation (n = 3). (**c**) Photoenzymatic conversion of $NaHCO_3$ to formate by CcFDH coupled with $CdTe^+$, Tk or Tk–CdTe 1:1 in the presence of NAD^+ under 1 h illumination. Data points are reported as mean ± standard deviation (n = 3).

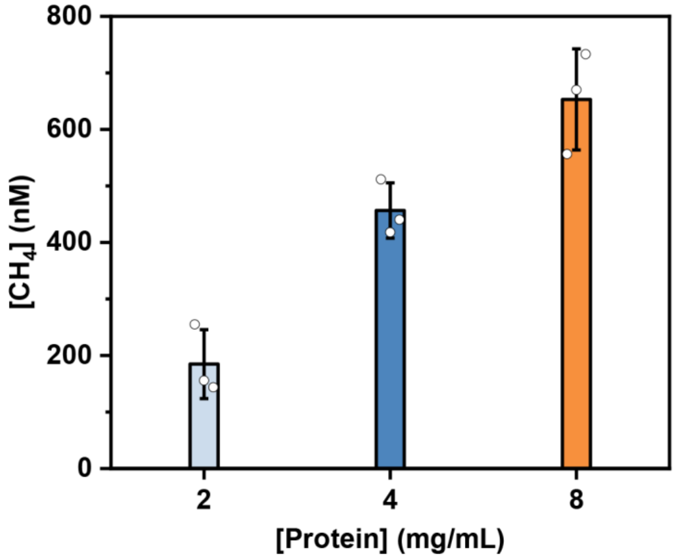

Supplementary Fig. 39 | Enzymatic conversion of CO_2 to methane with different concentration of MoFe crude extracts (protein) in the presence of ATP. Data points are reported as mean ± standard deviation (n = 3).

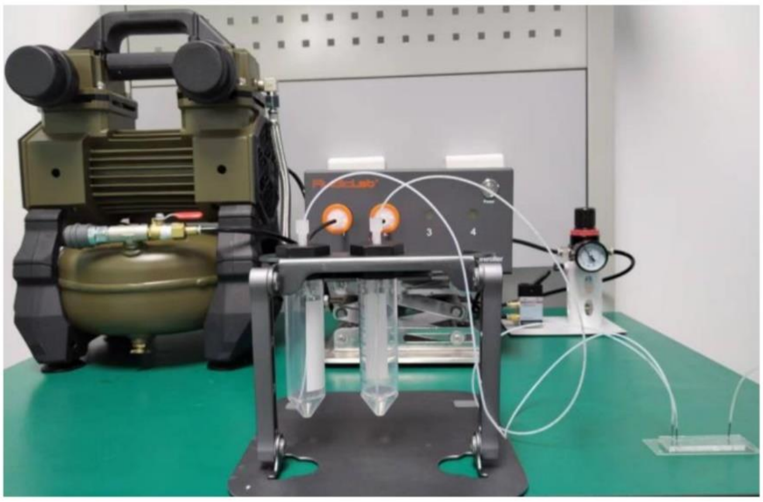

Supplementary Fig. 40 | The photograph of microfluidic device for the fabrication of artificial photosynthetic cells. The device contains pressure-driven pump, pressure source handle, pressure controller, liquid reservoir and microfluidic chip.

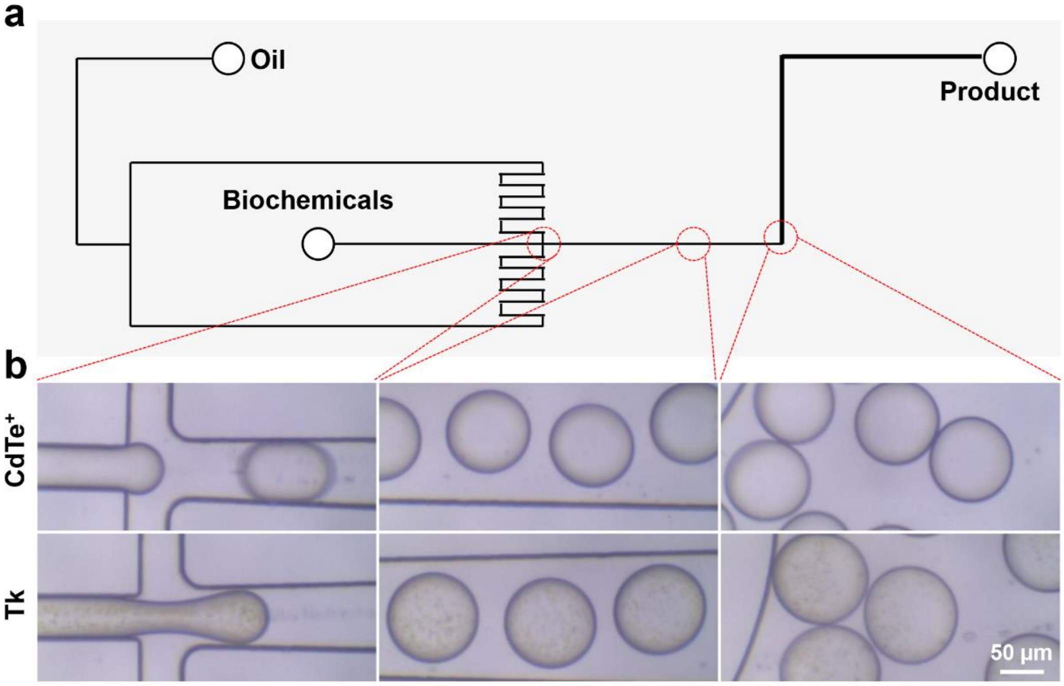

Supplementary Fig. 41 | The fabrication process of artificial photosynthetic cells for NADH regeneration in a microfluidic chip. (**a**) Schematic illustration of channels and components in a microfluidic chip. (**b**) Photographs of $CdTe^{+}$- or Tk-based artificial photosynthetic cells which flow through different area in channels.

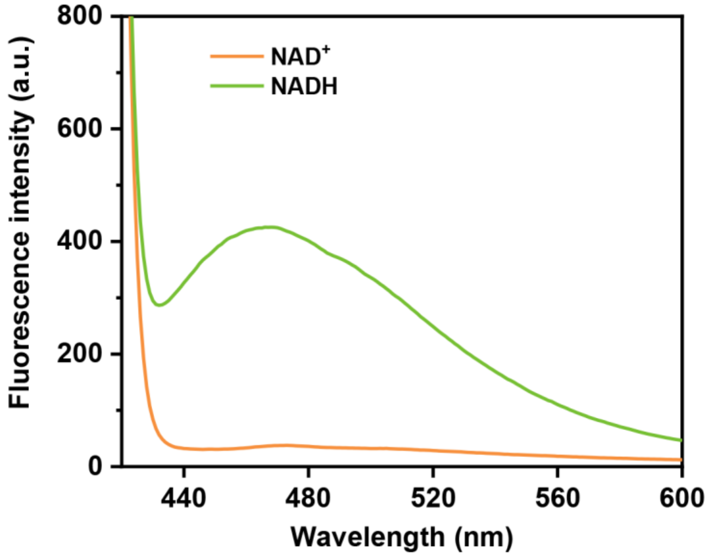

Supplementary Fig. 42 | Fluorescence spectra of NADH or NAD^+ in buffer with an excitation wavelength of 405 nm.

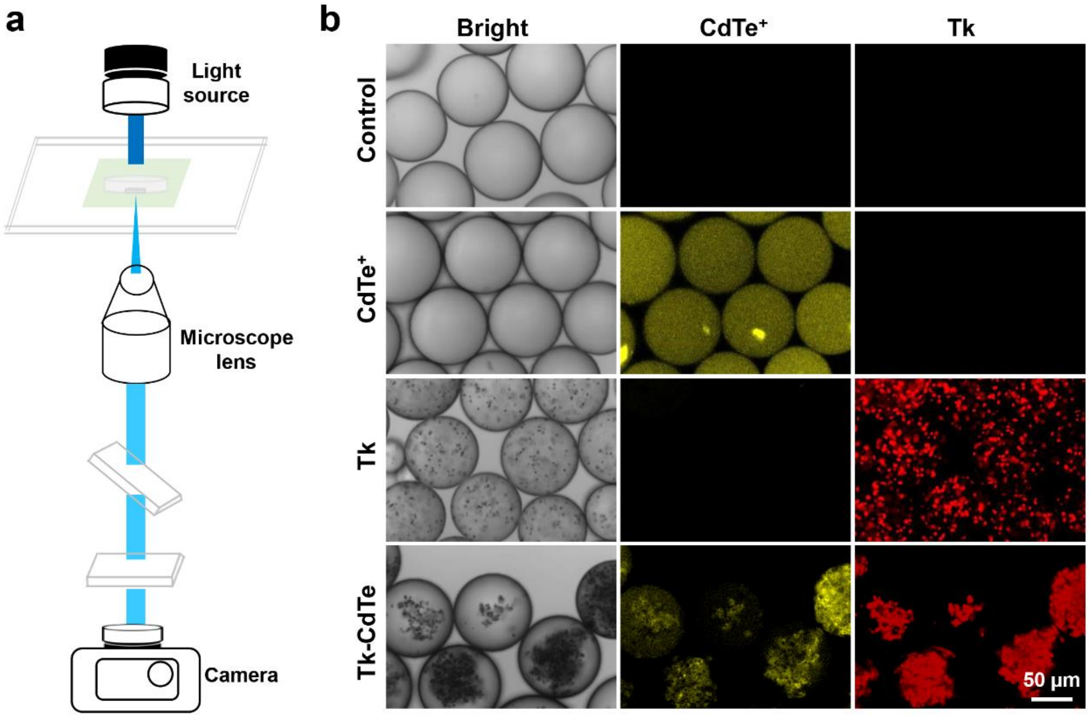

Supplementary Fig. 43 | CLSM images of artificial photosynthetic cells for NADH regeneration. (**a**) Simplified optical setup. (**b**) Images of artificial photosynthetic cells with Tk, $CdTe^{+}$ or Tk–CdTe. To identify thylakoid and CdTe QDs, an excitation laser of 488 nm or 405 nm was used, respectively. Tk and $CdTe^{+}$ are indicated by red and yellow fluorescence signals, respectively. As a control, the counterpart only containing buffer E is included.

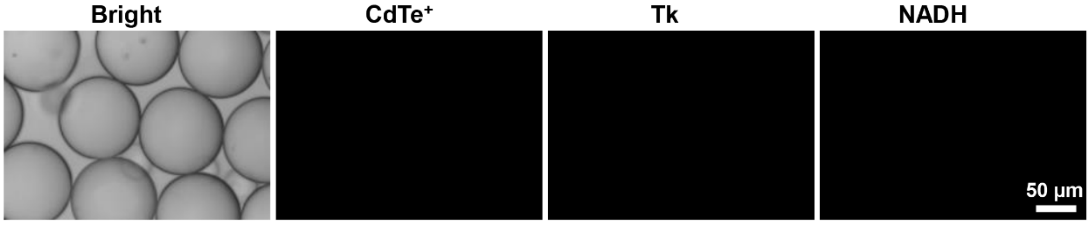

Supplementary Fig. 44 | CLSM images of NADH regeneration in artificial photosynthetic cells (control group) after 10 min light illumination. Regenerated NADH was detected with an excitation wavelength of 405 nm.

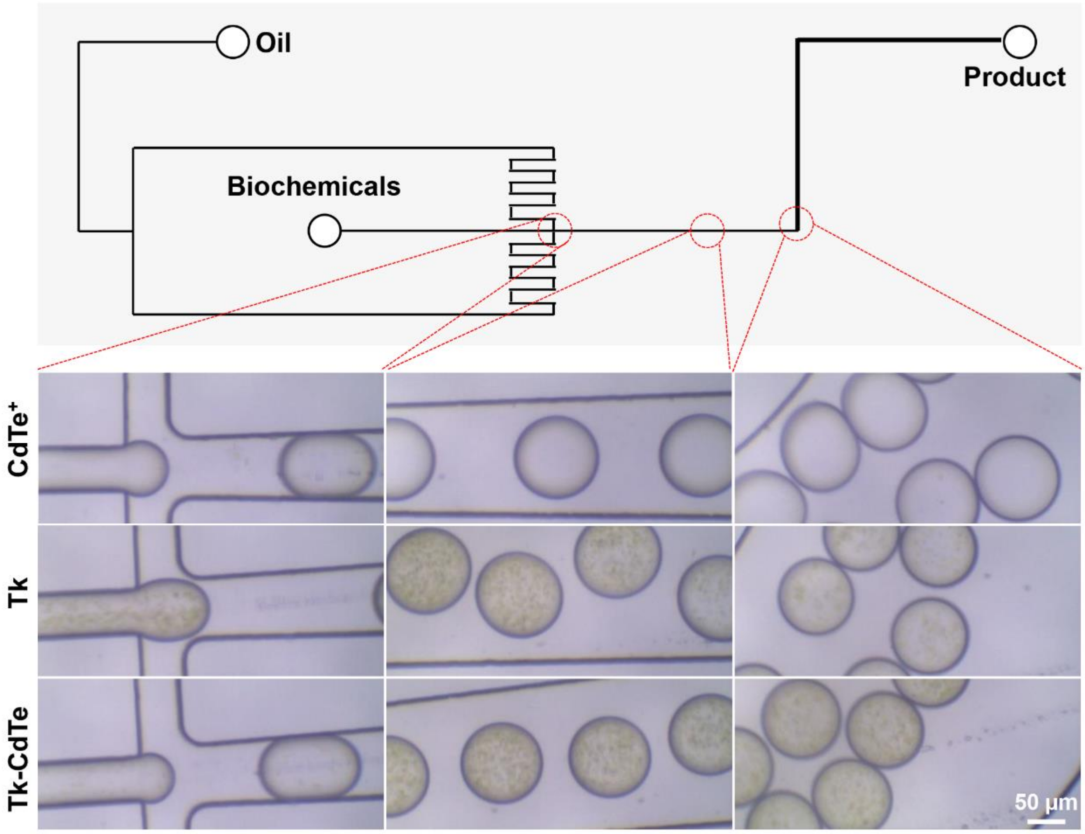

Supplementary Fig. 45 | The fabrication process of artificial photosynthetic cells for photoenzymatic conversion of CO_2 to formate with CcFDH in a microfluidic chip. Photographs of $CdTe^{+}$-, Tk- or Tk–CdTe-based artificial photosynthetic cells which flow through different area in channels.

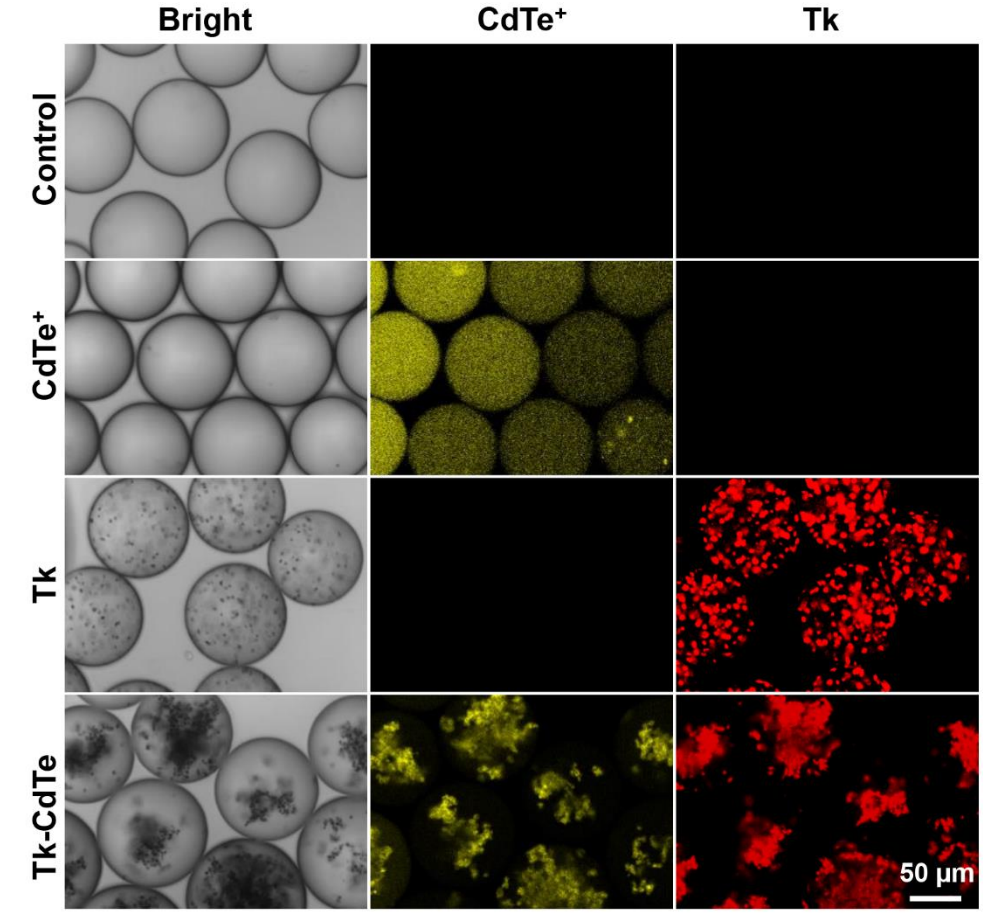

Supplementary Fig. 46 | CLSM images of artificial photosynthetic cells for photoenzymatic conversion of CO_2 to formate with CcFDH. Tk and $CdTe^+$ are indicated by red and yellow fluorescence signals, respectively. As a control, the counterpart only containing buffer G and CcFDH is included.

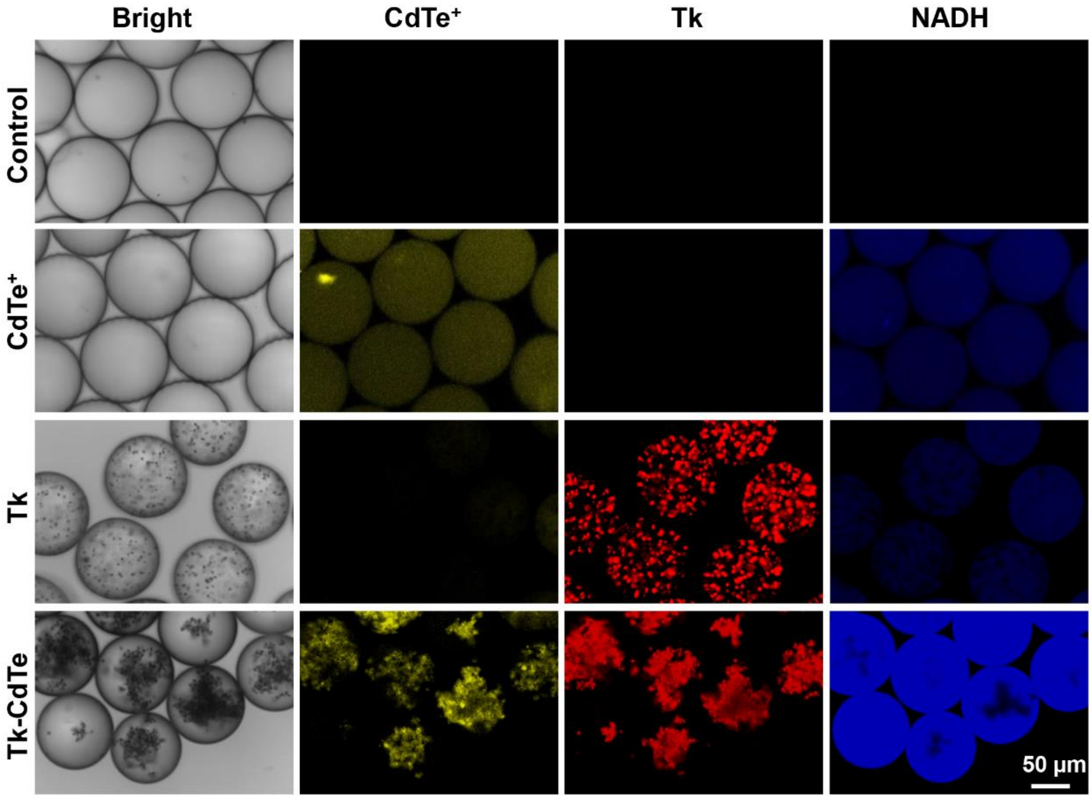

Supplementary Fig. 47 | CLSM images of NADH regeneration in artificial photosynthetic cells, which had been used for photoenzymatic conversion of CO_2 to formate with CcFDH, after 1 hour light illumination. Regenerated NADH was detected with an excitation wavelength of 405 nm.

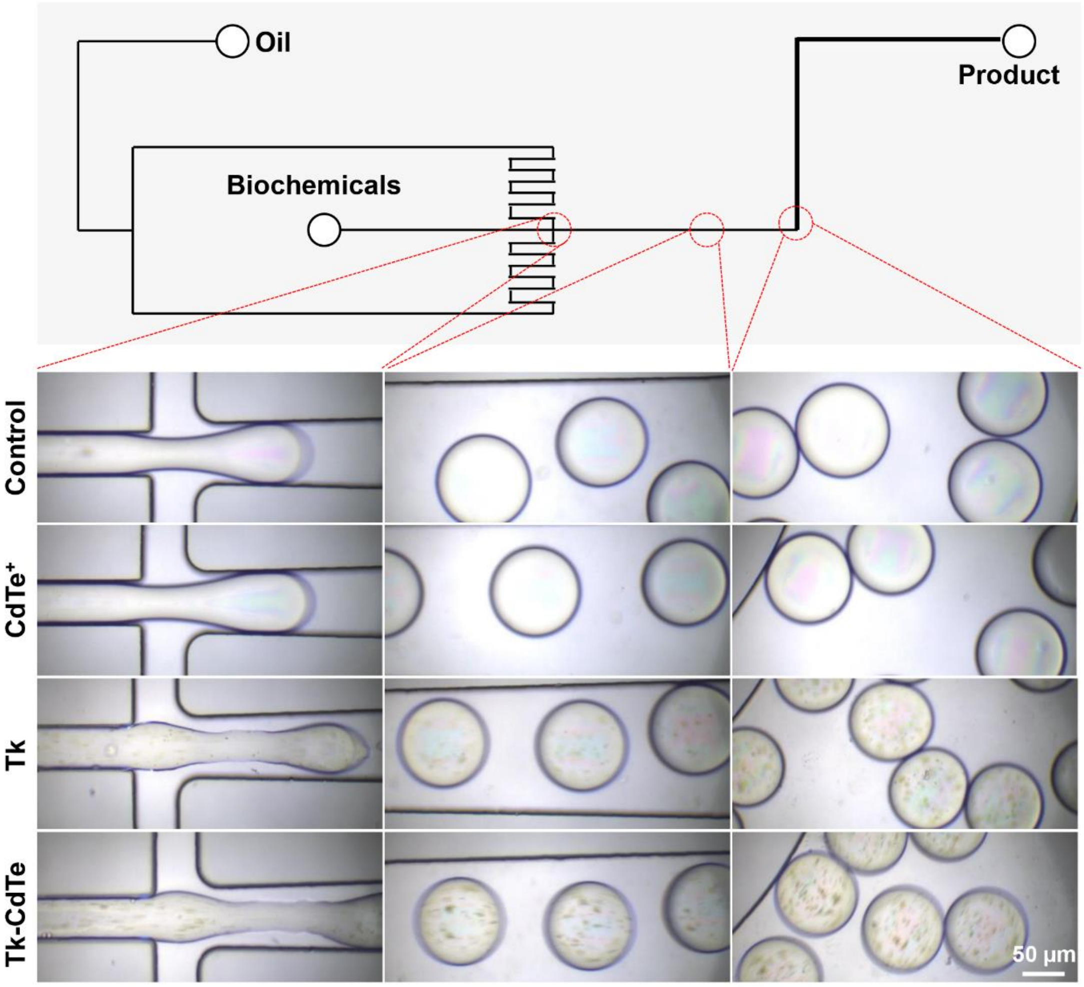

Supplementary Fig. 48 | The fabrication process of artificial photosynthetic cells for NADPH and ATP regeneration in a microfluidic chip. (**a**) Schematic illustration of channels and components in a microfluidic chip. (**b**) Photographs of artificial photosynthetic cells which flow through different area in channels.

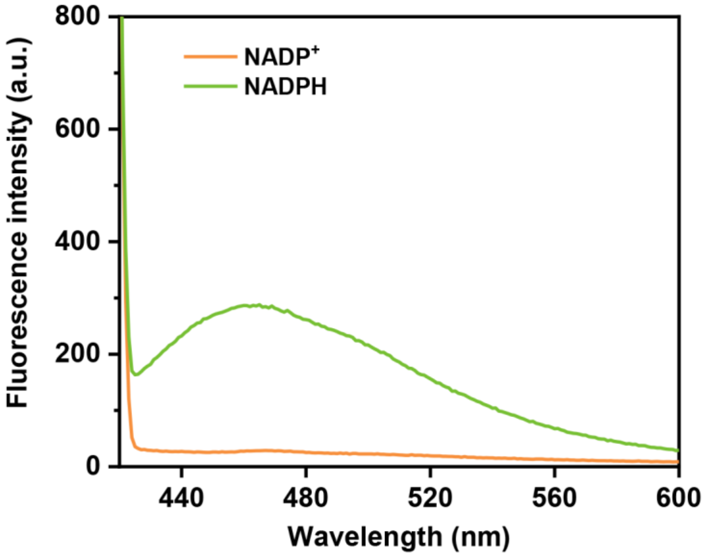

Supplementary Fig. 49 | Fluorescence spectra of NADPH or NADP^{+} in buffer with an excitation wavelength of 405 nm.

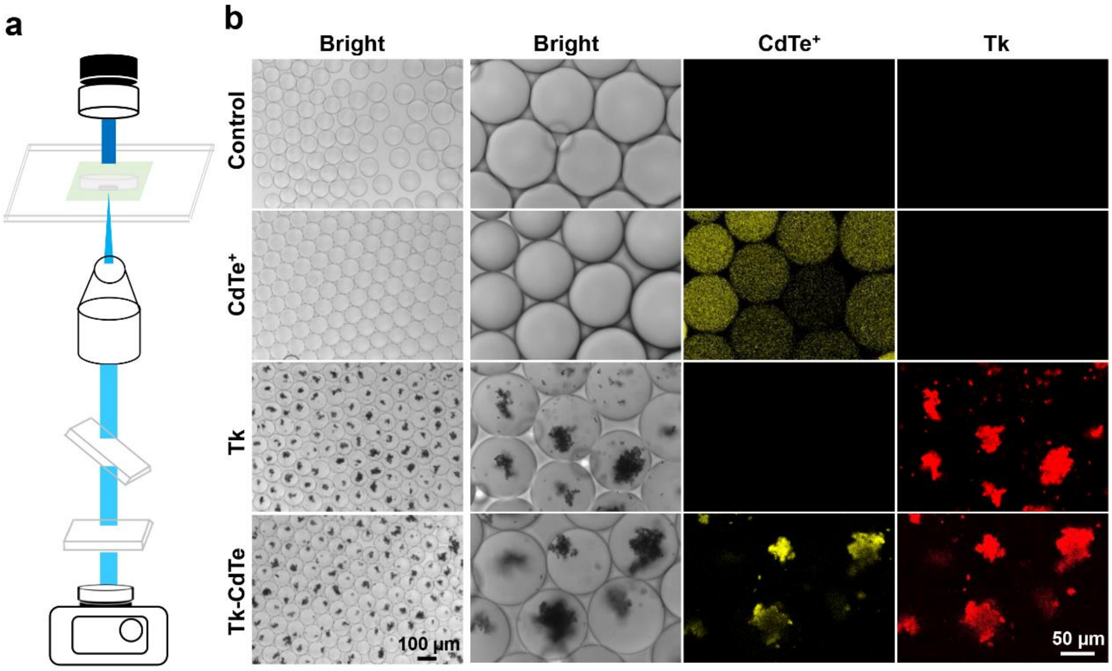

Supplementary Fig. 50 | CLSM images of artificial photosynthetic cells for NADPH and ATP regeneration. (**a**) Simplified optical setup. (**b**) Images of artificial photosynthetic cells with Tk, $CdTe^{+}$ or Tk–CdTe. To identify thylakoid and CdTe QDs, an excitation laser of 488 nm or 405 nm was used, respectively. Tk and $CdTe^{+}$ are indicated by red and yellow fluorescence signals, respectively. As a control, the counterpart only containing buffer F is included.

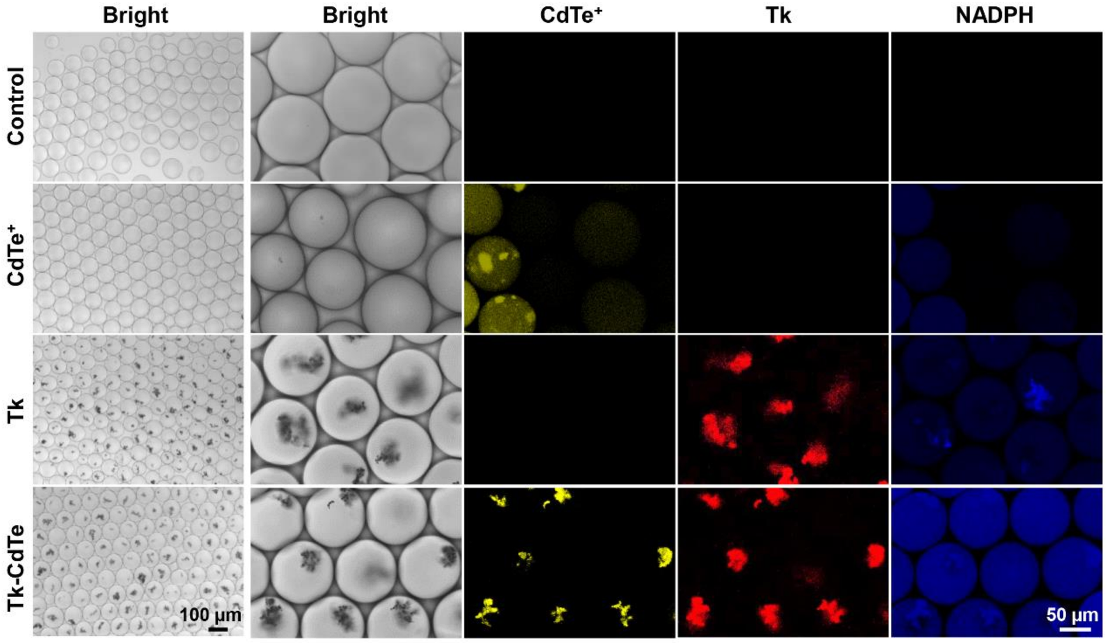

Supplementary Fig. 51 | CLSM images of NADPH regeneration in artificial photosynthetic cells after 10 min light illumination. Regenerated NADPH was detected with an excitation wavelength of 405 nm. As a control, the counterpart only containing buffer F is included.

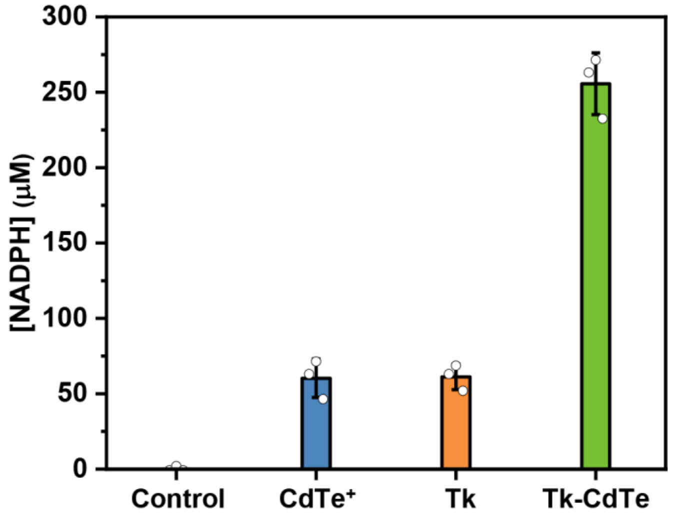

Supplementary Fig. 52 | NADPH regeneration by $CdTe^{+}$, Tk or Tk–CdTe with 50 μg/mL Chl equivalent in artificial photosynthetic cells after 10 min light illumination. The amounts of added $CdTe^{+}$ was 43.2 μg/mL. As a control, the counterpart only containing buffer F is included. Data points are reported as mean ± standard deviation (n = 3).

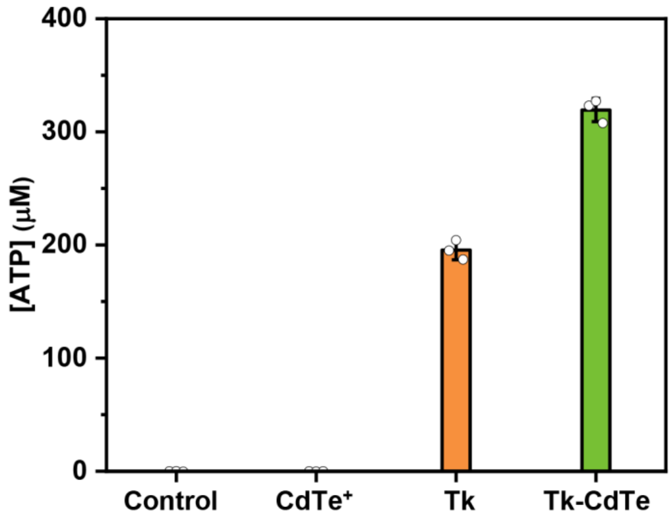

Supplementary Fig. 53 | ATP regeneration by $CdTe^+$, Tk or Tk–CdTe with 50 μg/mL Chl equivalent in artificial photosynthetic cells after 10 min light illumination. The amounts of added $CdTe^+$ was 43.2 μg/mL. As a control, the counterpart only containing buffer F is included. Data points are reported as mean ± standard deviation (n = 3).

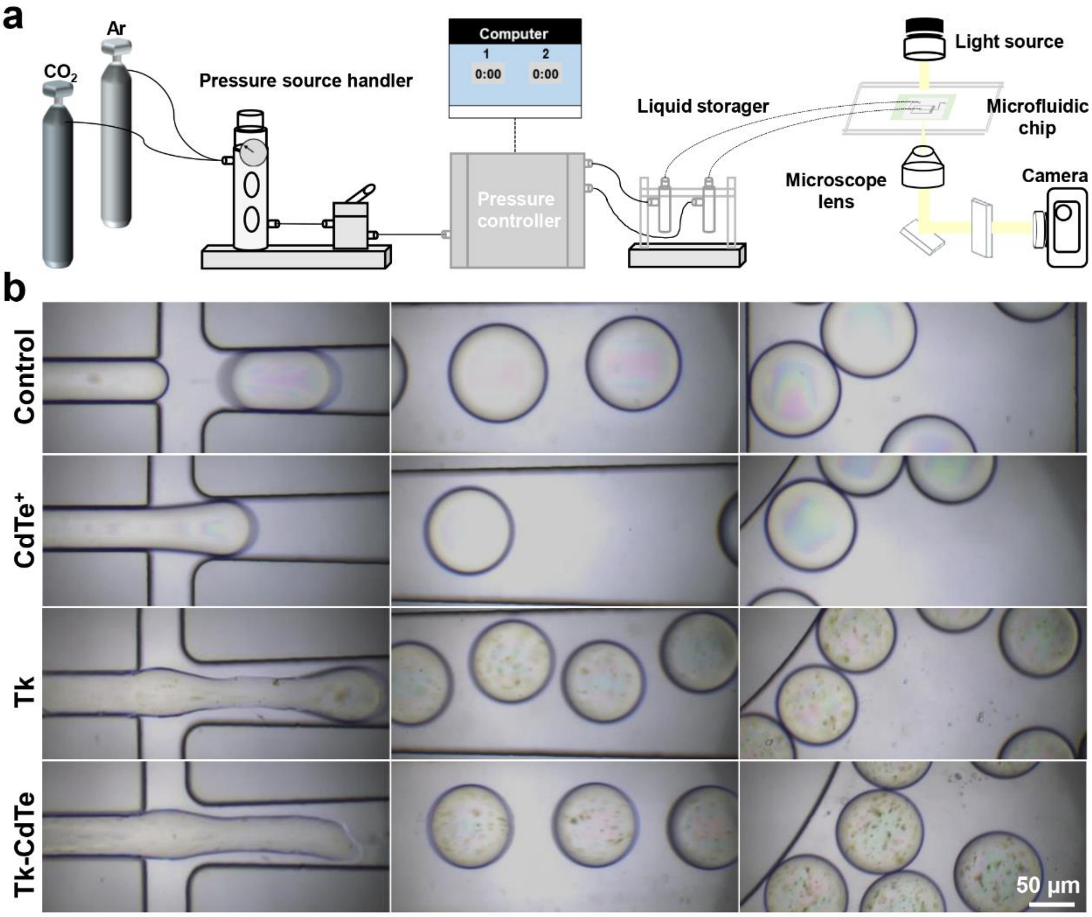

Supplementary Fig. 54 | The fabrication process of artificial photosynthetic cells for photoenzymatic conversion of CO_2 to formate or methane with PsFDH or MoFe in a microfluidic chip. (**a**) Schematic illustration of microfluidic device for artificial cells fabrication and real-time observation. (**b**) The fabrication process of artificial photosynthetic cells in a microfluidic chip. Photographs of $CdTe^+$-, Tk- or Tk–CdTe-based artificial photosynthetic cells which flow through different area in channels. As a control, the counterpart only containing buffer H, PsFDH and MoFe is included.

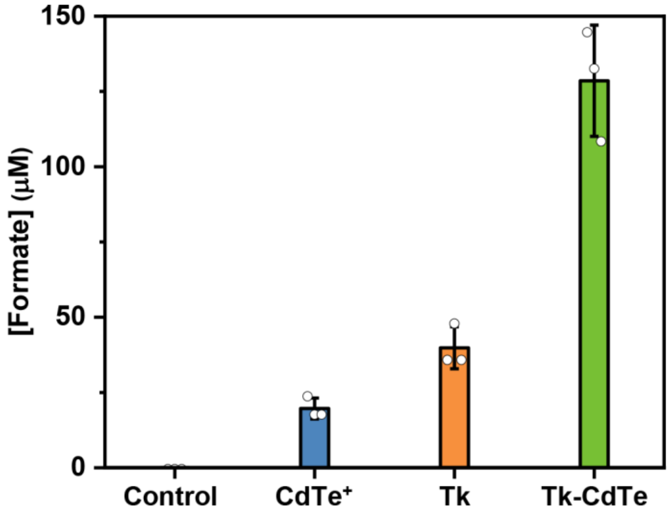

Supplementary Fig. 55 | Formate production from CO_2 with PsFDH by $CdTe^+$, Tk or Tk–CdTe in artificial photosynthetic cells after 4 h light illumination. Control denotes the counterpart only containing buffer H, PsFDH and MoFe. Data points are reported as mean ± standard deviation (n = 3).

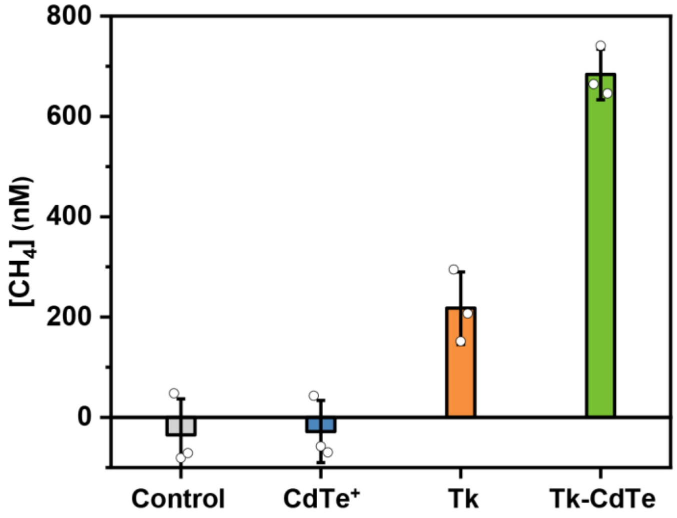

Supplementary Fig. 56 | Methane production from CO_2 with MoFe by $CdTe^+$, Tk or Tk–CdTe in artificial photosynthetic cells after 4 h light illumination. Control denotes the counterpart only containing buffer H, PsFDH and MoFe. Data points are reported as mean ± standard deviation (n = 3).

Supplementary Table 1 | CO_2 reductases and regenerated cofactors in the previously reported photoenzymatic CO_2 conversion systems.

CO_2 reductases*	Cofactors	References
Ccr-Pcc	NADPH/ATP	5
FDH	NADH	[45]
TsFDH	NADH	[46]
FDH	NADH	[47]
TsFDH-FaldDH-YADH	NADH	[48]
FDH	NADH	[49]
CcFDH-PcFaldDH-YADH	NADH	[17]
FDH-AlDH -ADH	NADPH	[50]
TsFDH	NADH	[51]
FDH	NADH	[52]
TsFDH	NADH	[53]
FateDH-FaldDH-ADH	NADH	[54]
FDH-F_{ald}DH-ADH	NADH	[55]
FDH	NADH	[56]
FDH	NADPH	[16]
FDH	NADH	[57]
ME	NADPH	[58]

*FDH/CcFDH/TsFDH/TsFDH/FateDH: formate dehydrogenase; F_{ald}DH/FaldDH/PcFaldDH: formaldehyde dehydrogenase; ADH/YADH: alcohol dehydrogenase; AlDH: aldehyde dehydrogenase; ME: malic enzyme; Ccr: crotonyl-coenzyme A (CoA) carboxylase/reductase; Pcc: propionyl-CoA carboxylase.

Supplementary Table 2 | A summary on the element contents and loading efficacy in Tk−CdTe determined by ICP−AES.

Sample	Cd (μg/mL)	Te (μg/mL)	Cd/Te	CdTe (μg/mL)	Added (μg/mL)	Loading (%)
Tk−CdTe 2:1						
1	231.15	43.86	5.27	275.01	300	91.67
2	219.63	41.37	5.31	261	300	87
3	248.91	47.1	5.28	296.01	300	98.67
4	232.59	44.4	5.24	276.99	300	92.33
Mean ± s.d.	233.07 ± 12.05	44.18 ± 2.35	5.28 ± 0.03	277.25 ± 14.39	300	92.42 ± 4.8
Tk−CdTe 1:1						
1	352.83	67.14	5.26	419.97	500	83.99
2	373.62	71.37	5.23	444.99	500	89
3	357.09	67.92	5.26	425.01	500	85
4	367.71	70.26	5.23	437.97	500	87.59
Mean ± s.d.	362.82 ± 9.54	69.17 ± 1.98	5.25 ± 0.01	431.99 ± 11.51	500	86.40 ± 2.3
Tk−CdTe 1:2						
1	467.91	89.13	5.25	557.04	750	74.27
2	490.89	93.18	5.27	584.07	750	77.87
3	500.52	95.43	5.24	596.95	750	79.47
4	476.76	91.2	5.23	567.96	750	75.73
Mean ± s.d.	484.02 ± 14.51	92.24 ± 2.70	5.25 ± 0.02	576.26 ± 17.19	750	76.83 ± 2.29

Supplementary Table 3 | Fitting parameters for the fluorescence decay curves of CdTe in Tk–CdTe.

Sample	T1 (B1)	T2 (B2)	T3 (B3)	τ1 (ns)	τ2 (ns)	τ3 (ns)	τ (ns)	χ^2
CdTe	14.50 (47.74%)	58.16 (28.63%)	3.30 (23.62%)	6.92	16.65	0.78	24.35	1.19
$CdTe^{+}$	14.85 (35.11%)	41.82 (53.68%)	2.54 (11.21%)	5.21	22.45	0.29	27.95	1.15
Tk–CdTe	5.34 (31.91%)	32.36 (17.85%)	0.24 (50.24%)	1.70	5.78	0.12	7.60	0.99

Supplementary Table 4 | Fitting parameters for the fluorescence decay curves of Tk in Tk–CdTe.

Sample	T1 (B1)	T2 (B2)	T3 (B3)	τ1 (ns)	τ2 (ns)	τ3 (ns)	τ (ns)	χ^2
Tk	1.30 (34.82%)	0.22 (62.24%)	5.64 (2.94%)	0.45	0.14	0.17	0.76	1.01
Tk-CdTe	1.35 (32.72%)	0.24 (61.45%)	8.41 (5.83%)	0.44	0.15	0.49	1.08	1.00

Supplementary Table 5 | A summary on the element contents and loading efficacy in PSII−CdTe that determined by ICP−AES.

Sample	Cd (μg/mL)	Te (μg/mL)	Cd/Te	CdTe (μg/mL)	Added (μg/mL)	Loading (%)
PSII−CdTe						
1	130.8	25.2	5.19	156	500	31.20
2	136.08	26.64	5.11	162.72	500	32.54
3	132.96	24.96	5.33	157.92	500	31.58
Mean ± s.d.	133.28 ± 2.65	25.6 ± 0.91	5.21 ± 0.11	158.88 ± 3.46	500	31.78 ± 0.69

Supplementary Table 6 | Fitting parameters for the fluorescence decay curves of CdTe in PSII–CdTe.

Sample	T1 (B1)	T2 (B2)	T3 (B3)	τ1 (ns)	τ2 (ns)	τ3 (ns)	τ (ns)	χ^2
CdTe	10.33 (37.72%)	37.06 (52.10%)	2.26 (10.18%)	3.90	19.31	0.23	23.44	1.19
$CdTe^+$	11.29 (35.00%)	43.00 (51.87%)	2.50 (13.12%)	3.95	22.30	0.33	26.58	1.09
PSII–CdTe	2.94 (10.81%)	18.40 (6.38%)	0.15 (82.82%)	0.32	1.17	0.12	1.61	1.02

Supplementary Table 7 | Fitting parameters for the fluorescence decay curves of PSII in PSII−CdTe.

Sample	T1 (B1)	T2 (B2)	T3 (B3)	τ1 (ns)	τ2 (ns)	τ3 (ns)	τ (ns)	χ^2
PSII	0.25 (50.60%)	1.13 (44.30%)	4.60 (5.10%)	0.13	0.50	0.23	0.86	1.01
PSII−CdTe	0.84 (74.12%)	2.32 (21.13%)	7.78 (4.75%)	0.62	0.49	0.37	1.48	1.08

Supplementary Table 8 | A summary on the element contents and loading efficacy in Tk−CdS that determined by ICP−AES.

Sample	Cd (μg/mL)	S (μg/mL)	Cd/S	CdS (μg/mL)	Added (μg/mL)	Loading (%)
Tk−CdS						
1	290.78	153.39	1.90	444.17	500	88.83
2	298.78	159.15	1.88	457.93	500	91.59
3	292.83	155.25	1.89	448.08	500	89.61
Mean ± s.d.	294.13 ± 4.15	155.93 ± 2.94	1.89 ± 0.01	450.06 ± 7.09	500	90.01 ± 1.42

Supplementary Table 9 | A summary on the element contents and loading efficacy in Tk–MoS_2 that determined by ICP–AES.

Sample	Mo (μg/mL)	S (μg/mL)	Mo/S	MoS_2 (μg/mL)	Added (μg/mL)	Loading (%)
Tk–MoS_2						
1	179.06	274.65	0.65	453.71	500	90.74
2	172.43	256.44	0.67	428.87	500	85.77
3	166.70	249.83	0.67	416.53	500	83.31
Mean ± s.d.	172.73 ± 6.18	260.31 ± 12.85	0.66 ± 0.01	433.04 ± 18.94	500	86.61 ± 3.79

Supplementary Table 10 | The reaction rate and quantum efficiency for CO_2 conversion to C1 product in our system compared to the state-of-the-art studies on photochemical CO_2 conversion.

Catalyst system	Reaction rate (μM mg^{-1} h^{-1})[i]	Quantum efficiency[ii]	References
Treated rape pollen (TRP)	1.4	AQE 0.32%	Energy Environ. Sci. 2018, 11, 2382-2389
Photosystem II/ FDH tandem cell	96.35 ± 8.85	AQE < 0.033 ± 0.004%	J. Am. Chem. Soc. 2018, 140, 16418−16422
CdS-PTi/ TsFDH−FaldDH−YADH	82.29 ± 6.17	IQE 0.40 ± 0.08%	ACS Catal. 2019, 9, 5, 3913–3925
ZnPc/BVNS	< 0.01	AQE < 0.3%	Angew. Chem. Int. Ed. 2019, 58, 10873 –10878
$CuIn_5S_8$ layers	N.P.[iii]	AQE 0.786%	Nat. Energy. 2019, 4, 690-699
$M_{0.33}WO_3$	< 18	N.P.	J. Am. Chem. Soc. 2019, 141, 13, 5267–5274
TPE-C_3N_4/ FDH	177.25	AQE 0.227 ± 0.047%	ACS Appl. Mater. Interfaces 2020, 12, 34795−34805
^{m}CD/CN	0.099	IQE 2.1%	Nat Commun. 2020, 11, 2531
Reduced titania-Cu_2O	N.P.	AQE 0.012%	Appl. Catal. B. 2020, 279, 119344
3DOM CdSQD/NC	104.2	AQE 2.9%	Adv. Mater. 2021, 33, 2102690
COF/FDH	< 9.78	AQE < 0.013%	Angew. Chem. Int. Ed. 2022, 61, e202200261
15CN@M	< 0.1	AQE 1.7%	J. Am. Chem. Soc. 2022, 144, 9576–9585
GCE/*a*-CD	111.46	AQE 0.2 ± 0.1%	J. Am. Chem. Soc. 2022, 144, 14207–14216
2H-WSe_2	N.P.	IQE 0.23%	Nat Commun. 2022, 13, 1256
Tk-CdTe/FDH	201.84 ± 23.89	IQE 2.46 ± 0.19%	This work

[i]Reaction rate was calculated as follows:

$$\text{Reaction rate} = \frac{\text{Concentration of C1 product}}{\text{Mass of catalyst system} \times \text{Illumination time}}$$

Note: Mass of catalyst system represents the mass of photocatalyst or the sum mass of light-absorption materials and CO_2 reductase.

[ii]AQE: apparent quantum efficiency; IQE: internal quantum efficiency.

[iii]N.P.: not provided.

Supplementary Table 11 | Biochemical components of artificial photosynthetic cells in this work.

	Modules in artificial photosynthetic cells			
**Measurements**	**Control**	**CdTe⁺**	**Tk**	**Tk–CdTe**
NADH regeneration	Buffer E[1]	Buffer E + $CdTe^+$	Buffer E + Tk	Buffer E + Tk–CdTe
NADPH and ATP regeneration	Buffer F[2]	Buffer F + $CdTe^+$	Buffer F + Tk	Buffer F + Tk–CdTe
CO_2 reduction with CcFDH	Buffer G[3] + CcFDH	Buffer G + $CdTe^+$ + CcFDH	Buffer G + Tk + CcFDH	Buffer G + Tk-CdTe[4] + CcFDH[5]
CO_2 reduction with PsFDH and MoFe	Buffer H[6] + PsFDH + MoFe	Buffer H + $CdTe^+$ + PsFDH + MoFe	Buffer H + Tk + PsFDH + MoFe	Buffer H + Tk-CdTe[7] + PsFDH[8] + MoFe[9]

[1]50 mM HEPES-KOH pH 7.8, 5 mM K_2HPO_4, 10 mM sodium L-ascorbate, 10 mM KCl, 5 mM $MgCl_2$, 700 mM sorbitol, 4 mM NAD^+.

[2]50 mM HEPES-KOH pH 7.8, 5 mM K_2HPO_4, 10 mM sodium L-ascorbate, 10 mM KCl, 5 mM $MgCl_2$, 700 mM sorbitol, 4 mM $NADP^+$, 100 mM ADP.

[3]50 mM HEPES-KOH pH 7.8, 5 mM K_2HPO_4, 10 mM sodium L-ascorbate, 10 mM KCl, 5 mM $MgCl_2$, 700 mM sorbitol, 4 mM NAD^+, 50 mM $NaHCO_3$.

[4]80 µg/mL Chl equivalent or equal amount $CdTe^+$ (i.e., Chl equivalent × loading efficacy; similarly treated hereinafter).

[5]NADH-dependent formate dehydrogenase, 0.5 mg/mL.

[6]50 mM HEPES-KOH pH 7.8, 5 mM K_2HPO_4, 10 mM sodium L-ascorbate, 10 mM KCl, 5 mM $MgCl_2$, 700 mM sorbitol, 4 mM $NADP^+$, 100 mM ADP, 100 mM sodium dithionite, 0.1mg/mL DTT, Ar, CO_2.

[7]50 µg/mL Chl equivalent.

[8]NADPH-dependent formate dehydrogenase, 2 mg/mL.

[9]ATP-dependent MoFe crude extracts, 16 mg/mL.

Supplementary Table 12 | Primers used in this work.

Primer	Sequence (5'-3')
nifD-F	ACATGCATGCATGACCGGTATGTCGCGCGAAGAGG
nifD-R	CGCGGATCCTCAGGCGCTGGCGGCGACTTTC
V70Aα-F	TACGCCGGTTCCAAAGGCGCCGTCTGGGGC
V70Aα-R	GGCGCCTTTGGAACCGGCGTAGGCGCAGCC
H195Gα-F	CGTTTCCCAGTCCCTGGGCCAACACATCGCC
H195Gα-R	TTGGCCCAGGGACTGGGAAACGCCGCG
NifA-puc57-F	CCCAAGCTTATGAATGCAACCATCCCTCAGCGCTCG
NifA-puc57-R	CGGGATCCTCAGATCTTGCGCATGTGGATGTTGAGGG
Ps-Mutations	GTTCATCTGCACTATACCTAGCGTCACCGCCTGCCGG AAAGCGTG

Supplementary Table 13 | List of DNA sequences in this work.

Gene	Sequence
**nifD**	ATGACCGGTATGTCGCGCGAAGAGGTTGAATCCCTCATCCAGGAAGTTCT<br>GGAAGTTTATCCCGAGAAGGCTCGCAAGGATCGTAACAAGCACCTGGCC<br>GTCAACGACCCGGCGGTTACCCAGTCCAAGAAGTGCATCATCTCCAACA<br>AGAAGTCCCAGCCCGGTCTGATGACCATCCGCGGCTGCGCCTACGCCGG<br>TTCCAAAGGCGTGGTCTGGGGCCCCATCAAGGACATGATCCACATCTCCC<br>ACGGTCCGGTAGGCTGCGGCCAGTATTCGCGCGCCGGCCGTCGTAACTAC<br>TACATCGGTACCACCGGTGTGAACGCCTTCGTCACCATGAACTTCACCTC<br>GGACTTCCAGGAGAAGGACATCGTGTTCGGTGGCGACAAGAAGCTCGCC<br>AAACTGATCGACGAAGTGGAAACCCTGTTCCCGCTGAACAAGGGTATCT<br>CCGTCCAGTCCGAGTGCCCGATCGGCCTGATCGGCGACGACATCGAATCC<br>GTGTCCAAGGTCAAGGGCGCCGAGCTCAGCAAGACCATCGTACCGGTCC<br>GTTGCGAAGGCTTCCGCGGCGTTTCCCAGTCCCTGGGCCACCACATCGCC<br>AACGACGCAGTCCGCGACTGGGTCCTGGGCAAGCGTGACGAAGACACC<br>ACCTTCGCCAGCACTCCTTACGATGTGGCCATCATCGGCGACTACAACAT<br>CGGCGGCGACGCCTGGTCTTCCCGCATCCTGCTGGAAGAAATGGGCCTG<br>CGTTGCGTAGCCCAGTGGTCCGGCGACGGCTCCATCTCCGAAATCGAGCT<br>GACCCCGAAGGTCAAGCTGAACCTGGTTCACTGCTACCGCTCGATGAAC<br>TACATCTCCCGTCACATGGAAGAGAAGTACGGTATCCCATGGATGGAGTA<br>CAACTTCTTCGGCCCGACCAAGACCATCGAGTCGCTGCGTGCCATCGCCG<br>CCAAGTTCGACGAGAGCATCCAGAAGAAGTGCGAAGAGGTCATCGCCA<br>AGTACAAGCCCGAGTGGGAAGCGGTGGTCGCCAAGTACCGTCCGCGCCT<br>GGAAGGCAAGCGCGTCATGCTCTACATCGGTGGCCTGCGTCCGCGCCAC<br>GTGATCGGCGCCTACGAAGACCTGGGCATGGAAGTGGTGGGTACCGGCT<br>ACGAGTTCGCCCACAACGACGACTATGACCGCACCATGAAAGAAATGGG<br>TGACTCCACCCTGCTGTACGATGACGTGACCGGCTACGAATTCGAAGAAT<br>TCGTCAAGCGCATCAAGCCCGACCTGATCGGCTCCGGTATCAAGGAGAA<br>GTTCATCTTCCAGAAGATGGGCATCCCCTTCCGTCAAATGCACTCCTGGG

	ATTATTCCGGCCCCTACCACGGCTTCGATGGCTTCGCCATCTTCGCCCGTG
	ACATGGACATGACCCTGAACAATCCGTGCTGGAAGAAACTGCAGGCTCC
	CTGGGAAGCTTCCGAAGGCGCCGAGAAAGTCGCCGCCAGCGCCTGA
**nifA**	ATGAATGCAACCATCCCTCAGCGCTCGGCCAAACAGAACCCGGTCGAAC
	TCTATGACCTGCAATTGCAGGCCCTGGCGAGCATCGCCCGCACGCTCAGC
	CGCGAACAACAGATCGACGAACTGCTCGAACAGGTCCTGGCCGTACTGC
	ACAATGACCTCGGCCTGCTGCATGGCCTGGTGACCATTTCCGACCCGGAA
	CACGGCGCCCTGCAGATCGGCGCCATCCACACCGACTCGGAAGCGGTGG
	CCCAGGCCTGCGAAGGCGTGCGCTACAGAAGCGGCGAAGGCGTGATCG
	GCAACGTGCTCAAGCACGGCAACAGCGTGGTGCTCGGGCGCATCTCCGC
	CGACCCGCGCTTTCTCGACCGCCTGGCGCTGTACGACCTGGAAATGCCGT
	TCATCGCCGTGCCGATCAAGAACCCCGAGGGCAACACCATCGGCGTGCT
	GGCGGCCCAGCCGGACTGCCGCGCCGACGAGCACATGCCCGCGCGCAC
	GCGCTTCCTGGAGATCGTCGCCAACCTGCTGGCGCAGACCGTGCGCCTG
	GTGGTGAACATCGAGGACGGCCGCGAGGCGGCCGACGAGCGCGACGAA
	CTGCGTCGCGAGGTGCGCGGCAAGTACGGCTTCGAGAACATGGTGGTGG
	GCCACACCCCCACCATGCGCCGGGTGTTCGATCAGATCCGCCGGGTCGCC
	AAGTGGAACAGCACCGTACTGGTCCTCGGCGAGTCCGGTACCGGCAAGG
	AACTGATCGCCAGCGCCATCCACTACAAGTCGCCGCGCGCGCACCGCCC
	CTTCGTCCGCCTGAACTGCGCCGCGCTGCCGGAAACCCTGCTCGAGTCC
	GAACTCTTCGGCCACGAGAAGGGCGCCTTCACCGGCGCGGTGAAGCAG
	CGCAAGGGGCGTTTCGAGCAGGCCGACGGCGGCACCCTGTTCCTCGACG
	AGATCGGCGAGATCTCGCCGATGTTCCAGGCCAAGCTGCTGCGCGTGCT
	GCAGGAAGGCGAGTTCGAGCGGGTCGGCGGCAACCAGACGGTGCGGGT
	CAACGTGCGCATCGTCGCCGCCACCAACCGCGACCTGGAAAGCGAGGTG
	GAAAAGGGCAAGTTCCGCGAGGACCTCTACTACCGCCTGAACGTCATGG
	CCATCCGCATTCCGCCGCTGCGCGAGCGTACCGCCGACATTCCCGAACTG
	GCGGAATTCCTGCTCGGCAAGATCGGCCGCCAGCAGGGCCGCCCGCTGA
	CCGTCACCGACAGCGCCATCCGCCTGCTGATGAGCCACCGCTGGCCGGG

CAACGTGCGCGAACTGGAGAACTGCCTGGAGCGCTCGGCGATCATGAGC
GAGGACGGCACCATCACCCGCGACGTGGTCTCGCTGACCGGGGTCGACA
ACGAGAGCCCGCCGCTCGCCGCGCCGCTGCCCGAGGTCAACCTGGCCGA
CGAGACCCTGGACGACCGCGAACGGGTGATCGCCGCCCTCGAACAGGCC
GGCTGGGTGCAGGCCAAGGCCGCGCGGCTGCTGGGCATGACGCCGCGG
CAGATCGCCTACCGCATCCAGACCCTCAACATCCACATGCGCAAGATCTG
A

CcFDH ATGTCAAATGGTATTCATGAAATAGATGATTGTGATTTAGTATTTATTTTTG
GCTATAACGGTGCGGATTCACATCCTATAGTTGCAAGAAGAATAGTAAATG
CAAAACGAAAGGGAGCTAAAATAGTAGTAACAGATCCACGTATAACAGA
ATCGGCTAGGATAGCTGATTTATGGCTTCCTATAAAAAATGGCACTAATAT
GATACTAGTAAATGCTTTTGCAAATGTTTTAATAAATGAAGGCTTGTACAA
CAAGCAATATGTTGAAGAACATACAGTAGGCTTTGAAGAATATAAAGCTT
TAGTTGAAAAGTATACTCCAGAGTATGCAGAGAAAATGACTGGAGTACCA
GCAGAAGATATAAGAAAATCAATGAGAATGTACTCTAAAGCTAAAAATGC
TATGATACTTTATGGTATGGGAGTATGTCAATTTGGACAGGCGGTTGATGT
AGTTAAAGGATTAGCATCATTGGCACTTCTAACTGGAAACTTTGGAAGAC
CAAATGTAGGAATTGGTCCTGTAAGAGGCCAAAACAATGTACAAGGTGC
TTGTGATATGGGTGCACTCCCTAATGTATATCCAGGATATCAATCAGTAAC
AAATGATGCCATAAGAGAAAAATTTGAAAATGCTTGGGGAGTAAAACTTC
CAAATAAAGTGGGATATCACTTAACTGAAGTTCCTCATTTAGTGCTTAAAG
AGGATAAAATAAAAGCTTATTACATTATGGGTGAGGATCCAGTACAAAGT
GATCCAGATGCAGCAGAAGTTAGAGAAGCTTTGGACAAGTTAGAACTTG
TAATTGTTCAAGATATATTTATGAATAAAACAGCACTTCATGCAGATGTAAT
ATTACCAGCTACTTCTTGGGGTGAACATGAAGGTGTATATAGTTCGGCTGA
TAGAGGTTTCCAAAGATTCCGTAAGGCAATAGAACCTACAGGAGATGTAA
AGCCTGATTGGCAGATAATTTCAGAAATTGCTAAAGCTATGGGATATGACA
TGAATTATAAAAATACAAAGGAAATTTGGGATGAATTAAGAAACTTGTGT
CCAAACTTTAAGGGAGCAAGTTACGAAAGACTTGAAGAACTAGGTGGAA

TTCAATGGCCATGTCCTTCAGAAGATCATCCAGGAACTTCATATTTGTATA
AAGGAAATAAATTTAATACTCCTAGTGGAAAGGCAAATTTATTTGCAGCA
GAATGGAGAGCACCTATGGAGAGTACAGATAAGGAATACCCTCTAGTACT
TTCTACAGTAAGAGAGGTTGGTCACTATTCAGTAAGAACAATGACTGGTA
ACTGTAGGGCACTTCAGCAGTTAGCTGATGAGCCAGGTTATGTTCAAATA
AATCCAGAGGATGCTAAAAATTTAAATATATTAGATCAAGAATTTGTGAGA
ATAAGTTCACGTAGAGGATCTGTAGTAGCTAAGGCATTAGTTACAGATAG
AGTTAATAAAGGTGCAGTTTACATGACATACCAATGGTGGGTAGGAGCAT
GTAATGAGCTTACTTTAAATAACTTAGATCCTATATCTAAGACACCAGAAT
ATAAATATTGTGCTGTAAAGGTAGAAAATATTAAAGATCAAAAGGCTGCA
GAACAATATGTACAGGATGAGTATACTAAAATAAGAAAAAAGATGAACAT
AAATTTAGAATGTTGTAAATAG

PsFDH ATGGCAAAAGTTCTGTGCGTGCTGTATGATGATCCGGTTGATGGCTATCCG
AAAACCTATGCCCGTGATGATCTGCCGAAAATTGATCATTATCCGGGTGGT
CAGACCCTGCCGACCCCGAAAGCCATTGATTTTACCCCGGGCCAGCTGCT
GGGTAGTGTGAGTGGCGAACTGGGTCTGCGTAAATATCTGGAAAGTAATG
GTCATACCCTGGTTGTGACCAGTGATAAAGATGGTCCGGATAGTGTTTTT
GAACGCGAACTGGTTGATGCAGATGTGGTTATTAGCCAGCCGTTTTGGCC
GGCATATCTGACCCCGGAACGTATTGCCAAAGCAAAAAATCTGAAACTG
GCACTGACCGCCGGTATTGGCAGTGATCATGTTGATCTGCAGAGTGCCAT
TGATCGTAATGTTACCGTTGCAGAAGTGACCTATTGCAATAGTATTAGTGT
TGCAGAACATGTTGTTATGATGATTCTGAGTCTGGTTCGTAATTATCTGCC
GAGCCATGAATGGGCACGCAAAGGTGGTTGGAATATTGCCGATTGTGTGA
GCCATGCCTATGATCTGGAAGCAATGCATGTTGGTACCGTGGCAGCCGGC
CGCATTGGTCTGGCCGTGCTGCGTCGTCTGGCCCCTTTTGATGTTCATCTG
CATTATACCCAGCGTAATCGTCTGCCGGAAAGTGTTGAAAAAGAACTGAA
TCTGACCTGGCATGCCACCCGTGAAGATATGTATCCGGTGTGCGATGTTGT
GACCCTGAATTGTCCGCTGCATCCGGAAACCGAACACATGATTAATGATG
AAACCCTGAAACTGTTTAAGCGCGGTGCCTATATTGTGAATACCGCCCGT

	GGTAAACTGTGCGATCGTGATGCCGTGGCCCGTGCCCTGGAAAGCGGCA<br>GACTGGCAGGCTATGCAGGTGACGTGTGGTTTCCGCAGCCGGCACCGAA<br>AGATCATCCGTGGCGCACCATGCCGTATAATGGCATGACCCCGCATATTAG<br>CGGTACCACCCTGACCGCACAGGCCCGTTATGCCGCAGGTACCCGTGAA<br>ATTCTGGAATGCTTTTTCGAAGGTCGTCCGATTCGCGATGAATATCTGATT<br>GTTCAGGGTGGTGCACTGGCCGGTACCGGCGCACATAGCTATAGCAAAG<br>GTAATGCCACCGGCGGCAGCGAAGAAGCAGCCAAATTCAAAAAAGCAG<br>TGTAA
**GhrA**	ATGGATATCATCTTTTATCACCCAACGTTCGATACCCAATGGTGGATTGAG<br>GCACTGCGCAAAGCTATTCCTCAGGCAAGAGTCAGAGCATGGAAAAGCG<br>GAGATAATGACTCTGCTGATTATGCTTTAGTCTGGCATCCTCCTGTTGAAA<br>TGCTGGCAGGGCGCGATCTTAAAGCGGTGTTCGCACTCGGGGCCGGTGT<br>TGATTCTATTTTGAGCAAGCTACAGGCACACCCTGAAATGCTGAACCCTT<br>CTGTTCCACTTTTTCGCCTGGAAGATACCGGTATGGGCGAGCAAATGCAG<br>GAATATGCTGTCAGTCAGGTGCTGCATTGGTTTCGACGTTTTGACGATTAT<br>CGCATCCAGCAAAATAGTTCGCATTGGCAACCGCTGCCTGAATATCATCG<br>GGAAGATTTTACCATCGGCATTTTGGGCGCAGGCGTACTGGGCAGTAAAG<br>TTGCTCAGAGTCTGCAAACCTGGCGCTTTCCGCTGCGTTGCTGGAGTCGA<br>ACCCGTAAATCGTGGCCTGGCGTGCAAAGCTTTGCCGGACGGGAAGAAC<br>TGTCTGCATTTCTGAGCCAATGTCGGGTATTGATTAATTTGTTACCGAATA<br>CCCCTGAAACCGTCGGCATTATTAATCAACAATTACTCGAAAAATTACCG<br>GATGGCGCGTATCTCCTCAACCTGGCGCGTGGTGTTCATGTTGTGGAAGA<br>TGACCTGCTCGCGGCGCTGGATAGCGGCAAAGTTAAAGGCGCAATGTTG<br>GATGTTTTTAATCGTGAACCCTTACCGCCTGAAAGTCCGCTCTGGCAACA<br>TCCACGCGTGACGATAACACCACATGTCGCCGCGATTACCCGTCCCGCTG<br>AAGCTGTGGAGTACATTTCTCGCACCATTGCCCAGCTCGAAAAAGGGGA<br>GAGGGTCTGCGGGCAAGTCGACCGCGCACGCGGCTACTAA

Supplementary Table 14 | List of enzymes in this work.

Enzymes	Recognition site
BamHI	G^GATCC
SphI (PaeI)	GCATG^C
NdeI	CA^TATG
NotI	GC^GGCCGC
XhoI	C^TCGAG
HindIII	A^AGCTT
XbaI	T^CTAGA
T4 DNA Ligase	

Supplementary Table 15 | Strains and plasmids in this work.

Strains or plasmids	Genotype or Phenotype	Source
***A. vinelandii* strains**		
ATCC 9046	WT strain	ATCC
TCC 9046 nifD^{V70A}	ATCC 9046 in which the nifD^{V75A} mutation was introduced at its native locus using in vitro site-directed gene mutation technology	This Study
ATCC 9046 nifD^{H195G}	ATCC 9046 in which the nifD^{H195G} mutation was introduced at its native locus using in vitro site-directed gene mutation technology	This Study
ATCC 9046 nifD^{V70AH195G}	ATCC 9046 in which the nifD$^{V75A\ H195G}$mutation was introduced at its native locus using in vitro site-directed gene mutation technology	This Study
ATCC 9046 nifA*	nifA*; 48-bp dreeletion encoding Q-linker amino acids 202–217; Enhanced nifD expression	This Study
ATCC 9046nifA*nifD^{V70AH195G}	ATCC 9046 in which the nifD$^{V75A\ H195G}$mutation and nifA* was introduced at its native locus using in vitro site-directed gene mutation technology	This Study
***E. coli* strains**		
DH5α	F^{-}λ^{-}recA1Δ(lacZYA-argF) U169 hsdR17 thi-1 gyrA96 supE44 endA1 relA1 Φ80lacZΔM15	Gibco-BRL
BL21(DE)3	F- ompT hsdSB (rB- mB-) gal dcm (DE3)	Gibco-BRL
**Plasmids**		
pUC 19	pUC19 carries a 54 base-pair multiple cloning site polylinker that contains unique sites for 13 different hexanucleotide-specific restriction endonucleases, Ampicillin resistance.	Thermo
pUC 57-Kan	pUC57-Kan carries a 81 base-pair multiple cloning	MiaolingBio

	site polylinker that contains unique sites for 23 different hexanucleotide-specific restriction endonucleases, Kanamycin resistance.	
pUC 19-nifD	nifD coding sequence cloned into the MCS site of pUC 19	This Study
pUC 19-nifD^{V70A}	Mutant nifD^{V70A} coding sequence cloned into the MCS site of pUC 19	This Study
pUC 19-nifD^{H195G}	Mutant nifD^{H195G} coding sequence cloned into the MCS site of pUC 19	This Study
pUC 19-nifD^{V70AH195G}	Mutant nifD^{V70AH195G} coding sequence cloned into the MCS site of pUC 19	This Study
pUC 57-Kan-nifA*	nifA coding sequence cloned into the MCS site of pUC 57-Kan	This Study

Supplementary Table 16 | The compositions of *Azotobacter Vinelandii* medium (AVM) and *Escherichia coli* medium (ECM).

AVM	ECM
Yeast extract 5 g/L	Tryptone 10 g/L
Mannitol 20 g/L	Yeast extract 5 g/L
K_2HPO_4 0.2 g/L	NaCl 10 g/L
KH_2PO_4 0.8 g/L	Agar 15 g/L (Solid Medium)
Metal ion mixture*	NaOH adjust pH to 7.4
Agar 15 g/L (Solid Medium)	
NaOH adjust pH to 7.2	

*: $MgSO_4 \times 7H_2O$ 0.2 g/L, $CaSO_4 \times 2H_2O$ 0.1 g/L, $FeCl_3$ 0.01 g/L, $Na_2MoO_4 \times 2H_2O$ 0.002 g/L.

References

1. Bao, Y. P. et al. Immobilization of catalytic sites on quantum dots by ligand bridging for photocatalytic CO_2 reduction. *Nanoscale* **12,** 2507-2514 (2020).
2. Wang, J. et al. Enabling Visible-Light-Driven Selective CO_2 Reduction by Doping Quantum Dots: Trapping Electrons and Suppressing H_2 Evolution. *Angew. Chem. Int. Ed.* **57,** 16447-16451 (2018).
3. Wang, Y. & Ni, Y. N. Molybdenum Disulfide Quantum Dots as a Photoluminescence Sensing Platform for 2,4,6-Trinitrophenol Detection. *Anal. Chem.* **86,** 7463-7470 (2014).
4. Peng, H., Zhang, L. J., Kjallman, T. H. M., Soeller, C. & Travas-Sejdic, J. DNA hybridization detection with blue luminescent quantum dots and dye-labeled single-stranded DNA. *J. Am. Chem. Soc.* **129,** 3048-3049 (2007).
5. Miller, T. E. et al. Light-powered CO_2 fixation in a chloroplast mimic with natural and synthetic parts. *Science* **368,** 649-654 (2020).
6. Li, F. et al. Installing a Green Engine To Drive an Enzyme Cascade: A Light-Powered In Vitro Biosystem for Poly(3-hydroxybutyrate) Synthesis. *Angew. Chem. Int. Ed.* **61,** e202111054 (2022).
7. Chen, Z. W. et al. Light-Gated Synthetic Protocells for Plasmon-Enhanced Chemiosmotic Gradient Generation and ATP Synthesis. *Angew. Chem. Int. Ed.* **58,** 4896-4900 (2019).
8. Porra, R. J. The chequered history of the development and use of simultaneous equations for the accurate determination of chlorophylls a and b. *Photosyn. Res.* **73,** 149-156 (2002).
9. Wittig, I., Braun, H. P. & Schagger, H. Blue native PAGE. *Nat. Protoc.* **1,** 418-428 (2006).
10. Lee, K. Y. et al. Photosynthetic artificial organelles sustain and control ATP-dependent reactions in a protocellular system. *Nat. Biotechnol.* **36,** 530-535 (2018).
11. Kuruma, Y. & Ueda, T. The PURE system for the cell-free synthesis of membrane proteins. *Nat. Protoc.* **10,** 1328-1344 (2015).
12. Berhanu, S., Ueda, T. & Kuruma, Y. Artificial photosynthetic cell producing energy for protein synthesis. *Nat. Commun.* **10,** 1325 (2019).
13. Tian, W. J. et al. Photoelectrochemical Water Oxidation and Longevous Photoelectric Conversion by a Photosystem II Electrode. *Adv. Energy Mater.* **11,** 2100911 (2021).
14. Zhao, Y. J. et al. Fully Conjugated Two-Dimensional sp^2-Carbon Covalent Organic Frameworks as Artificial Photosystem I with High Efficiency. *Angew. Chem. Int. Ed.* **58,** 5376-5381 (2019).
15. Yang, Y. et al. Structural basis for human ZBTB7A action at the fetal globin promoter. *Cell Rep.* **36,**

109759 (2021).

16. Ihara, M., Kawano, Y., Urano, M. & Okabe, A. Light Driven CO_2 Fixation by Using Cyanobacterial Photosystem I and NADPH-Dependent Formate Dehydrogenase. *PLoS One* **8,** e71581 (2013).
17. Kuk, S. K. et al. Photoelectrochemical Reduction of Carbon Dioxide to Methanol through a Highly Efficient Enzyme Cascade. *Angew. Chem. Int. Ed.* **56,** 3827-3832 (2017).
18. Yang, Z. Y., Moure, V. R., Dean, D. R. & Seefeldt, L. C. Carbon dioxide reduction to methane and coupling with acetylene to form propylene catalyzed by remodeled nitrogenase. *Proc. Natl. Acad. Sci. U.S.A.* **109,** 19644-19648 (2012).
19. Shah, V. K., Brill, W. J. & Davis, L. C. Nitrogenase .I. Repression and Derepression of Iron-Molybdenum and Iron Proteins of Nitrogenase in Azotobacter-Vinelandii. *Biochim. Biophys. Acta* **256,** 498-511 (1972).
20. Chowdhury, D. R., Spiccia, L., Amritphale, S. S., Paul, A. & Singh, A. A robust iron oxyhydroxide water oxidation catalyst operating under near neutral and alkaline conditions. *J. Mater. Chem. A* **4,** 3655-3660 (2016).
21. Zhao, Z. F. et al. Engineering Olefin-Linked Covalent Organic Frameworks for Photoenzymatic Reduction of CO_2. *Angew. Chem. Int. Ed.* **61,** e202200261 (2022).
22. Wang, Y. J. et al. Wafer-scale synthesis of monolayer WSe_2: A multi-functional photocatalyst for efficient overall pure water splitting. *Nano Energy* **51,** 54-60 (2018).
23. Qorbani, M. et al. Atomistic insights into highly active reconstructed edges of monolayer 2H-WSe_2 photocatalyst. *Nat. Commun.* **13,** 1256 (2022).
24. García de Arquer, F. P. et al. Semiconductor quantum dots: Technological progress and future challenges. *Science* **373,** eaaz8541 (2021).
25. Mondal, N. & Samanta, A. Ultrafast Charge Transfer and Trapping Dynamics in a Colloidal Mixture of Similarly Charged CdTe Quantum Dots and Silver Nanoparticles. *J. Phys. Chem. C* **120,** 650-658 (2016).
26. Xiao, J.-W. et al. Ligand engineering on CdTe quantum dots in perovskite solar cells for suppressed hysteresis. *Nano Energy* **46,** 45-53 (2018).
27. Soll, J. & Schleiff, E. Protein import into chloroplasts. *Nat. Rev. Mol. Cell Biol.* **5,** 198-208 (2004).
28. Hu, C. M. J. et al. Nanoparticle biointerfacing by platelet membrane cloaking. *Nature* **526,** 118-121 (2015).

29. Hu, C. M. J., Fang, R. H., Luk, B. T. & Zhang, L. F. Nanoparticle-detained toxins for safe and effective vaccination. *Nat. Nanotechnol.* **8,** 933-938 (2013).

30. Reszczyńska, E. & Hanaka, A. Lipids Composition in Plant Membranes. *Cell Biochem. Biophys.* **78,** 401-414 (2020).

31. Krumova, S. B. et al. Temperature dependence of the lipid packing in thylakoid membranes studied by time- and spectrally resolved fluorescence of Merocyanine 540. *Biochim. Biophys. Acta* **1778,** 2823-2833 (2008).

32. Pannwitz, A. et al. Roadmap towards solar fuel synthesis at the water interface of liposome membranes. *Chem. Soc. Rev.* **50,** 4833-4855 (2021).

33. Zhu, X. G., Long, S. P. & Ort, D. R. Improving photosynthetic efficiency for greater yield. *Annu. Rev. Plant Biol.* **61,** 235-261 (2010).

34. Bolton, J. R. & Hall, D. O. Photochemical Conversion and Storage of Solar Energy. *Annu. Rev. Energy* **4,** 353-401 (1979).

35. Kornienko, N., Zhang, J. Z., Sakimoto, K. K., Yang, P. D. & Reisner, E. Interfacing nature's catalytic machinery with synthetic materials for semi-artificial photosynthesis. *Nat. Nanotechnol.* **13,** 890-899 (2018).

36. Ben-Hayyim, G., Drechsler, Z. & Neumann, J. Photosystem 2 mediated electron transport and phosphorylation with ferricyanide and dibromothymoquinone the uncoupling activity of dibromothymoquinone. *Plant Sci. Lett.* **7,** 171-178 (1976).

37. Trebst, A. Energy Conservation in Photosynthetic Electron Transport of Chloroplasts. *Ann. Rev. Plant Physiol.* **25,** 423-458 (1974).

38. Wang, W. Y., Chen, J., Li, C. & Tian, W. M. Achieving solar overall water splitting with hybrid photosystems of photosystem II and artificial photocatalysts. *Nat. Commun.* **5,** 4647 (2014).

39. Kato, M., Zhang, J. Z., Paul, N. & Reisner, E. Protein film photoelectrochemistry of the water oxidation enzyme photosystem II. *Chem. Soc. Rev.* **43,** 6485-6497 (2014).

40. Tschörtner, J., Lai, B. & Krömer, J. O. Biophotovoltaics: Green Power Generation From Sunlight and Water. *Front. Microbiol.* **10,** 866 (2019).

41. Tu, Y.-J., Njus, D. & Schlegel, H. B. A theoretical study of ascorbic acid oxidation and $HOO^{\cdot}/O_2^{\cdot-}$ radical scavenging. *Org. Biomol. Chem.* **15,** 4417-4431 (2017).

42. Martini, M. A. et al. The Nonphysiological Reductant Sodium Dithionite and [FeFe] Hydrogenase:

Influence on the Enzyme Mechanism. *J. Am. Chem. Soc.* **143,** 18159-18171 (2021).

43. Yang, Z.-Y., Moure, V. R., Dean, D. R. & Seefeldt, L. C. Carbon dioxide reduction to methane and coupling with acetylene to form propylene catalyzed by remodeled nitrogenase. *Proc. Natl. Acad. Sci. U.S.A.* **109,** 19644-19648 (2012).
44. Yang, Z.-Y., Badalyan, A., Hoffman, B. M., Dean, D. R. & Seefeldt, L. C. The Fe Protein Cycle Associated with Nitrogenase Catalysis Requires the Hydrolysis of Two ATP for Each Single Electron Transfer Event. *J. Am. Chem. Soc.* **145,** 5637-5644 (2023).
45. Chen, Y. J. et al. Integration of Enzymes and Photosensitizers in a Hierarchical Mesoporous Metal-Organic Framework for Light-Driven CO_2 Reduction. *J. Am. Chem. Soc.* **142,** 1768-1773 (2020).
46. Kim, J. et al. Robust FeOOH/$BiVO_4$/Cu(In, Ga)Se_2 tandem structure for solar-powered biocatalytic CO_2 reduction. *J. Mater. Chem. A* **8,** 8496-8502 (2020).
47. Tian, Y. et al. Construction of Functionally Compartmental Inorganic Photocatalyst-Enzyme System via Imitating Chloroplast for Efficient Photoreduction of CO_2 to Formic Acid. *ACS Appl. Mater. Interfaces* **12,** 34795-34805 (2020).
48. Zhang, S. H. et al. Artificial Thylakoid for the Coordinated Photoenzymatic Reduction of Carbon Dioxide. *ACS Catal.* **9,** 3913-3925 (2019).
49. Meng, J. L. et al. A thiophene-modified doubleshell hollow g-C_3N_4 nanosphere boosts NADH regeneration via synergistic enhancement of charge excitation and separation. *Catal. Sci. Technol.* **9,** 1911-1921 (2019).
50. Shah, K. J. & Imae, T. Photoinduced enzymatic conversion of CO_2 gas to solar fuel on functional cellulose nanofiber films. *J. Mater. Chem. A* **5,** 9691-9701 (2017).
51. Son, E. J. et al. Sunlight-assisted, biocatalytic formate synthesis from CO_2 and water using silicon-based photoelectrochemical cells. *ChemComm* **52,** 9723-9726 (2016).
52. Yadav, D., Yadav, R. K., Kumar, A., Park, N. J. & Baeg, J. O. Functionalized Graphene Quantum Dots as Efficient Visible-Light Photocatalysts for Selective Solar Fuel Production from CO_2. *ChemCatChem* **8,** 3389-3393 (2016).
53. Nam, D. H. et al. Enzymatic photosynthesis of formate from carbon dioxide coupled with highly efficient photoelectrochemical regeneration of nicotinamide cofactors. *Green Chem.* **18,** 5989-5993 (2016).
54. Ji, X. Y., Su, Z. G., Wang, P., Ma, G. H. & Zhang, S. P. Integration of Artificial Photosynthesis System

for Enhanced Electronic Energy-Transfer Efficacy: A Case Study for Solar-Energy Driven Bioconversion of Carbon Dioxide to Methanol. *Small* **12,** 4753-4762 (2016).

55. Yadav, R. K. et al. Highly Selective Solar-Driven Methanol from CO_2 by a Photocatalyst/Biocatalyst Integrated System. *J. Am. Chem. Soc.* **136,** 16728-16731 (2014).
56. Yadav, R. K. et al. Graphene-BODIPY as a photocatalyst in the photocatalytic-biocatalytic coupled system for solar fuel production from CO_2. *J. Mater. Chem. A* **2,** 5068-5076 (2014).
57. Yadav, R. K. et al. A Photocatalyst-Enzyme Coupled Artificial Photosynthesis System for Solar Energy in Production of Formic Acid from CO_2. *J. Am. Chem. Soc.* **134,** 11455-11461 (2012).
58. Amao, Y. & Ishikawa, M. Visible light and enzymatic induced synthesis of malic acid from pyruvic acid and HCO_3^- with the combination system of zinc chlorophyll derivative and malic enzyme in water media. *Catal. Commun* **8,** 523-526 (2007).
