# Supplementary material for: Artificial photosynthetic cells with biotic–abiotic hybrid energy modules for customized CO2 conversion
Source: Nat Commun. 2023 Oct 25;14:6783. doi: 10.1038/s41467-023-42591-x (PMC10600252; doi:10.1038/s41467-023-42591-x)
Supplement: Supplementary file 4 — Description of Additional Supplementary files [file 41467_2023_42591_MOESM4_ESM.docx]

**Supplementary Movie 1**

This file contains Supplementary Movie 1. Microdroplets with ~100 µm in diameter and distinctive rough edge were generated by a flow-focusing junction fabricated in a polydimethylsiloxane chip. The desired artificial photosynthetic cells were successfully constructed. Upon the fabrication of artificial cells, the activity of NADH regeneration was assessed in the cells. Tk-CdTe group indicated Buffer E + Tk-CdTe. The movie was displayed at a rate of 24 frames/sec.

**Supplementary Movie 2**

This file contains Supplementary Movie 2. Microdroplets with ~100 µm in diameter and distinctive rough edge were generated by a flow-focusing junction fabricated in a polydimethylsiloxane chip. Upon the fabrication of artificial cells, the activity of NADH-dependent photoenzymatic CO_2_ reduction was assessed via formate production with CcFDH in the cells. Tk-CdTe group indicated Buffer G + Tk-CdTe + CcFDH. The movie was displayed at a rate of 24 frames/sec.

**Supplementary Movie 3**

This file contains Supplementary Movie 3. Microdroplets with ~100 µm in diameter and distinctive rough edge were generated by a flow-focusing junction fabricated in a polydimethylsiloxane chip. Upon the fabrication of artificial cells, the activity of NADPH and ATP regeneration was assessed in the cells. Tk-CdTe group indicated Buffer F + Tk-CdTe. The movie was displayed at a rate of 24 frames/sec.
